# Correction: A New Large Hyainailourine from the Bartonian of Europe and Its Bearings on the Evolution and Ecology of Massive Hyaenodonts (Mammalia)

**DOI:** 10.1371/journal.pone.0141941

**Published:** 2015-10-30

**Authors:** The PLOS ONE Staff

## Notice of Republication

This article was republished on October 14, 2015, to correct the order of Figs 8–13. The publisher apologizes for the error. Please download this article again to view the correct version. The originally published, uncorrected article and the republished, corrected article are provided here for reference.

## Supporting Information

S1 File Originally published, uncorrected article. (PDF)

S2 File Republished corrected article. (PDF)
