# Supplementary material for: A New Large Hyainailourine from the Bartonian of Europe and Its Bearings on the Evolution and Ecology of Massive Hyaenodonts (Mammalia)
Source: PLoS One. 2015 Sep 23;10(9):e0135698. doi: 10.1371/journal.pone.0135698 (PMC4580617; doi:10.1371/journal.pone.0135698)
Supplement: S1 Text — *Category established by comparisons with relatives of similar sizes. (DOCX) [file pone.0135698.s001.docx]

**Text S1.** **Taxa included in the phylogenetic analysis – except *Parvavorodon* – and their estimated body mass.** *Category established by comparisons with relatives of similar sizes.

| **Taxon** | **Estimated body mass (state for character 1)** | **Age** | **Repartition** | **References** |
| --- | --- | --- | --- | --- |
| *Tinerhodon disputatus* | 0.02 kg (0) | Late Paleocene | Africa | [10] |
| **Sinopinae** | | | | |
| *Prototomus minimus / P. girardoti* | 0.24 / 0.45 kg (0) | Early Eocene | Europe | [13-14] |
| **Proviverrinae** | | | | |
| *Eoproviverra / Parvagula /*  *Proviverra* | 0.031 / 0.064 / 0.42 kg (0) | Early - Late Eocene | Europe | [18-19] |
| **Koholiinae** | | | | |
| *Lahimia selloumi* | 0.81 kg (0) | Late Paleocene | Africa | [1] |
| *Boualitomus marocanensis* | 0.24 kg (0) | Early Eocene | Africa | [8] |
| **Hyainailourinae** | | | | |
| *Akhnatenavus leptognathus* | 30.31 kg (2) | Late Eocene | Africa | [48] |
| *Furodon crocheti* | 1.88 kg (0) | Middle Eocene | Africa | [11] |
| *Hemipsalodon* | 271.15 (4) | Middle-Late Eocene | North America | [41,85] |
| *Kerberos langebadreae* | 87.69 kg (3) | Middle Eocene | Europe | Present paper |
| *Hyainailouros* *bugtiensis (=Megistotherium osteothlastes)* | 2027.53 kg (4) | Early-Middle Miocene | Africa | [87] |
| *Hyainailouros sulzeri* | 1109.92 kg (4) | Early-Middle Miocene | Africa and Europe | [51] |
| *Isohyaenodon andrewsi* | 14.26 kg (2) | Early Miocene | Africa | [3] |
| *Leakitherium hiwegi** | (2) | Early Miocene | Africa | [3] |
| *Isohyaenodon pilgrimi* | 0.56 kg (0) | Early Miocene | Africa | [3] |
| *Isohyaenodon zadoki* | 7.35 kg (1) | Early Miocene | Africa | [3] (M_1_ and M_2_ estimated after *I. andrewi* M.15048) |
| *Parapterodon lostangensis* | 97.78 kg (3) | Late Eocene | Europe | [24] |
| *Paroxyaena galliae* | 46.71 kg (3) | Middle Eocene | Europe | [24] |
| *Paroxyaena pavlovi** | Close in size to *Paroxyaena galliae* (3) | Late Eocene | Europe | [53] |
| *“Pterodon” africanus* | 253.45 kg (4) | Late Eocene | Africa | [48] |
| *Pterodon dasyuroides* | 51.56 kg (3) | Late Eocene | Europe | [24] |
| *“Pterodon” phiomensis* | 146.59 kg (4) | Late Eocene | Africa | [48] |
| *“Pterodon” styros** | Close in size to *Pterodon dasyuroides* (3) | Early Oligocene | Africa | [48] |
| S*ivapterodon lahirii* | Close in size to *Hyainailouros* (4) | Middle Miocene | Asia | [34] |
| *Parvavorodon gheerbranti** | Close in size to *Boualitomus marocanensis* (0) | ?Early Eocene | Africa | [11] |
| **Apterodontinae** | | | | |
| *Apterodon langebadreae* | 14.54 kg (2) | Middle Eocene | Africa | [35] |
| *Apterodon macrognathus* | 38.86 kg (3) | Late Eocene | Africa | [35] |
