# Supplementary material for: A New Large Hyainailourine from the Bartonian of Europe and Its Bearings on the Evolution and Ecology of Massive Hyaenodonts (Mammalia)
Source: PLoS One. 2015 Sep 23;10(9):e0135698. doi: 10.1371/journal.pone.0135698 (PMC4580617; doi:10.1371/journal.pone.0135698)
Supplement: S2 Text — (DOCX) [file pone.0135698.s002.docx]

**Text S2. Character list.**

1. **Body size**

Very small (<1 kg) **(0)**; small (<11 kg) **(1)**; medium (<31 kg) **(2)**; large (<100 kg) **(3)**; very large (> 100kg) **(4)**.

1. **Incisor number**

Unreduced (3) **(0)**; reduced (< 3) **(1)**.

1. **P^1^**

Double-rooted **(0)**; single-rooted **(1)**.

1. **Premolar buccal cingulum**

Absent/weak **(0)**; strong **(1)**.

1. **P^3^ lingual cingulum**

Weak or absent **(0)**; with large lingual cingulum **(1)**; strong with salient basal lingual expansion **(2)**

1. **P^3^ metastyle**

Present **(0)**; very reduced to absent **(1)**.

1. **P^3^ protocone**

Present **(0)**; absent **(1)**.

1. **P^3^ roots**

Three roots **(0)**; two roots **(1)**.

1. **P^4^ metastyle**

Present **(0)**; absent **(1)**.

1. **P^4^ parastyle**

Present, moderate **(0)**; expanded **(1)**; small or absent **(2)**.

1. **P^4^ distal accessory cusp**

Not bulbous **(0)**; bulbous **(1)**.

1. **P^4^ postparacrista**

Not shearing **(0)**; shearing **(1)**.

1. **P^4^ protocone**

Present **(0)**; small to absent **(1)**.

1. **P^4^ roots**

Mesial is smallest **(0)**; distal is smallest **(1)**.

1. **P^4^-M^2^ mesiolabial cingulum**

Present **(0)**; vestigial **(1)**; absent **(2)**.

1. **Molar paraconule crista**

Strong **(0)**; weak and short **(1)**.

1. **M^1^-M^2^ metaconule**

Present **(0)**; absent **(1)**.

1. **M^1^-M^2^ metastyle length**

Short **(0)**; medium **(1)**; long **(2)**

1. **M^1^-M^2^ paracone/metacone fusion**

Completely separated **(0)**; fused at base **(1)**; fused with groove **(2)**; fused without groove **(3)**.

1. **M^1^-M^2^ paracone height**

Greater than metacone **(0)**; subequal to metacone **(1)**.

1. **M^1^-M^2^ paraconule**

Present **(0)**; absent **(1)**

1. **M^1^-M^2^ parastyle form**

Present **(0)**; absent on M^1^ or on M^1^ and M^2^ **(1)**.

1. **M^1^-M^2^ parastyle size**

Well-developed **(0)**; short **(1)**.

1. **M^1^-M^2^ protocone size**

Large **(0)**; small **(1)**; absent **(2)**.

1. **M^1^-M^2^ ectoflexus**

Absent **(0)**; single-pronounced **(1)**.

1. **M^1^-M^2^ protocone position**

Lingual **(0)**; mesiolingual **(1)**

1. **M^3^**

Unreduced **(0)**; two-cusped **(1)**; lost **(2)**.

1. **P_1_**

Unreduced **(0)**; reduced **(1)**; absent **(2)**.

1. **P_2_/P_3_ size relative to P_4_**

Slightly smaller **(0)**; smaller very reduced **(1)**; very reduced **(2)**.

1. **P_3_ mesial cuspulid**

Present **(0)**; absent **(1)**

1. **P_3_ distal cuspulid**

Present **(0)**; absent **(1)**.

1. **P_4_ mesial cuspulid**

Present **(0)**; absent **(1)**.

1. **P_4_ shape**

Low and broad **(0)**; tall and short **(1)**; tall and long **(2)**.

1. **P_4_ talonid**

Present **(0)**; absent **(1)**.

1. **Premolar diastemata**

Large **(0)**; slight **(1)**; none **(2)**.

1. **Lower molar series relative to premolar series length**

Premolar > molar **(0)**; molar > premolar **(1)**; subequal **(2)**.

1. **M_1_ metaconid**

High or as high as paraconid **(0)**; lower than paraconid **(1)**; absent **(2)**.

1. **M_1_ relative to M_2_**

>50% **(0)**; almost <50% **(1)**.

1. **M_1_ talonid basin**

Present **(0)**; absent **(1)**.

1. **M_1_ talonid entoconid**

Present **(0)**; reduced (crestiform) **(1)**; absent **(2)**.

1. **M_1_ talonid hypoconulid**

Present **(0)**; absent **(1)**.

1. **M_1_-M_3_ anterior keel**

Relatively small **(0)**; relatively large **(1)**.

1. **M_1_-M_2_ talonid size**

Moderate **(0)**; small **(1)**.

1. **M_1_-M_2_ trigonid/talonid width**

Subequal **(0)**; talonid narrower than trigonid **(1)**.

1. **M_2_ talonid length (ordered)**

1/3 of tooth **(0)**; > 1/3 tooth **(1)**; vestigial **(2)**.

1. **M_2_/M_3_ paraconid height**

Lower than protoconid **(0)**; subequal to protoconid **(1)**.

1. **M_2_/M_3_ protoconid to paraconid length**

Subequal **(0)**; protoconid > paraconid **(1)**.

1. **M2/M3 postprotocritid**

Directed to metaconid **(0)**; distally directed **(1)**; distally directed and elongated **(2)**.

1. **M_3_ talonid**

Present, basined **(0)**; present, trenchant **(1)**; absent **(2)**.

1. **Symphyseal region**

Unfused **(0)**; fused **(1)**.
