# Supplementary material for: Correction: A New Large Hyainailourine from the Bartonian of Europe and Its Bearings on the Evolution and Ecology of Massive Hyaenodonts (Mammalia)
Source: PLoS One. 2015 Oct 30;10(10):e0141941. doi: 10.1371/journal.pone.0141941 (PMC4627799; doi:10.1371/journal.pone.0141941)
Supplement: S1 File — (PDF) [file pone.0141941.s001.pdf]

PLOS ONE

RESEARCH ARTICLE

A New Large Hyainailourine from the Bartonian of Europe and Its Bearings on the Evolution and Ecology of Massive Hyaenodonts (Mammalia)

Floréal Solé[1,2]*, **Eli Amson**[2], **Matthew Borths**[3], **Dominique Vidalenc**[4], **Michael Morlo**[5], **Katharina Bastl**[6,7]

1 D. O. Earth and history of Life, Department of Paleontology, Royal Belgian Institute of Natural Sciences, Rue Vautier 29, B-1000, Brussels, Belgium, **2** Centre de Recherche sur la Paléobiodiversité et les Paléoenvironnements (CR2P: CNRS, MNHN, UPMC-Paris-06, Sorbonne Universités), Muséum national d'Histoire Naturelle, département Histoire de la Terre, 57 rue Cuvier, CP38, F-75005, Paris, France, **3** Department of Anatomical Sciences, Stony Brook University, Stony Brook, New York, 11794, United States of America, **4** Independent Researcher, 103 avenue F. Mitterrand, 31800, St Gaudens, France, **5** Forschungsinstitut Senckenberg, Senckenberganlage 25, D-60235, Frankfurt, Germany, **6** Institut für Paläontologie, Universität Wien, Althanstraße 14, A-1090, Wien, Österreich, **7** HNO-Klinik, Medizinische Universität Wien, Forschungsgruppe Aerobiologie und Polleninformation, Währinger Gürtel 18–20, A-1090, Wien, Österreich

* floreal.sole@naturalsciences.be

CrossMark
click for updates

OPEN ACCESS

Citation: Solé F, Amson E, Borths M, Vidalenc D, Morlo M, Bastl K (2015) A New Large Hyainailourine from the Bartonian of Europe and Its Bearings on the Evolution and Ecology of Massive Hyaenodonts (Mammalia). PLoS ONE 10(9): e0135698. doi:10.1371/journal.pone.0135698

Editor: Matt Friedman, University of Oxford, UNITED KINGDOM

Received: January 8, 2015

Accepted: July 26, 2015

Published: September 23, 2015

Copyright: © 2015 Solé et al. This is an open access article distributed under the terms of the Creative Commons Attribution License, which permits unrestricted use, distribution, and reproduction in any medium, provided the original author and source are credited.

Data Availability Statement: All relevant data are within the paper and its Supporting Information files.

Funding: This research was supported by the National Science Foundation Grant DEB-1311354 to MB (PhD Thesis). The Belgian Science Policy Office supports the project Paleurafrica BR/121/A3/ PALEURAFRICA (Postdoctoral position of FS). The funders had no role in study design, data collection and analysis, decision to publish, or preparation of the manuscript.

Abstract

We describe a new large-sized species of hypercarnivorous hyainailourine–*Kerberos langebadreae* gen. & sp. nov.–from the Bartonian (MP16) locality of Montespieu (Tarn, France). These specimens consist of a skull, two hemimandibles and several hind limb elements (fibula, astragalus, calcaneum, metatarsals, and phalanges). Size estimates suggest *K*. *langebadreae* may have weighed up to 140 kg, revealing this species as the largest carnivorous mammal in Europe at that time. Besides its very large size, *K*. *langebadreae* possesses an interesting combination of primitive and derived features. The distinctive skull morphology of *K*. *langebadreae* reflects a powerful bite force. The postcranial elements, which are rarely associated with hyainailourine specimens, indicate an animal capable of a plantigrade stance and adapted for terrestrial locomotion. We performed the first phylogenetic analysis of hyainailourines to determine the systematic position of *K*. *langebadreae* and to understand the evolution of the group that includes other massive carnivores. The analysis demonstrates that *Hemipsalodon*, a North American taxon, is a hyainailourine and is closely related to European *Paroxyaena*. Based on this analysis we hypothesize the biogeographic history of the Hyainailourinae. The group appeared in Africa with a first migration to Europe during the Bartonian that likely included the ancestors of *Kerberos*, *Paroxyaena* and *Hemipsalodon*, which further dispersed into North America at this time. We propose that the hyainailourines dispersed into Europe also during the Priabonian. These migrants have no ecological equivalent in Europe during these intervals and likely did not conflict with the endemic hyaenodont proviverrines. The discovery of *K*. *langebadreae*

Competing Interests: The authors have declared that no competing interests exist.

shows that large body size appears early in the evolution of hyainailourines. Surprisingly, the late Miocene *Hyainailouros* shares a more recent common ancestor with small-bodied hyainailourines (below 15 kg). Finally, our study supports a close relationship between the Hyainailourinae and Apterodontinae and we propose the new clade: Hyainailouridae.

Introduction

Hyaenodonta is an order of specialized carnivorous mammals that is known from the Selandian (Paleocene) [1–2] to the Serravallian (Miocene) [3–4]. Hyaenodonta probably originated either in Africa [1,5–6] or in Asia [7]. Until now, two Palaeocene species have been recorded in Africa: *Tinerhodon disputatus* Gheerbrant, 1995 [5] and *Lahimia selloumi* Solé & Gheerbrant, 2009 in Solé et al. [1]. The sole Laurasian hyaenodont recorded in the Paleocene is *Prolimnocyon chowi* Meng, Zhai & Wyss, 1998 [7] from China. Species from this order are recorded in Africa, India, and all of Laurasia (Asia, Europe, North America).

Hyaenodonts are first recorded in Europe during the earliest Eocene (Fig 1). Three subfamilies are known in Europe at this time. Sinopinae and Arfiinae were diverse in Northern Europe and found in Dormaal (Belgium; MP7) [12], Le Quesnoy (France; MP7) [13], and Abbey Wood (England; MP8+9) [14], while the Proviverrinae were restricted to Southern European Province [15–17] (as defined by Marandat [18] and Marandat et al. [19]). During the main part of the Ypresian, Lutetian, and Bartonian the hyaenodont proviverrines were the dominant specialized carnivorous mammals in European ecosystems [17,20–22] (Fig 1). The diversification of proviverrines in Europe, which were small-bodied and restricted to southern Europe during the earliest Eocene, was spurred by the disappearance of the subfamily's presumed ecological competitors–Oxyaenodonta, and hyaenodonts from the clades Sinopinae and Arfiinae–during the Ypresian (between Dormaal (reference-level MP7) and Avenay (reference-level MP8+9) [13,17,23]).

The decline of the proviverrines occurred during the Priabonian and coincides with the arrival in Europe during Bartonian and Priabonian of new competitors from Asia (Carnivora and Hyaenodontinae) and Africa (Hyainailourinae) [9] (Fig 1). The last occurrence of Proviverrinae (*Allopterodon minor*) is close to the MP19 reference-level in Obergösgen (Priabonian; Germany) [24]. While the Bartonian is characterized by the presence of Proviverrinae and Hyainailourinae, the Priabonian of Europe is characterized by the presence of Proviverrinae, Hyainailourinae and Hyaenodontinae (Fig 1).

One of the co-authors, D.V., discovered in 1981 the specimens described here in the French site of Montespieu–a locality that is very close to the city of Lautrec (Tarn) (Fig 2). As part of the "*Castrais*" faunas, the locality is considered to be Bartonian in age and close to the MP16 level (Robiac) [26–29]. The fossils belong to a large hyaenodont referred to a new hyainailourine taxon: *Kerberos langebadreae* gen. & sp. nov. The new taxon described here represents one of the two oldest hyainailourines recorded in Europe. The other, the hyainailourine *Paroxyaena*, is known in Robiac's fauna (reference locality of MP16 reference-level) [30]. The study of these hyainailourines is important for understanding their ecological role in the Bartonian of Europe just as proviverrines begin to decline (Fig 1). Moreover, *Kerberos langebadreae* expands our knowledge of carnivorous mammalian niches in European environments.

The discovery of this new species also has important implications for the evolution of Hyainailourinae. Solé et al. [6] recently described the earliest hyainailourines from the Gour Lazib

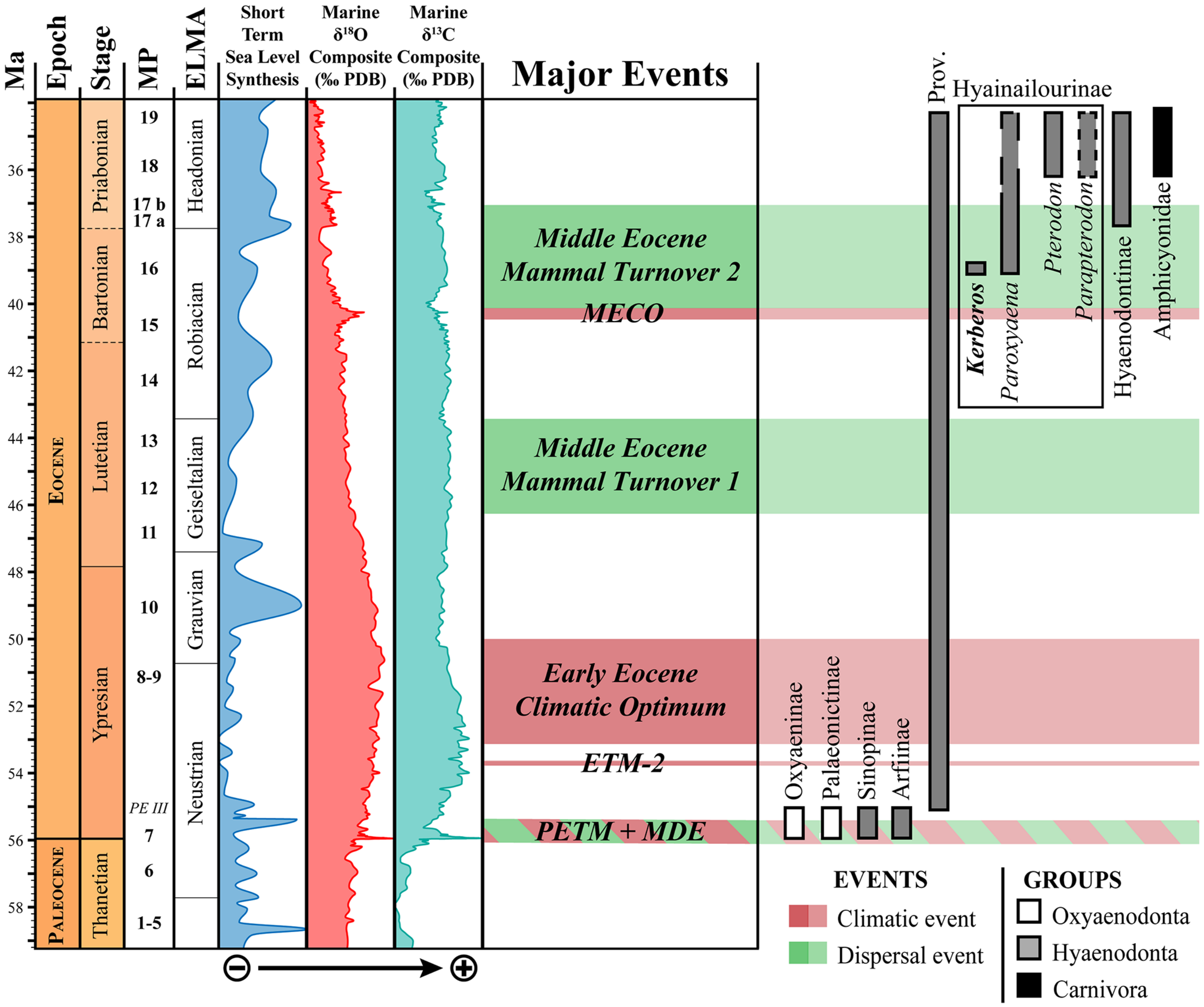

Fig 1. Stratigraphic repartition of the different subfamilies of specialized carnivorous mammals in Europe during the Paleocene and Eocene, with special attention to hyainailourines. The new taxon described here, *Kerberos langebadreae*, is in bold. Note that currently i. the stratigraphic extension of *Paroxyaena pavlovi* during Priabonian is unknown [8] and ii. the age of *Parapterodon* is unknown but is estimated to be late Priabonian [9]. Stratigraphic scale, eustatic curve, and isotopic curves produced with TSCreator [10] from the data compiled by Gradstein et al. [11]. Abbreviations: ELMA, European Land Mammal Ages; MP, Mammal Palaeogene.

doi:10.1371/journal.pone.0135698.g001

Formation (late Early or early Middle Eocene; Algeria). Through subsequent dispersals, this subfamily is known from Africa, Asia, Europe, and North America, but the phylogenetic hypothesis presented in Solé et al. [6] supports an African origin for Hyainailourinae. This new taxon allows i. the study of the evolution of this subfamily, and ii. the examination of hyainailourine dispersals into Laurasia during the Eocene.

Additionally, the cranial, mandibular and dental remains associated with postcranial material are the first known for an early hyainailourine. Because the new taxon is one of the oldest hyainailourines ever recorded, the postcranial material allows us to study the skeletal morphology and locomotion of an early representative of the subfamily.

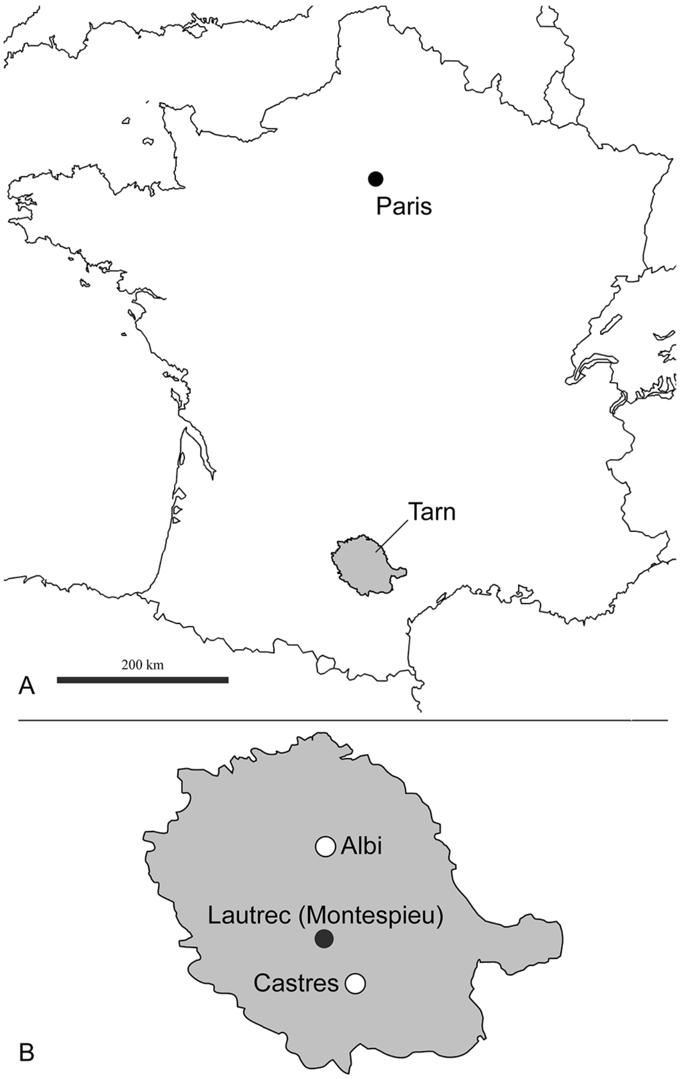

Fig 2. Geographic map with indications of the location of the fossiliferous locality of Montespieu (MP16), where material of *Kerberos langebadreae* was found. A, Localisation of the department of Tarn (France). **B,** Localisation of Montespieu in Tarn. B: redrawn after Laurent et al. ([25], Fig 1).

doi:10.1371/journal.pone.0135698.g002

Material and Methods

Material

The fossils of *Kerberos langebadreae* were found by D.V. in the French fossiliferous locality of Montespieu, which is situated close to Lautrec (Tarn, France) (Fig 2). The Montespieu locality was discovered during 19th century. The mammals that were found in this locality were notably described and illustrated by Stehlin [26]. Most of the first fossils collected from the locality are housed in the Muséum d'Histoire naturelle de Toulouse (Collection Noulet). The mammal fauna of Montespieu is considered close to that of Robiac in age–the latter locality is the reference-locality for reference-level MP16 [29,30].

The fieldwork undertaken by D.V. in 1970s and '80s previously provided the type and only specimen of *Cynohyaenodon lautricensis* [31].

The material of the new hyainailourine was prepared by D.V. and C. Bouillet (MNHN). The specimens are housed at the *Muséum national d'Histoire naturelle* (Paris, France).

Specimen numbers: MNHN.F.EBA 517; MNHN.F.EBA 518a; MNHN.F.EBA 518b; MNHN.F.EBA 520; MNHN.F.EBA 521; MNHN.F.EBA 522; MNHN.F.EBA 523; MNHN.F.EBA 524; MNHN.F.EBA 525; MNHN.F.EBA 526; MNHN.F.EBA 527; MNHN.F.EBA 528.

Repository information: Muséum national d'Histoire Naturelle, Collection of fossils, Collection of fossils from the Eocene of Aquitaine Basin (EBA); 57 rue Cuvier, CP38, F-75005, Paris, France.

No permits were required for the described study, which complied with all relevant regulations.

Nomenclatural Acts

The electronic edition of this article conforms to the requirements of the amended International Code of Zoological Nomenclature, and hence the new names contained herein are available under that Code from the electronic edition of this article. This published work and the nomenclatural acts it contains have been registered in ZooBank, the online registration system for the ICZN. The ZooBank LSIDs (Life Science Identifiers) can be resolved and the associated information viewed through any standard web browser by appending the LSID to the prefix "http://zoobank.org/". The LSID for this publication is: urn:lsid:zoobank.org:pub:8C2E4D2E-4890-4625-ABA9-B3137A6DB276. The electronic edition of this work was published in a journal with an ISSN, and has been archived and is available from the following digital repositories: PubMed Central, LOCKSS.

Terminology

We follow the dental terminology of Van Valen [32] for the molars, and of Ginsburg [33] for the premolars. The dental measurements (Length x Width in mm.) follow Gingerich and Deutsch [34]. The statistical parameters are the observed range (OR) and mean (M).

The osteological terminology follows that of Miller et al. [35]; while the measurements of the postcranial elements follow Argot [36].

Phylogenetic analysis

The taxa that have been included are listed in S1 Text. The data matrix is based on Holroyd [37] with new character definitions. It consists of 49 dental characters (32 binary characters and 17 multistate characters) and one character related to body mass (S2 Text). It includes 18 hyainailourine taxa.

The polarization of the characters was based on outgroup comparison criteria. All the multistate characters were treated as unordered–Holroyd [37] treated characters 34 and 44 as ordered.

We used the basal hyaenodonts *Tinerhodon*, *Eoproviverra eisenmanni*, and *Prototomus* as outgroups to Hyainailouridae. Because the dentition of *E. eisenmanni* is not entirely known (only several molars have been discovered), we used its contemporaneous *Parvagula* in order to code the premolars and the proviverrine *Proviverra typica* for coding the remaining characters. *P. typica* is considered one of the most primitive hyaenodonts–and thus proviverrines–despite its Eocene age [38]. *Prototomus* was coded based on the two European species: *P. minimus* and *P. girardoti* and the oldest North American species: *P. deimos* and *P. phobos*.

The data matrix (S1 File) was assembled with WinClada [39] and the parsimony analyses were performed with TNT [40] using implicit enumeration. The consensus trees have been assembled and analyzed with WinClada. We performed three analyses: the first one includes

all the taxa and characters, while *Leakitherium* is excluded in the second and third analyses because it is very poorly known.

The genus *Leakitherium* is only known from two maxillary fragments that display P^4, M^1 and M^2 [3]. Morales et al. [41] referred a fragment of an M_2 to the genus. The taxonomic status of *Leakitherium* among hyainailourines is unresolved [41]. They hypothesized that *Leakitherium* is a synonym for *Isohyaenodon andrewsi*. In this analysis *Leakitherium* is closely related to Miocene hyainailourines such as *Megistotherium* and *Isohyaenodon*, but the hypothesis of Morales et al. [41] is not supported here.

Finally, the first character, which concerns body mass (S2 Text), is excluded in the third analysis because it could correspond to ecologic convergence rather than reflecting a phylogenetic signal.

After each analysis, each node of the strict consensus tree was assigned a Bremer support calculated with TNT for ten supplementary steps.

Results

Systematic Paleontology

Placentalia Owen, 1837 [42]

Ferae Linnaeus, 1758 [43]

Hyaenodonta Van Valen, 1967 [44]

Diagnosis (emended after Gunnell [45]). Elongate, narrow skull with narrow basicranium and high, narrow occiput; transversally constricted interorbital region; tritubercular to sectorial molars with carnassial blades in P^4, M^1, M^2, and M_1, M_2 and M_3 (except in Limnocyoninae and Machaeroidinae which have lost the posterior-most molars); M^3 present in most taxa; M_3 always present (except in Limnocyoninae and Machaeroidinae); manus and pes mesaxonic, plantigrade to digitigrade posture; fibula articulated with calcaneum; astragalar-cuboid articulation reduced or absent; terminal phalanges compressed and fissured at tip in most taxa; central, scaphoid, and lunar not fused (except perhaps in the hyainailourines *Hyainailouros* and *Pterodon*).

Distribution. Africa, Asia, Europe, and North America; Selandian (Paleocene) to Serravallian (Miocene).

Included families. Hyaenodontidae Leidy, 1869 [46], emended in this paper; Hyainailouridae Pilgrim, 1932 [47], emended in this paper;? Koholiinae Crochet, 1988 [48].

Notes. As in other specialized carnivorous mammals, hyaenodonts possess secant teeth. These meat-slicing teeth, also called carnassial teeth, are located in M^{1-2} and M_{2-3} position in Hyaenodonta [45]. Hyaenodonta was traditionally placed in the extinct order Creodonta together with the Oxyaenidae. Some workers have suggested a diphyletic origin for Creodonta [1,38,49–51]. While the separate origins of Oxyaenidae and Hyaenodontidae have not been established in a cladistic framework, Solé [51] proposed to raise these two families to the ordinal level–Oxyaenodonta and Hyaenodonta, and not Hyaenodontida as mistakenly used by several authors [6,51–53]–to highlight the possible separate evolutionary origins of these two extinct carnivorous mammalian lineages. It is worth mentioning that Spaulding et al. [54] recovered a monophyletic Creodonta, but their phylogenetic analyses, which addressed the relationships of Cetacea among mammals, only included four creodonts (three hyaenodonts and only one oxyaenid).

The systematic position of Koholiinae among Hyaenodonta is presently uncertain because cranial material is unknown (see below).

Discussion of the relationships within Hyaenodonta. Polly [38] established that hypercarnivory (characterized by reduction of the metaconids, simplification of the talonid,

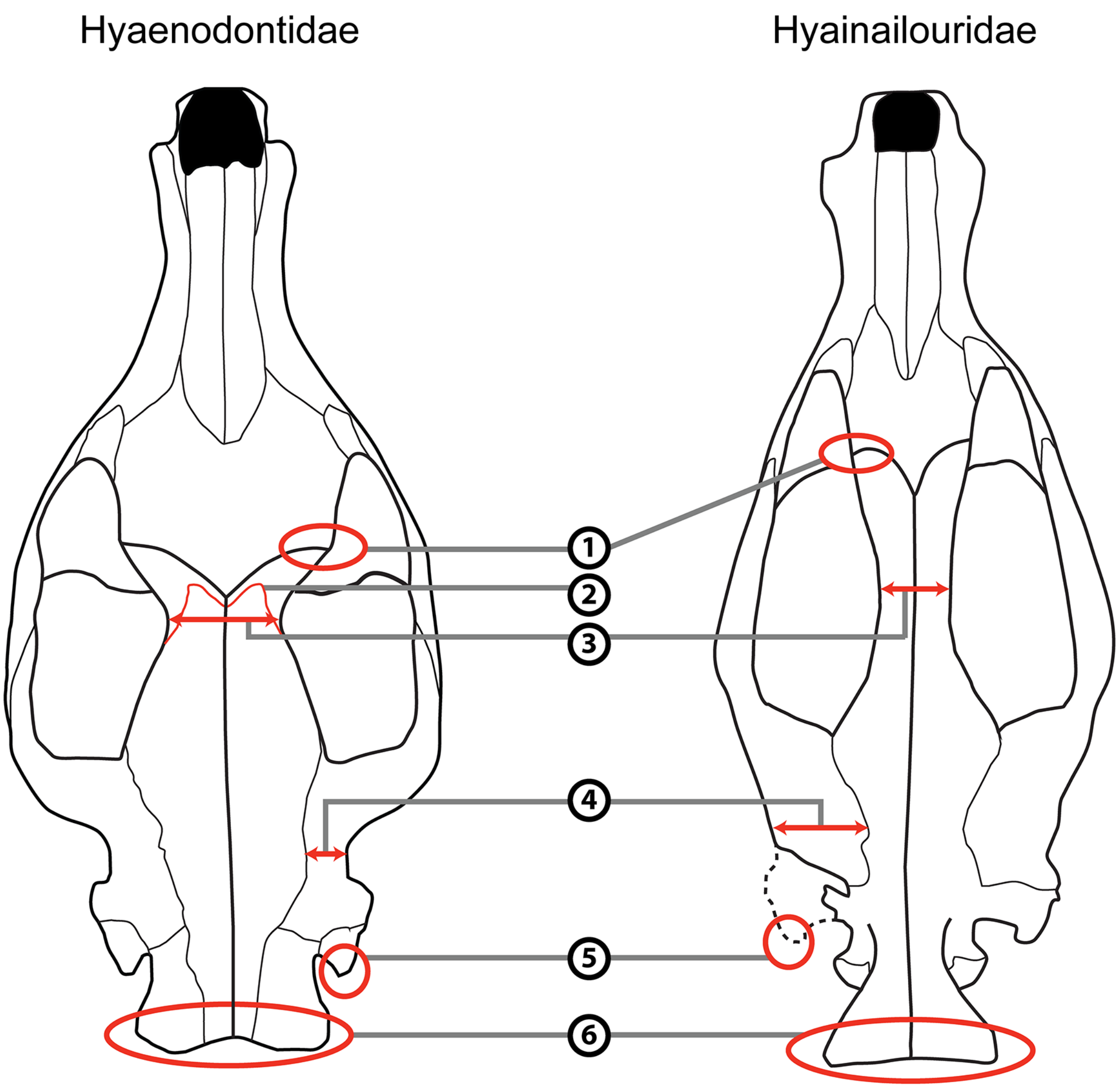

Fig 3. Comparison of the skull in dorsal view of Hyaenodontidae (left) and Hyainailouridae (right). See Table 1 for description of the numbered features. Left: skull of *Sinopa grangeri* redrawn after Matthew ([55], Fig 4); right: skull of *Apterodon macrognathus* redrawn after Osborn ([56]; Fig 1A).

doi:10.1371/journal.pone.0135698.g003

extension of the metastyle, reduction of the protocone, among other features) arose at least twice within hyaenodonts: among Hyainailourinae (Pterodontinae *sensu* Polly) and Hyaenodontinae. The distinction between the two subfamilies includes several cranial features. Polly noted that hyainailourines are characterized by a nuchal crest that does not extend laterally toward the mastoid processes and a circular subarcuate fossa on the petrosal. Hyaenodontines are characterized by a nuchal crest that extends toward the mastoid process, a robust bridge over the foramen stylomastoid primitivum, the absence of a bony ridge dividing the posterior petrosal sinus from the foramen stylomastoid primitivum, the presence of an inflated posterior petrosal sinus, and the presence of a horse-shoe shaped subarcuate fossa.

The cranial morphology of the hyainailourines is similar to that of the European and African hyaenodont subfamily Apterodontinae, notably in the short lateral extension of the nuchal crest (Fig 3, Feature 6; Table 1). Moreover, based on the present study, we can add the following features that seem to unite Hyainailouridae (Hyainailourinae+Apterodontinae): presence

Table 1. Comparison of the cranial features of Hyaenodontidae and Hyainailouridae.

Feature	Hyainailouridae	Hyaenodontidae
**1: Postorbital process**	Poorly developed; except in Miocene *Hemipsalodon*	Well developed
**2: Suture frontal parietal**	Early obliteration of the suture (except in *Hemipsalodon*)	Present
**3: Constriction before the braincase**	Absent	Present
**4: Squamosal**	Laterally expanded posterior to the zygomatic arch	Poorly laterally expanded
**5: Expansion of the mastoido-paroccipital apophysis**	Weak posterior extension in later forms	Always posteriorly extended
**6: Expansion of the nuchal crest to mastoid process**	Nuchal crest does not reach the mastoid process	Nuchal crest reaches the mastoid process
**7: Preglenoid crest**	Present	Absent
**8: Lateral expansion of the mastoid process**	Mastoid process laterally expanded in later forms	Mastoid process poorly laterally expanded in later forms
**9: Occipital condyle**	Large	Small
**10: Process at the junction between the maxillary and jugal**	Rarely present	Often present
**11: Suture between the jugal and squamosal**	Anteroposteriorly extended	Poorly anteroposteriorly extended
**12: Pterygoid**	Extended anteroposteriorly and ventrally	Poorly extended anteroposteriorly and ventrally
**13: Mental foramina**	Often more than two	Very rarely more than two
**14: Angular process**	Poorly developed; absence of anterior concavity	Robust with a anteriorly located concavity

doi:10.1371/journal.pone.0135698.t001

of a preglenoid crest (Figs 4 and 5, Feature 7; Table 1), early obliteration of the suture between the frontal and parietal bones (Figs 3 and 5, Feature 2; Table 1), anteroposteriorly extended suture between the jugal and squamosal (Fig 5, Feature 11; Table 1), a lateral expansion of the squamosal posterior to the zygomatic arch (Fig 3, Feature 4; Table 1), and large occipital condyles (Figs 4 and 5, Feature 9; Table 1). We can also note that apterodontines and hyainailourines often display more than two mental foramina (Fig 5, Feature 13; Table 1), while hyaenodontids very rarely display more than two, and have a weak ventral concavity anterior to the angular process (Fig 5, Feature 14; Table 1). The younger representatives of the clade (e.g., *Hyainailouros*) are characterized by a transverse elongation of the mastoid process (Fig 4, Feature 8; Table 1), rather than an anteroposterior elongation (Figs 3 and 4, Feature 5; Table 1) as observed in the earliest species in the order Hyaenodonta. Finally, the narrowest part of the braincase is immediately behind the postorbital processes in Hyaenodontidae, while this constriction is more posterior in Hyainailouridae (at least at the middle of the parietals) (Fig 3, Feature 3; Table 1).

Some representatives of hyainailourines have lost the P_1 [9]. This feature is one of the main characteristics of Koholiinae [1]. Koholiines and hyainailourines share the latter feature, as well as the presence of pronounced secant teeth and extensive radiations in Africa. However, the koholiines differ from hyainailourines in featuring a robust angular process that is preceded anteriorly by a concavity (Fig 5, Feature 14; Table 1). It is thus presently difficult to refer the koholiines to either Hyainailouridae or Hyaenodontidae.

The cranial morphology of Hyaenodontidae is on the whole similar to Hyaenodontinae in Polly [38]. The clade is characterized by the morphology of the nuchal crest, which extends toward the mastoid process (Figs 3 and 5, Feature 6; Table 1). We can add several features not described previously to distinguish Hyaenodontidae from Hyainailouridae. Hyaenodontids generally possess: a well-developed postorbital process (Figs 3 and 5, Feature 1; Table 1), a short extension of the jugal/squamosal suture anteroposteriorly (Fig 5, Feature 11; Table 1),

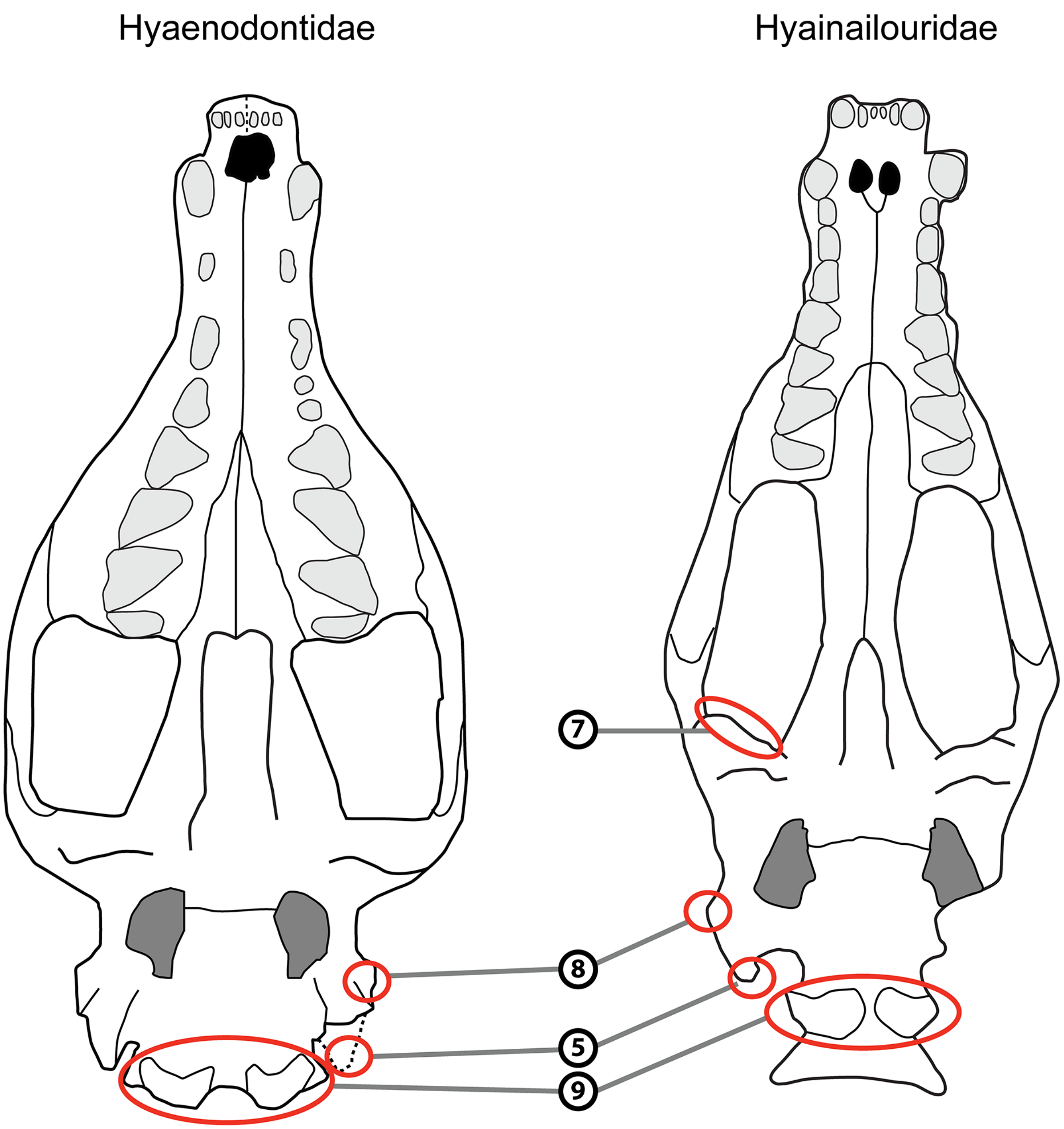

Fig 4. Comparison of the skull in ventral view of Hyaenodontidae (left) and Hyainailouridae (right). See Table 1 for description of the numbered features. Left: skull of *Sinopa grangeri* redrawn after Matthew ([55], Fig 5); right: skull of *Apterodon macrognathus* redrawn after Szalay ([57]; Fig 7).

doi:10.1371/journal.pone.0135698.g004

absence of a preglenoid crest (Figs 4 and 5, feature 7; Table 1), a process at the junction between the maxillary and the jugal (Fig 5, Feature 10; Table 1), and a small occipital condyle (Figs 4 and 5, Feature 9; Table 1). Unlike Hyainailouridae, Hyaenodontidae exhibit a clear suture between the parietal and frontal (Figs 3 and 5, Feature 2; Table 1) and the suture is located close to the anterior constriction of the braincase (Fig 3, Feature 3; Table 1). Hyaenodontids all possess a single- or double-rooted p1 and very rarely display more than two mental foramina (Fig 5, Feature 13; Table 1). Hyaenodontid pterygoids are generally less developed ventrally and posteriorly than hyainailourid pterygoids (Fig 5, Feature 12; Table 1). In almost all

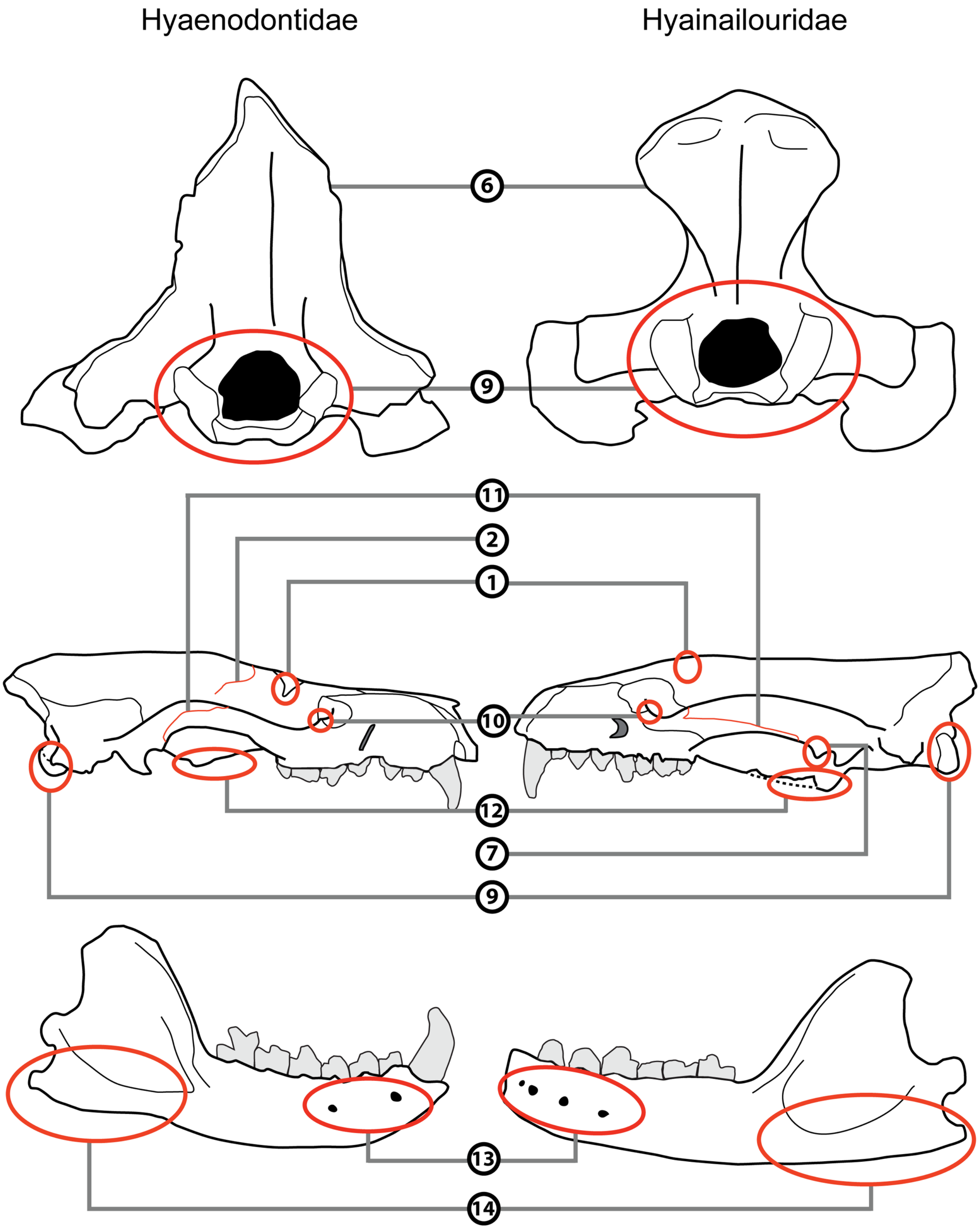

Hyaenodontidae
Hyainailouridae
6
9
11
2
1
10
12
7
9
13
14

Fig 5. Comparison of the skull in posterior (upper) and lateral (middle) views and of the mandible in labial view (lower) of Hyaenodontidae (left) and Hyainailouridae (right). See Table 1 for description of the numbered features. Upper: skulls of *Pterodon dasyuroides* (right) and *Hyaenodon compressus* (left) redrawn after Polly ([38]; Fig 11A and 11B); middle: skulls of *Limnocyon verus* (left) and *Apterodon macrognathus* (right) redrawn respectively after Matthew ([58]; Fig 53) and Szalay ([57], Fig 6); lower: mandibles of *Limnocyon verus* (left) and *Apterodon macrognathus* (right) redrawn respectively after Matthew ([58]; Fig 53) and Szalay ([57], Fig 9).

doi:10.1371/journal.pone.0135698.g005

hyaenodontids the mandible possesses a concavity on the ventral margin anterior to the angular process and the latter is generally robust and projects more ventrally in Hyaenodontidae (Fig 5, Feature 14; Table 1).

We tentatively refer the Teratodontinae as recently defined by Solé et al. [6] to Hyaenodontidae based on the skull of *Dissopsalis* described by Colbert [59] and mandibles available for the teratodontine genera. The fragmentary skull of *Dissopsalis* seems to display developed postorbital process (Figs 3 and 5, Feature 1; Table 1), constriction before the braincase (Fig 3, Feature 3; Table 1), and a distally elongated mastoido-paroccipital apophysis (Figs 3 and 4, Feature 5; Table 1). Colbert [59] noted that *Dissopsalis* possesses a developed preglenoid crest (Figs 4 and 5, Feature 7; Table 1), a feature of Hyainailouridae; however, based on his drawings, we assess that the morphology of the mandibular fossa is similar to that of Hyaenodontidae rather than to that of Hyainailouridae. Finally, teratodontines display a concavity anterior to a robust angular process (Fig 5, Feature 14; Table 1) as in other Hyaenodontidae.

The Indohyaenodontidae seem to be endemic in South (India, Pakistan) and Southeast (Myanmar) of Asia [6]. Unfortunately, only few cranial remains are known for this subfamily [60–61]. Cranial and dental specimens show typical features of Hyaenodontidae such as the presence of a concavity anterior to the angular process (Fig 5, Feature 14; Table 1), the presence of only two mental foramina (Fig 5, Feature 13; Table 1), the absence of a preglenoid crest (Figs 4 and 5, Feature 7; Table 1), a process at the junction between the maxillary and the jugal (Fig 5, Feature 10; Table 1), a well-developed postorbital process (Figs 3 and 5, Feature 1; Table 1), and a clear suture between the parietal and frontal (Figs 3 and 5, Feature 2; Table 1). We therefore include this subfamily in Hyaenodontidae.

Machaeroidinae has been often considered to be closely related to Limnocyoninae [62]. Machaeroidinae and Limnocyoninae notably exhibit an extension of the nuchal crest towards the mastoid, as seen in other species in Hyaenodontidae. Species in both subfamilies have moreover lost M_3 and M^3 (except in their oldest representative *Prolimnocyon*), supporting a close relationship between these families and the inclusion of both Machaeroidinae and Limnocyoninae in Hyaenodonta. Several authors (e.g. Muizon & Lange-Badré [63]) however referred the Machaeroidinae to Oxyaenodonta; Zack [64] recently proposed arguments based on postcranial elements to support this hypothesis.

The two cranial morphotypes described and discussed here may be useful for clarifying the relationships within Hyaenodonta, though these features await rigorous evaluation in a cladistic analysis to establish plesiomorphic and apomorphic states of characters. Because the two morphotypes imply distinct recruitment of the cranial muscles and both morphotypes seem to be a combination of primitive and derived features, we hypothesize that these two cranial morphotypes appeared early in the radiation of Hyaenodonta. Based on the presence of hyaenodontids in the Paleocene of Asia, we place the radiation of Hyaenodonta and divergence of Hyaenodontidae and Hyainailouridae during the Selandian, if not earlier [2].

Whether or not the order Hyaenodonta is rooted in Africa (Hyainailourindae + Hyaenodontidae) as proposed by Gheerbrant [5], Gheerbrant et al. [50] and Solé et al. [1,6] needs further clarification.

One can note that Hyaenodontidae mainly radiated in Laurasia (Fig 6) where six clades are known (Hyaenodontinae, Limnocyoninae, Sinopinae, Arfiinae, Proviverrinae, and probably

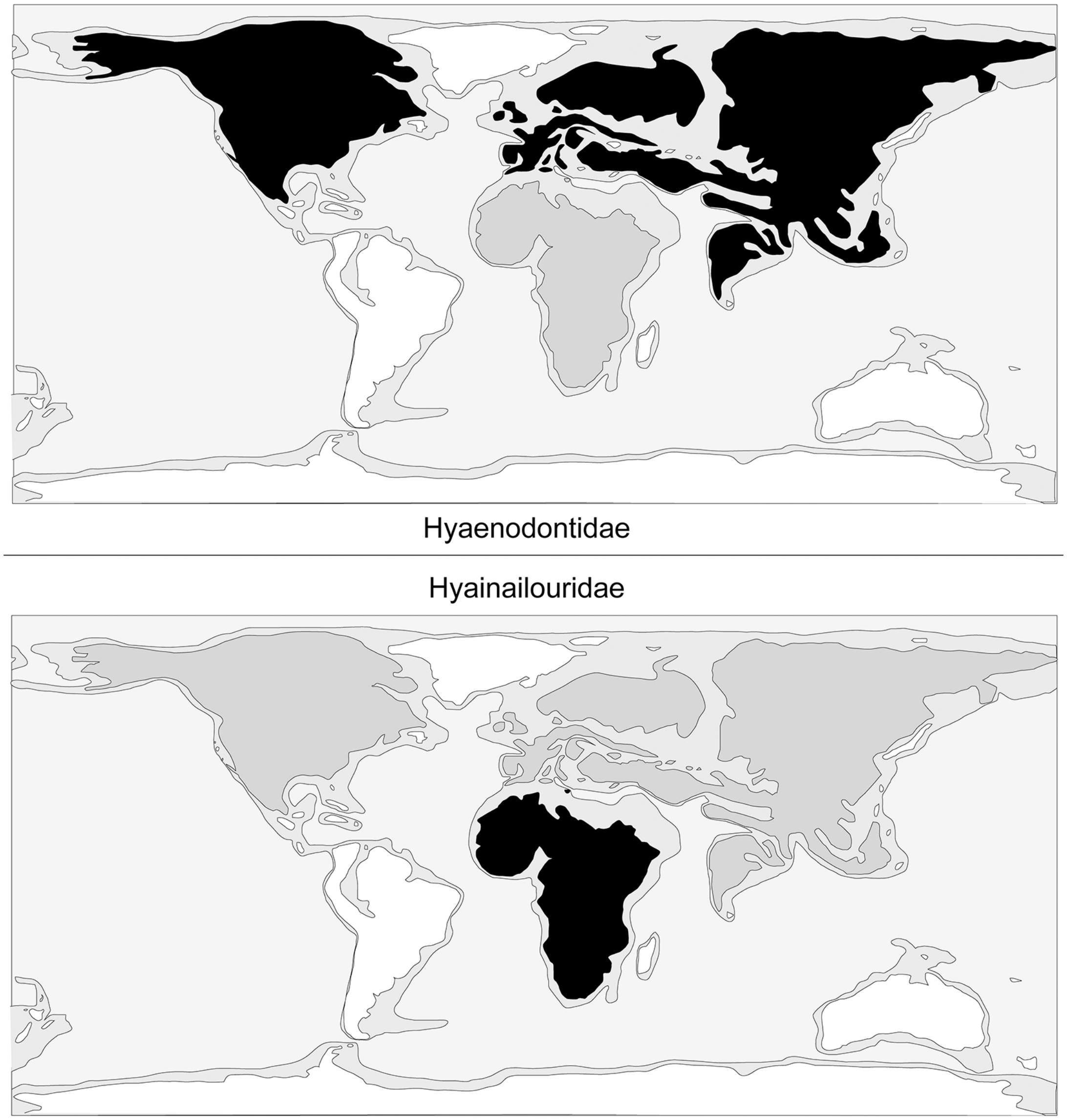

Fig 6. Comparison of the geographic distribution of Hyaenodontidae (top) and Hyainailouridae (bottom). White: no fossils recorded; Grey: limited diversity; Black: main radiation.

doi:10.1371/journal.pone.0135698.g006

Machaeroidinae), while Hyainailouridae principally radiated in Africa (Fig 6), where koholiines, apterodontines and hyainailourines find their earliest record [1,6,52].

Concerning the dispersals of these two families, it is worth mentioning that apterodontines dispersed into Europe and hyainailourines dispersed into North America, Europe and Asia [9,65–66]. Among Hyaenodontidae, only Teratodontinae and Indohyaenodontinae are known exclusively from Gondwanan landmasses (Africa and South/South-East of Asia). If Hyaenodontidae appeared in Laurasia, these two subfamilies may have dispersed into Africa during the earliest Eocene.

Hyaenodontidae Leidy, 1869 emend. [46]

Diagnosis. The members of this family share a cranial pattern characterized by the presence of a visible suture between the parietal and frontal and a well-developed postorbital

process, a constriction anterior to the braincase, pterygoid generally shorter than in Hyainailouridae, a short extension of the jugal/squamosal suture anteroposteriorly, the absence of preglenoid crest, a distally elongated mastoido-paroccipital apophysis, a nuchal crest extending to the mastoid process, small occipital condyles, the presence of only two mental foramina, and a concavity anterior to the angular process (less pronounced in hyaenodontines). The P_1 (single- or double-rooted) is usually present.

Distribution. Africa, Asia, Europe, and North America; Thanetian (Paleocene) to Serravallian (Miocene).

Included subfamilies. Arfiinae Solé, 2013 [51]; Hyaenodontinae Leidy, 1869 [46]; Indohyaenodontinae Solé et al., 2013 [6]; Limnocyoninae Wortman, 1902 [67];? Machaeroidinae Matthew, 1909 [58]; Proviverrinae Schlosser, 1886 [68]; Sinopinae Solé, 2013 [51]; Teratodontinae Savage, 1965 [3].

Note. The systematic position of the saber-toothed Machaeroidinae among Hyaenodonta or Oxyaenodonta is presently uncertain (see Gunnell [45]).

Hyainailouridae Pilgrim, 1832 emend. [47]

Diagnosis. The members of this family share a cranial pattern characterized by a massive skull, the absence of visible suture between the parietal and frontal, a weak postorbital process, a pterygoid generally extended ventrally and distally, an anteroposteriorly extended suture of the jugal/squamosal, the presence of a preglenoid crest, a lateral expansion of the squamosal posterior to the zygomatic arch, a transversally expanded mastoid process (in later forms), a nuchal crest that does not extend laterally to mastoid process, large occipital condyles, and the presence two or more mental foramina. The P_1, if present, is small (single-rooted).

Distribution. Africa, Asia, and Europe; late Ypresian or early Lutetian (Paleocene) to Serravallian (Miocene).

Included subfamilies. Apterodontinae Szalay, 1967 [57]; Hyainailourinae Pilgrim, 1932 [47].

Relationships between the Hyainailourinae and Apterodontinae. Hyainailourinae and Apterodontinae are diverse during the Paleogene in Africa [3,52,69–70]. The two groups are also recorded in Europe: the Hyainailourinae are recorded in the Late Eocene and Early to Middle Miocene, while the Apterodotinae are known in the Early Oligocene [9,33,66,71]. Hyainailourinae is also known in Early to Middle Miocene of Asia [47].

Recent discoveries in North Africa place the youngest possible origin of Hyainailourinae in the late Early or early Middle Eocene [6] and the youngest possible origin of Apterodontinae in the late Middle Eocene [52], implying an Afro-Arabian origin for these subfamilies. The presence of Hyainailourinae and Apterodontinae in Eurasia is likely a consequence of Northern immigrations (see below).

Apterodontinae is a peculiar group among Hyaenodonta [37,38,52,57]. Apterodontines are characterized by the loss of the metaconid, the reduction of the paraconid, and retention of a prominent talonid on the lower molars where most hyaenodonts with reduced metaconids have also reduced the talonid and increased the relative size of the paraconid. Based on postcranial material, Grohé et al. [52] proposed semi-aquatic habits for *Apterodon*. They suggested that the unusual dentition of *Apterodon* may have been related to a semi-aquatic lifestyle with simplified and tall teeth, employed for catching aquatic prey such as 'fish' and/or 'invertebrates' [52].

In contrast to apterodontines, large hyainailourines were likely hunters and scavengers and were probably ecologically analogous to the extant hyenas and the extinct borophagines; the latter however differ from large hyainailourines in exhibiting cursorial adaptations (see [72] and [73]).

The possibility of a close relationship between apterodontines and hyainailourines based on dental and cranial characters has been suggested before [6,38,52,61]. Here we explicitly identify

numerous cranial features (e.g., absence of visible suture between the parietal and frontal, weak postorbital process, presence of a preglenoid crest, large occipital condyles) that group Apterodontinae and Hyainailourinae within the family Hyainailouridae (see above). The strongest similarities between apterodontines and hyainailourines lie in the posterior region of the skull related to the characteristic rearrangement of cervical musculature first described by Polly [38] in Hyainailourinae (Pterodontinae in Polly [38]).

The skull of *Apterodon* is anteroposteriorly elongate. The oldest hyainailourines such as *Pterodon* and *Kerberos* also exhibit anteroposteriorly elongate skulls, while others, such as the later hyainailourines *Paroxyaena*, *Hemipsalodon*, and *Hyainailouros*, differ from *Apterodon* and early hyainailoruines in featuring anteroposteriorly short rostral and basicranial regions. This suggests that *Kerberos* and *Apterodon* possess a primitive overall cranial morphology for Hyainailouridae.

Apterodon differs from hyainailourines in several features, which include a more robust zygomatic arch. This feature provides greater surface area for the origin of larger masseter muscles in apterodontines. On the mandible, the masseteric fossa is shallow but anteroposteriorly elongate, providing a long insertion for the masseter relative to the masseteric fossa in *Kerberos* and *Pterodon*, which further supports the important role of the masseter muscles in apterodontines.

Apterodon also differs from hyainailourines in the arrangement of the upper tooth rows that are close to parallel along the palate while nearly all other hyaenodonts have tooth rows that are distinctively diverging posteriorly. This parallel alignment may be related to semi-aquatic feeding as well. The facial morphology of apterodontines also differs from hyainailourines that have a strong narrowing of the facial morphology posterior to the canines [8,9]. Besides the differences in the masseteric fossa morphology, the mandibles of apterodontines and hyainailourines are generally similar. It is interesting to note that *Apterodon macrognathus* and *Kerberos* share the presence of a dorsally inflected, thin and pointed angular process, and an extended coronoid crest.

Concerning the postcranium, the only elements that can be compared between Apterontinae and Hyainailourinae are the distal part of the fibula, the astragalus, and the calcaneum. The distal part of the fibula is very similar in these two groups. However, there are differences in the tarsal bones. The tuber calcaneus of *Kerberos* and *Hyainailouros* is shorter and wider than that of *Apterodon* and the ectal facet of the hyainailourines, though poorly preserved on the specimen referred to *Kerberos*, seems to be shorter than the ectal facet of *Apterodon*. The neck of the astragalus of *Kerberos* and *Hyainailouros* is also shorter and narrower than that of *Apterodon*, though this difference in morphology may be related to the larger body size of the hyainailourine species. Apterodoninae and Hyainailourinae share the shallow groove of the tibial facet. This feature suggests that both groups maintained a plantigrade foot posture, though this morphology seems to be primitive in Hyaenodonta. The differences suggest a more weight-bearing mode of life in *Kerberos*, while the adaptations of *Apterodon* may suggest adaptations towards semi-aquatic mode of life.

In conclusion, apterodontines and primitive hyainailourines share a characteristic cranial construction. Broad attachments for nuchal musculature could have been adaptive for scavenging/hunting in terrestrial and aquatic environments, but this hypothesis needs to be tested in the future. However, numerous differences are present in the dentition and postcranium, and we advocate retaining separate subfamily designations for these lineages.

Hyainailourinae Pilgrim, 1932 [47]

Emended diagnosis (emended after Holroyd [69]). Hyainailouridae with high and secant paraconid, connate metacone and paracone on M^1-M^2, weak to absent P^3 lingual cingulum, P^4

lacking continuous lingual cingulum, relatively large anterior keels on lower molars, M_3 talonid reduced relative to that of M_1–M_2, lower molar protoconids and paraconids subequal in length, facial region constricted lateromedially at P^2 and abruptly expanded caudal to P^4, infraorbital process poorly developed, massive zygomatic arch, anterior margin of choanae located at the level of M^3, and circular subarcuate fossa present on petrosal.

Note. Hyainailourinae (*sensu* Solé et al., 2013 [6]) includes 12 genera: *Akhnatenavus* Holroyd, 1999 [69], *Furodon* Solé et al., 2013 [6], *Hemipsalodon* Cope, 1885 [74], *Hyainailouros* Biedermann, 1863 [75], *Isohyaenodon* Savage, 1965 [3], *Kerberos* gen. nov. (present work), *Leakitherium* Savage, 1965 [3], *Parapterodon* Lange-Badré, 1979 [9], *Paroxyaena* Martin, 1906 [76], *Parvavorodon* Solé et al., 2013 [6], *Pterodon* Blainville, 1839 [77] and *Sivapterodon* Ginsburg, 1980 [4].

However, agreement on which species should be assigned to different hyainailourine genera is not firmly established. We follow Morlo et al. [78] by assigning only two species to *Hyainailouros*: *Hyainailouros bugtiensis* Pilgrim, 1912 [79] (= *Megistotherium osteothlastes* Savage, 1973 [80]) and *Hyainailouros sulzeri* Biedermann, 1863 [75] (= *Hyainailouros fourtaui* Koenigswald, 1947 [81] = *Hyainailouros napakensis* Ginsburg, 1980 [4] = *Hyainailouros nyanzae* Savage, 1965 [3]). Concerning *Isohyaenodon*, we partially follow Morales et al. [41], by including three species: *Isohyaenodon andrewsi* Savage, 1965 [3], *Isohyaenodon zadoki* Savage, 1965 [3] (= *Isohyaenodon matthewi* Savage, 1965 [3]) and *Isohyaenodon pilgrimi* Savage, 1965 [3]; however *Leakitherium* is not synonymized with *Isohyaenodon andrewsi* herein. Moreover, our analysis shows that '*Pterodon*' might not be a natural group but represents a polyphyletic genus.

Paroxyaenini Lavrov, 2007 [8]

Diagnosis (emended after Lavrov [8]). Medium-sized Hyainailourinae with short, wide facial region. Dental formula: I3/3, C1/1, P4/4, M3/3. P^1 single-rooted, P^2 and P^3 double-rooted. Protocone well developed on P^3. Amphicones of M^1 and M^2 incompletely fused; apices of paracone and metacone distinctly separate; cingulum well developed on molars and premolars; M^1, M^2, and DP^4 showing well-developed precingula with many cuspules. Metastyle of M^1 and M^2 approximately half of the tooth length. Postprotocrista absent from M^1 and M^2; QM1 approximately 120°–130°, QM2 approximately 125°; surface of enamel of P^3 –M^2 rugose. Fissura orbitalis and foramen rotundum fused into one foramen. Medial part of glenoid fossa of jaw joint very deep. Large maxillo-nasalis fossa located dorsal to alveoli of P^4–M^2. Tentorium well developed.

Included genera. *Hemipsalodon* Cope, 1885 [74] and *Paroxyaena* Martin, 1906 [76].

Distribution. Europe and North America; Bartonian (Eocene) to Priabonian (Eocene).

Hyainailourini Ginsburg, 1980 [4]

Diagnosis. Small to large-sized Hyainailourines: facial region elongated in oldest representatives (e.g., *Kerberos*), but shorter in youngest ones (e.g., *Hyainailouros*). Dental formula: I1-3/2-3, C1/1, P4/3-4, M2-3/3. P^1 single (e.g., *Hyainailouros*) or double-rooted (e.g., *Kerberos*), Amphicones of M^1 and M^2 nearly to completely fused; protocone on molars very reduced; cingula weakly developed on molars and premolars. Fissura orbitalis and foramen rotundum separated.

Included genera. *Akhnatenavus* Holroyd, 1999 [69], *Hyainailouros* Biedermann, 1863 [75], *Isohyaenodon* Savage, 1965 [3], *Kerberos* gen. nov., *Leakitherium* Savage, 1965 [3], *Parapterodon* Lange-Badré, 1979 [9], *Pterodon* Blainville, 1839 [77] and *Sivapterodon* Ginsburg, 1980 [4].

Distribution. Africa and Europe; Bartonian (Eocene) to Serravallian (Miocene).

Kerberos gen. nov.

urn:lsid:zoobank.org:act:CF40CC5A-025F-464B-8769-54471DD888B0

Diagnosis. Same as for the type and only species.

Type and only species. *Kerberos langebadreae* nov. sp.

Type locality. Montespieu (locality close to Lautrec), Tarn, France; MP16, Bartonian, Eocene.

Type horizon and age. Formation des Molasses de Saix et de Lautrec, Grès de Puech Auriol et de Venès, Bartonian, Eocene.

Etymology. Cerberus (*Kerberos* in Greek) is an impressive mythological multi-headed dog–Hellhound–that guards the entrance of the Underworld.

Kerberos langebadreae sp. nov.

urn:lsid:zoobank.org:act:8645FC88-4DAF-4084-9E14-64AE1D707EC7 (Figs 7–12)

Diagnosis. This large-sized hyainailourine displays a particular combination of primitive features, namely an anteriorly narrow mandible and a small diastema between P_1 and P_2, and derived features, namely a reduced parastylar area on upper molars, a large protocone on P^3, and premaxilla narrow throughout its whole length. It differs from *Paroxyaena* by having a more closely appressed paracone and metacone, and more reduced protocone on upper molars, and a shorter and narrower talonid on the lower molars. *Kerberos* differs from *Pterodon dasyuroides* by possessing larger P_1 and P^1, and from *Parapterodon* by retaining the P^1, exhibiting a more reduced parastylar area, and a more reduced protocone on upper molars.

Etymology. Dedicated to Dr. Lange-Badré, who greatly improved our knowledge of Eocene carnivorous mammals.

Type locality. Montespieu (locality close to Lautrec), Tarn, France; MP16, Bartonian, Eocene.

Type horizon and age. Formation des Molasses de Saix et de Lautrec, Grès de Puech Auriol et de Venès, Bartonian, Eocene.

Holotype. MNHN.F.EBA 517, nearly complete skull bearing on right side I^2-I^3 and P^2-M^3, and on left side I^2-I^3, P^1, P^4-M^3.

Referred material. MNHN.F.EBA 518a, left hemimandible bearing C and M_2-M_3; MNHN.F.EBA 518b, right hemimandible bearing P_2-P_4 and M_2-M_3; MNHN.F.EBA 520, right fibula; MNHN.F.EBA 521, right astragalus; MNHN.F.EBA 522, right calcaneus; MNHN.F.EBA 523, right metatarsal I; MNHN.F.EBA 524, right metatarsal II; MNHN.F.EBA 525, right metatarsal III; MNHN.F.EBA 526, left metatarsal II; MNHN.F.EBA 527, right middle phalanx; MNHN.F.EBA 528, right middle phalanx.

Measurements. Tables 2 and 3.

Description of the skull, mandible and dentition. The skull of the holotype is distorted transversally but is almost complete (Fig 7). It is also riddled with postmortem cracks and breaks, which partly obliterate the sutures (see Fig 13 for drawings of the skull with indications of the sutures). The auditory region, which is poorly preserved, will not be described thoroughly. The taxa chosen for comparison assessed from original specimens housed in the MNHN and literature represent the different subfamilies of Hyaenodonta (i.e., the Proviverrinae, Hyainailourinae, Hyaenodontinae, and Limnocyoninae), which are known from the Bartonian, Priabonian and Oligocene.

The most striking feature of the skull is its tremendous size compared to contemporaneous European proviverrines. The condylobasal length (~ 35 cm) approaches that of a female *Ursus arctos* [82]. The rostrum is very short, even shorter than that of the brevirostrate *Hyaenodon brachyrhynchus*. The stoutness of the rostrum contrasts with the relatively long ethmoid

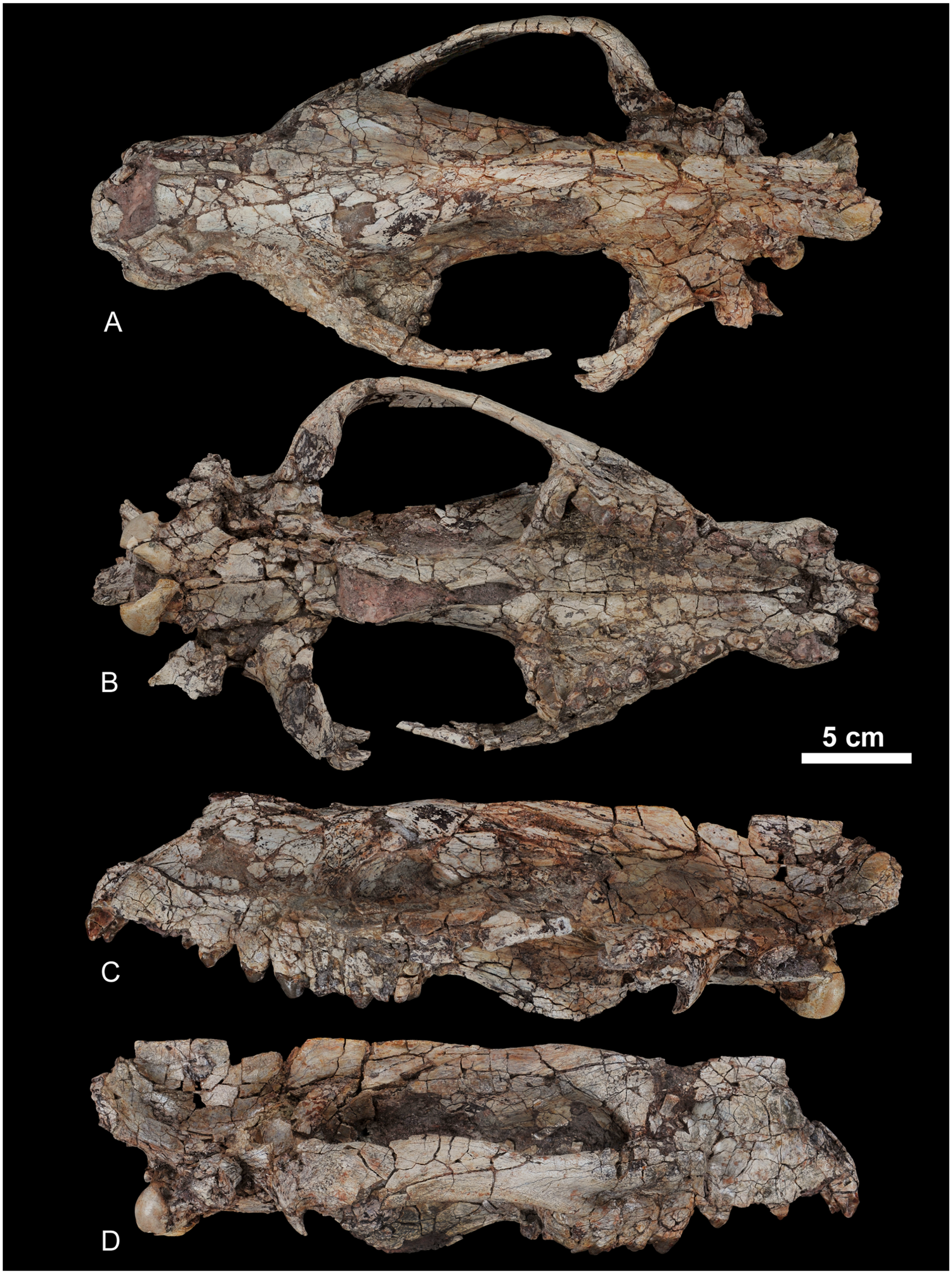
A
B
5 cm
C
D

Fig 7. Skull of *Kerberos langebadreae* gen. & sp. nov (holotype, MNHN.F.EBA 517). A, dorsal view; B, ventral view; C, left lateral view; D, right lateral view.

doi:10.1371/journal.pone.0135698.g007

region. Even though the basicranium is crushed transversally, it seems to have been mediolaterally extensive as in *Pterodon dasyuroides*.

Premaxilla (Fig 13, Premax.). In lateral view (Fig 7), the opening of the nasal cavity forms an angle of approximately 45° with the anteroposterior lengthening of the skull, which approaches the condition of *H. brachyrhynchus*. The nasal aperture forms a more opened angle in the proviverrine *Cynohyaenodon cayluxi* and a slightly sharper angle in *P. dasyuroides*. The premaxilla differs from that of other hyaenodonts as the ascending ramus is narrow along its entire length. The posterior portion of the premaxilla is not extensive, reaching just posterior to the dorsal edge of the opening of the nasal cavity. The general hyaenodont condition is an extended premaxilla that reaches more posterior to the nasal aperture. The incisive foramina (Fig 13, Inc. F.) are anteroposteriorly shorter than those of *P. dasyuroides*.

Maxilla (Fig 13, Max.). Dorsal to the canine, the maxilla shows a weak anteroposterior convexity surrounding the long root of the tooth. At the level of P^2, the maxillae are conspicuously constricted transversally. The constriction extends to the palate ventrally. Posteriorly the lateral borders of the maxillae flare, reaching more than twice the width of that of the anterior constriction. This morphology is similar to the condition observed in *P. dasyuroides*. The anterior opening of the infraorbital canal (Fig 13, Infr. F.) is at the level of the mesial root of P^4, as in *P. dasyuroides*, but unlike the more anterior condition at the level of the interradicular space of P^3 seen in *Hyaenodon* [9]. Anterior to the infraorbital canal there is a shallow and round concavity, as described in *C. cayluxi* [9]. Superior to M^1 and M^2 is a well-marked fossa for the origin of nasolabialis. The inferior border of the orbit is horizontal, as in *C. cayluxi*. The posterior margin of the orbit does not slope dorsally as in *P. dasyuroides*. The orbit is relatively open on its dorsal and ventral margins, as in *C. cayluxi*, and differing from that of *P. dasyuroides* and *H. brachyrhynchus*.

Nasal (Fig 13, Na.). The nasal is a narrow and slightly triangular bone in dorsal view. Its posterior process terminates posterior to the anterior border of the orbit. Similarly long nasals are found in *P. dasyuroides*, contrasting with the shorter nasals of the proviverrines *Quercytherium* and *C. cayluxi*, the hyaenodontine *H. brachyrhynchus*, and the limnocyonine *Thereutherium*. There is no lateral expansion of the nasals directed toward the lacrimal as can be found in *P. dasyuroides* or *H. brachyrhynchus*. The nasal is mediolaterally convex at its anterior end, and flattens posteriorly.

Lacrimal (Fig 13, La.). The lacrimal is a large bone with a prominent anterior excursion onto the face beyond the medial margin of the orbit. The dorsal suture is on the dorsal border of the orbit, while the ventral suture extends onto the anterior margin of the zygomatic arch at the ventral border of the orbit. Unfortunately, the posterior border of the lacrimal is obscured by the fractures.

Jugal (Fig 13, J.). The fossa for nasolabialis described on the maxilla extends on the anterior portion of the jugal. It is weakly concave ventrally and dorsally. The jugal is almost straight with little lateral flare. Its lateroventral border is a rugose surface. This rugosity indicates a robust origin for the superficial layer of the masseter muscle, and suggests that the taxon was capable of powerful adduction of the mandible [9]. The suture with the squamosal gently slopes posteroventrally and the jugal almost reaches the preglenoid process (Fig 13, Pr. Gl. Pr.).

Frontal-Parietal (Fig 13, Fr.-Pa.). The two bones are well fused and no suture is evident between them. The sagittal crest (Fig 13, Sag. Cr.) is extremely well developed. On the posterior portion of the parietal, the sagittal crest represents more than half of the height of the skull,

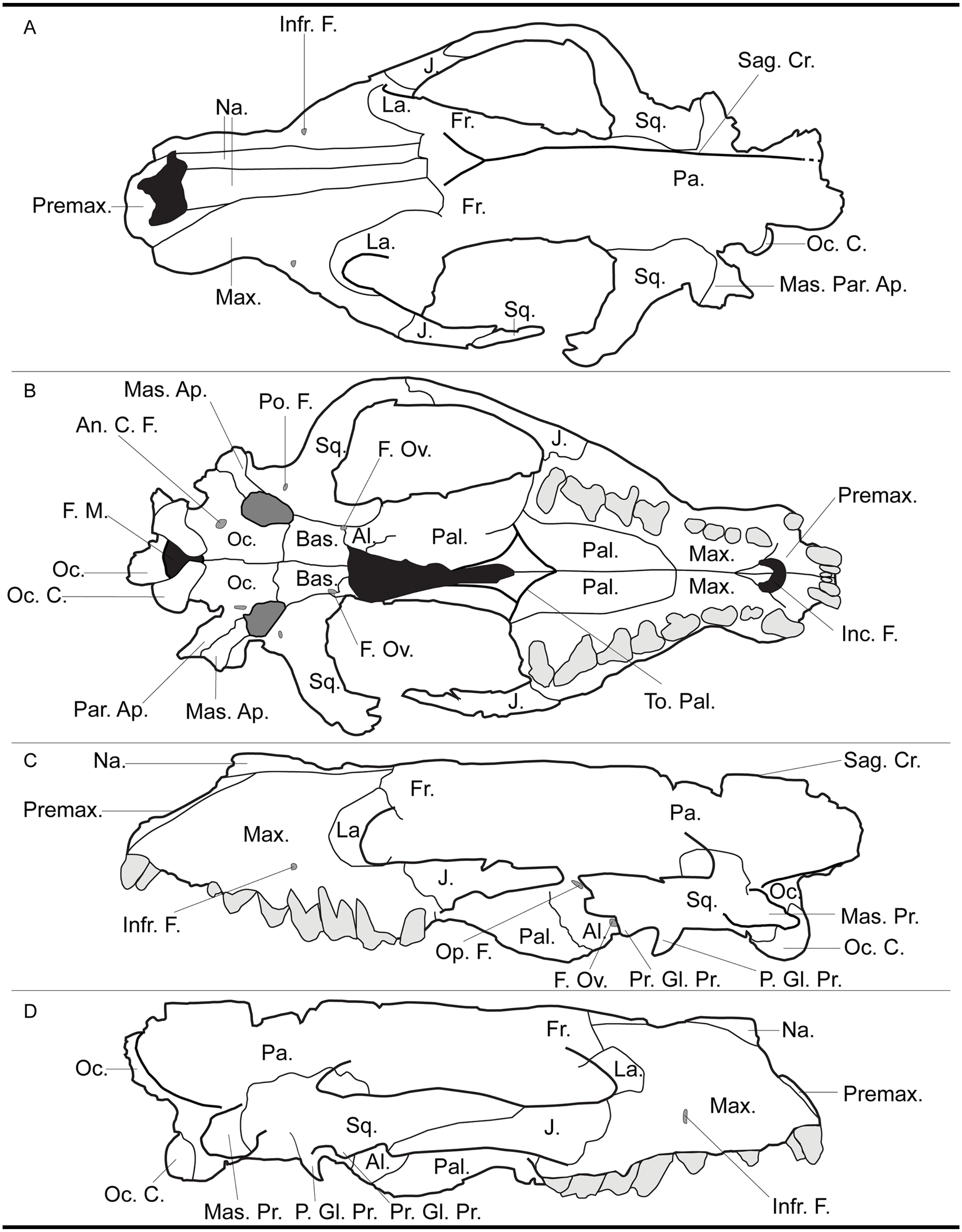
A
Infr. F.
Sag. Cr.
J.
La.
Na.
Fr.
Sq.
Pa.
Premax.
Fr.
La.
Oc. C.
Sq.
Mas. Par. Ap.
Max.
Sq.
J.
B
Mas. Ap.
Po. F.
An. C. F.
Sq.
F. Ov.
J.
Premax.
F. M.
Oc.
Bas.
Al.
Pal.
Pal.
Max.
Oc.
Oc.
Bas.
Pal.
Max.
Oc. C.
Inc. F.
F. Ov.
Sq.
Par. Ap.
Mas. Ap.
J.
To. Pal.
C
Na.
Sag. Cr.
Fr.
Premax.
Pa.
Max.
La
J.
Oc.
Sq.
Mas. Pr.
Infr. F.
Op. F.
Pal.
Al.
Oc. C.
F. Ov.
Pr. Gl. Pr.
P. Gl. Pr.
D
Fr.
Na.
Pa.
Oc.
La.
Premax.
Max.
Sq.
J.
Al.
Pal.
Oc. C.
Mas. Pr.
P. Gl. Pr.
Pr. Gl. Pr.
Infr. F.

Fig 8. Occipital view of the skull of *Kerberos langebadreae* gen. & sp. nov. (holotype, MNHN.F.EBA 517). A, occipital view. B, drawing of the occipital view. Abbreviations: F. M., foramen magnum; Lamb. Cr., lambdoid crest; Oc., occipital; Oc. C., occipital condyle; Oc. Cr., occipital crest; P. Gl. Pr., postglenoid process; Par. Ap., paroccipial apophysis; Sag. Cr., sagittal crest; Sq., squamosal.

doi:10.1371/journal.pone.0135698.g008

and forms a large area of origin for the temporal muscle, the other powerful adductor of the mandible.

Squamosal (Fig 13, Sq.). A roughened surface on superior surface of the posterior end of the zygomatic process marks the zygomatic origin of the temporal muscle. The deep masseter and zygomaticomandibularis also originate in this area but anteriorly to the temporal muscle. The squamosal bears both pre- and postglenoid processes (Fig 13, Pr. Gl. Pr. & P. Gl. Pr). The posterior pterygoid foramen (Fig 13, Po. F.) is preserved posterior to the postglenoid process. The otic region is severely crushed and hence hardly describable. The anteroposteriorly short promontorium is preserved and is isolated from the basioccipital by a large carotid foramen. A similar condition is found in *P. dasyuroides*.

Palatine (Fig 13, Pal.). The anterior border of the choana (posteriormost ventral contact between the palatines) almost reaches the level of the posterior end of the teeth row. Among Hyaenodonta, this feature is shared with the proviverrine *Allopterodon* and the limnocyonine *Thinocyon*. The position of the choana contrasts with the fused palatines in *P. dasyuroides* and *Apterodon macrognathus*, which finally open to the choana at the mid-point of the orbit. As in *P. dasyuroides*, there is a well-developed torus palatinus (Fig 13, To. Pal.) at the level of M^3. Anterior to M^3, the palatine is deeply excavated, for the occlusion with M_3. The palatines are as mesiodistally extended as they are in *P. dasyuroides*.

Alisphenoid (Fig 13, Al.). The morphology of the alisphenoid is difficult to define because of the numerous fractures in the specimen. However, the suture between the alisphenoid and palatine is clearly preserved at the posterior edge of the nasopalatine structure. A single foramen is preserved in the orbital region and we tentatively identify this as the optic foramen (Fig 13, Op. F.).

Basisphenoid (Fig 13, Bas.). The contacts between the alisphenoid, basisphenoid and squamosal are situated close to the foramen oval (Fig 13, F. Ov.). The basisphenoid is almost rectangular in ventral view (Fig 13B). The contact with the pterygoid bones is obscured by matrix.

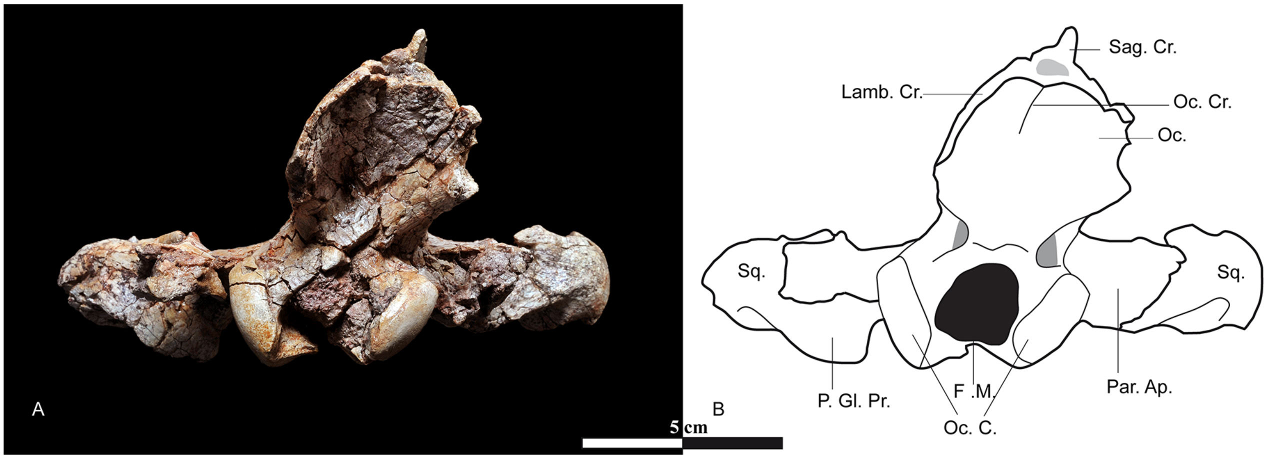

Fig 9. Upper dentition of *Kerberos langebadreae* gen. & sp. nov. (holotype, MNHN.F.EBA 517). A, right maxilla bearing P^1 and P^4-M^3 in occlusal view. B, left maxilla bearing P^2-M^3 in occlusal view.

doi:10.1371/journal.pone.0135698.g009

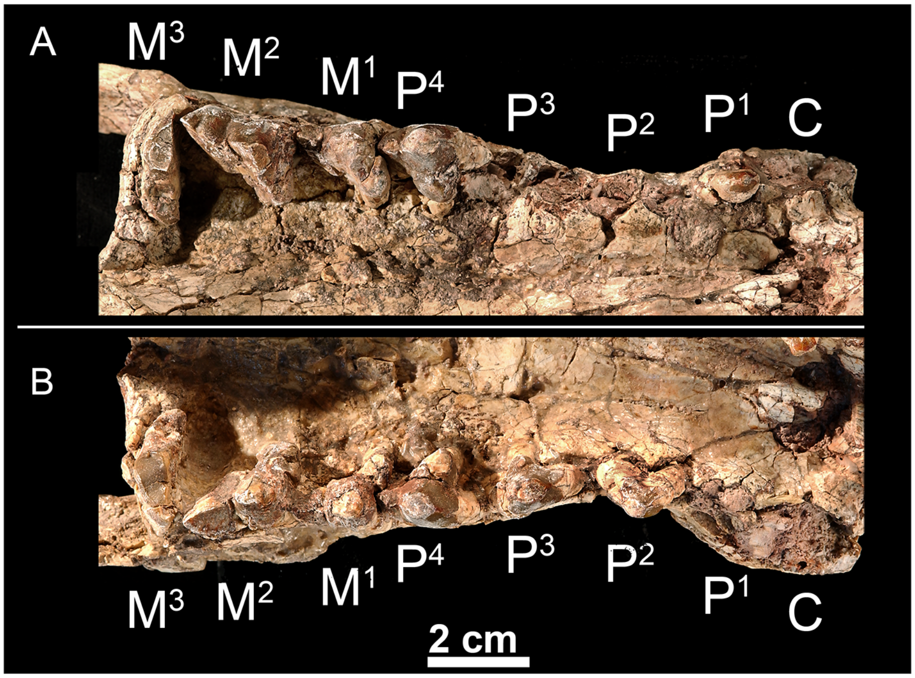

Fig 10. Mandible of *Kerberos langebadreae* gen. & sp. nov. (MNHN.F.EBA 518). A-C; MNHN.F.EBA 518a, left hemimandible bearing canine, M_2-M_3; A, occlusal view; B, lingual view; C, labial view. D-F, MNHN. F.EBA 518b, right hemimandible bearing P_3-P_4 and M_2-M_3; D, labial view; E, lingual view; F, occlusal view.

doi:10.1371/journal.pone.0135698.g010

Mastoid process (Fig 13, Mas. Pr.). The mastoid process is well preserved on the skull of *Kerberos*. However, the structures that contribute to its composition are difficult to distinguish. We tentatively delimitate its three components: the retrotympanic process of the squamosal, the mastoid apophysis (Mas. Ap.), and the paroccipital apophysis (Par. Ap.). The paroccipital apophysis extends distally.

Occipital region (Fig 13, Oc.). The supraoccipital, exoccipital, and basioccipital bones are all thoroughly fused to each other. The lambdoidal crests are incomplete and do not extend to the mastoid process, a feature characteristic of Hyainailouridae (see above). Instead, the lambdoidal crests converge ventrally, forming a concave, oval posterior surface above the foramen magnum (Fig 8). They are directed ventrally toward the foramen magnum (Fig 13, F. M.), forming a clover-leaf-like shape (Fig 8). This morphology, notably found in *P. dasyuroides*, is used by Polly [38] to diagnose the Hyainailourinae ("Pterodontinae" *sensu* Polly [38] = Hyainailouridae). The anterior condyloid foramina are clearly visible and distal to the foramina, the occipital condyles (Fig 13, Oc. C.) are large and positioned around the foramen magnum.

Upper dentition. The skull (MNHN.F.EBA 517) displays three upper incisors. The three incisors are compressed transversally. The I^1 is the smallest. I^2 and I^3 are simple and conical. I^3 is distinctly larger than the two other incisors and is semicaniniform.

Unfortunately, the crowns of the canines are not preserved.

The P^1, which is double-rooted, is formed by a single pointed cusp (= paracone) (Fig 9). No parastyle is present. The metastyle is very poorly developed. The crown of the P^2 is unknown but the alveoli indicate that it is double-rooted like the P^1. The P^3 is triple-rooted with a heavily worn parastyle. The metastyle is short and low. A small protocone is present; it is lingually located relative to the paracone. The P^4 is the largest premolar. The parastylar region is worn out as on the previous premolars, but it was certainly well developed, which is typical of the

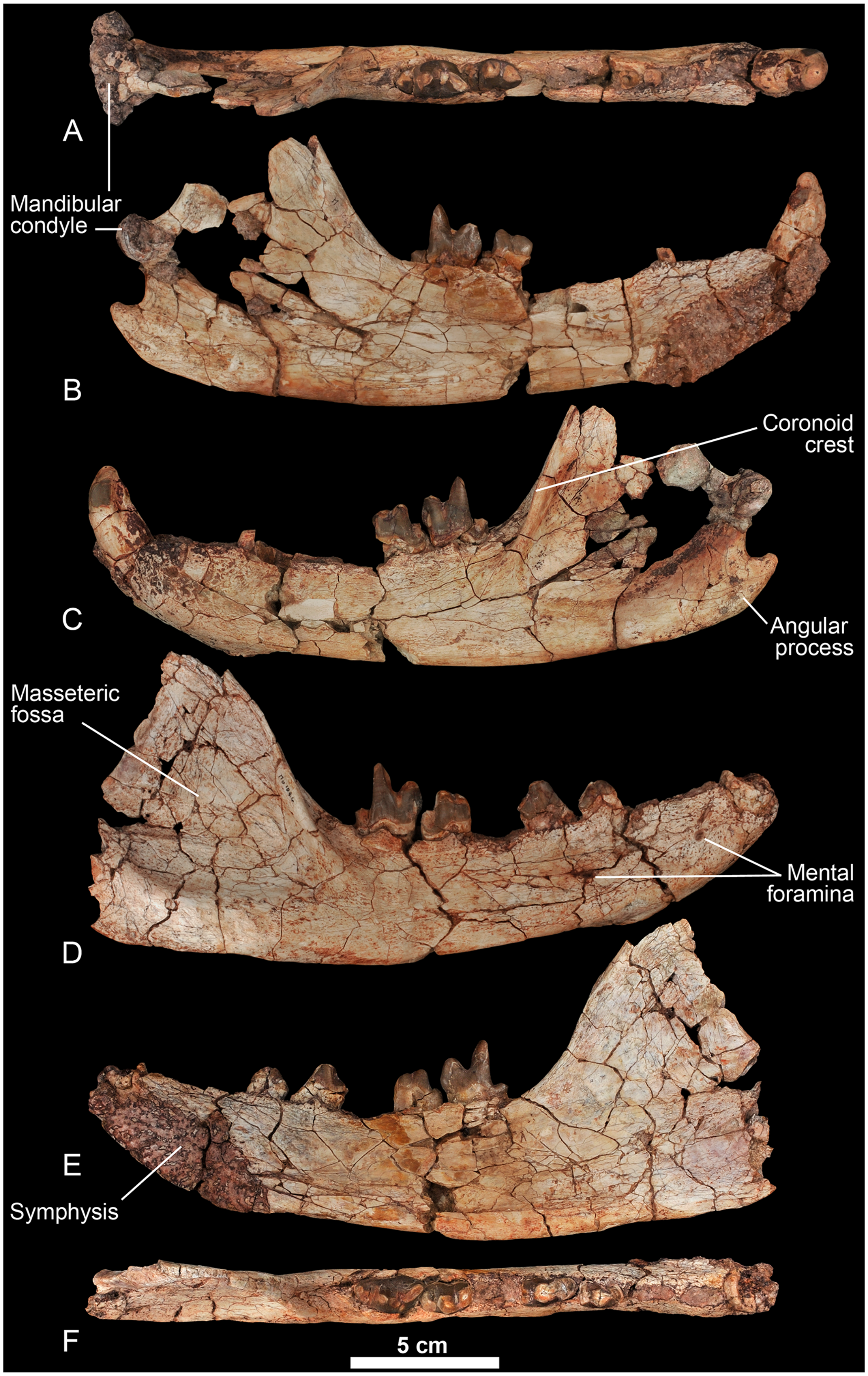
A
Mandibular condyle
B
Coronoid crest
C
Angular process
Masseteric fossa
Mental foramina
D
E
Symphysis
F
5 cm

Fig 11. Postcranial elements of *Kerberos langebadreae* gen. & sp. nov. A-B, fibula (right, MNHN.F.EBA 520); A, dorsal view; B, plantar view. C-E, calcaneus (right, MNHN.F.EBA 522); C, dorsal view; D, medial view; E, lateral view. F-G, astragalus (right, MNHN.F.EBA 521); F, plantar view; G, dorsal view.

doi:10.1371/journal.pone.0135698.g011

hyainailourine P^4. The protocone is much more developed than on the P^3. The protocone does not bear accessory cusps.

The three upper molars are known. The M^1 is characterized by a short parastyle, a small, mesially located protocone relative to the paracone and metacone, and a long, mesiodistally aligned metastyle. The paracone and metacone are fused into an amphicone [9]. No groove

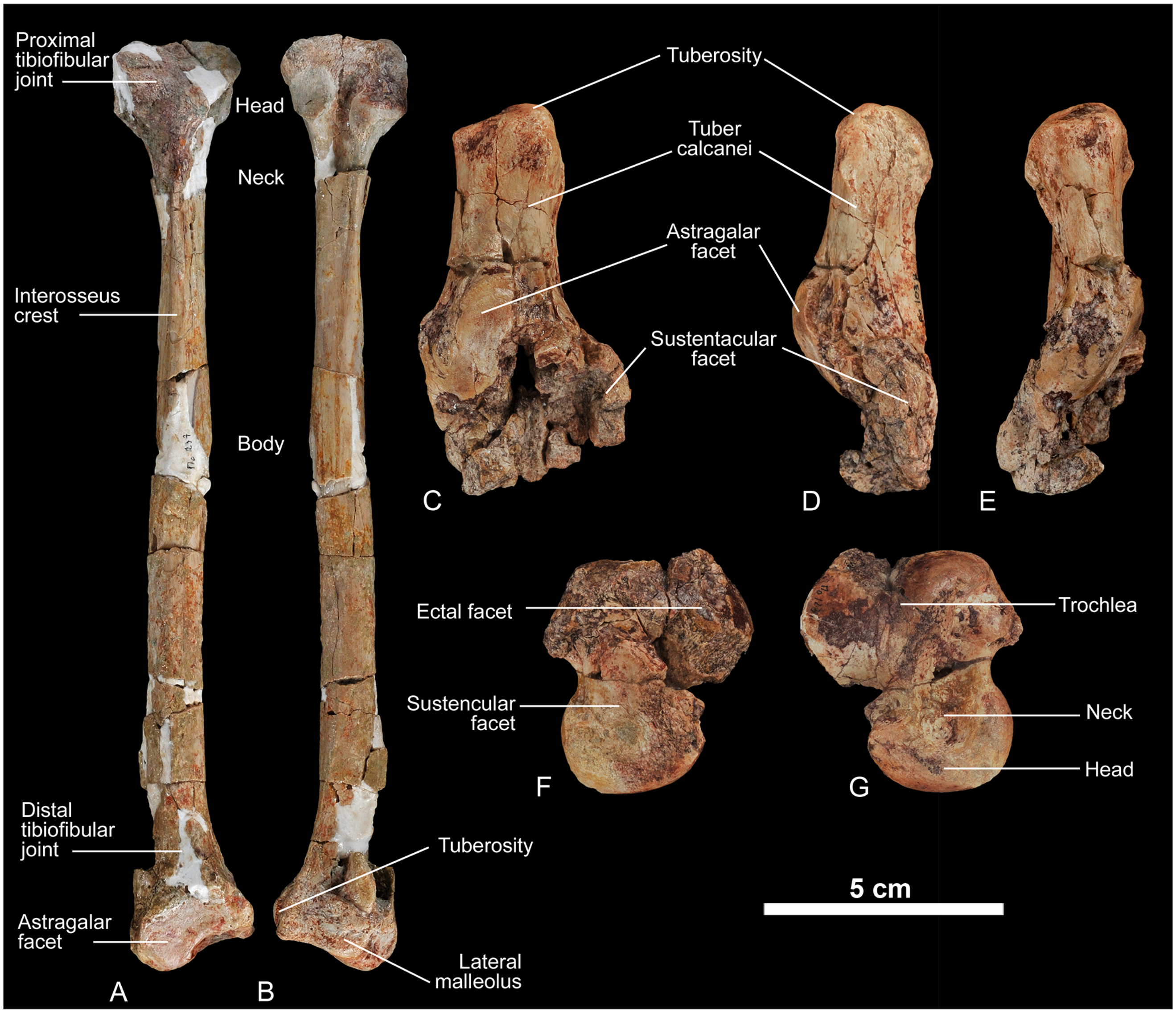

Fig 12. Postcranial elements of *Kerberos langebadreae* gen. & sp. nov. A-B, metatarsal I (MNHN.F.EBA 523); A, dorsal view; B, plantar view. C-D, metatarsal II (right MNHN.F.EBA 524); C, dorsal view; D, plantar view. E-F, metatarsal III (MNHN.F.EBA 525); E, dorsal view; F, plantar view. G-H, metatarsal II (left, MNHN.F.EBA 526); G, dorsal view; H, plantar view. I-J, middle phalanx (MNHN.F.EBA 528); I, dorsal view; J, plantar view. K-L, middle phalanx (MNHN.F.EBA 527); K, dorsal view; L, plantar view.

doi:10.1371/journal.pone.0135698.g012

Table 2. Dental measurements (in mm.) of *Kerberos langebadreae* gen. & sp. nov.

Locus		n	OR	Mean	Locus		n	OR	Mean
I^1	L	2	0.68–0.92	0.8					
	W	2	1.50–1.55	1.53					
I^2	L	2	0.44–0.59	0.52					
	W	2	0.91–0.98	0.93					
I^3	L	2	0.18–0.28	0.23					
	W	2	0.61–0.67	0.64					
C	L	2	2.17–2.19	2.18	C	L	2	1.98–2.12	2.05
	W	2	1.54–1.63	1.59		W	2	1.76	1.76
P^1	L	2	1.07*-1.21	1.14	P_1	L	2	1.62*-1.67*	1.65
	W	2	0.65–0.69	0.67		W	2	0.75*-0.77*	0.76
P^2	L	2	1.35–1.64*	1.5	P_2	L	2	1.52*-1.68*	1.60
	W	2	0.64–0.65*	0.65		W	2	0.68*-0.86*	0.77
P^3	L	2	1.83*-1.86	1.85	P_3	L	2	1.62–1.63*	1.63
	W	2	0.85*-1.18	1		W	2	0.72*-0.91	0.82
P^4	L	2	2.08–2.13	2.11	P_4	L	1	2.03	-
	W	2	1.63–1.76	1.7		W	1	1.01	-
M^1	L	2	1.91–1.95	1.93	M_1	L	1	1.57*	-
	W	2	1.75–1.84	1.8		W	1	1.16*	-
M^2	L	2	2.30–2.39	2.35	M_2	L	2	1.87–2.09	1.98
	W	2	1.81–2	1.9		W	2	1.15–1.19	1.17
M^3	L	2	0.89–0.95	0.92	M_3	L	2	2.50–2.66	2.58
	W	2	2.44–2.53	2.49		W	2	1.36–1.37	1.37

*based on roots. Abbreviations: L, mesiodistal length; n, number of teeth measured; OR, Observed range; W, linguolabial width. Observed range.

doi:10.1371/journal.pone.0135698.t002

delimitates the paracone from the metacone though the apex of each cusp would have been distinct. The M^1 has no cingulum, which is also the case on the M^2 and M^3. The M^2 has a generally similar morphology to the M^1. However, the parastylar region and the metastyle are slightly more developed with heavy distal abrasion where the M_3 would have shorn against the metastyle. The M^3 is transversally elongate. The preparacrista is long. The metacone is absent. The protocone is short and narrow. The P^4, M^1 and M^2 are implanted obliquely. Rather than being positioned parallel to the buccal margin, the cusps of these teeth angle slightly posteriorly and laterally. Such a condition is found in the hyainailourine *P. dasuryoides*.

Dentary. One mental foramen is present below the P_1 and another is present below the P_4 (Fig 10). The symphysis extends posteriorly up to the distal root of the P_3. The ventral margin of the mandible is slightly convex, especially posterior to the tooth row. The mandibular condyle is slightly higher than the tooth row. It is cylindrical and mediolaterally elongate. The coronoid crest arises at a 45° angle relative to the horizontal ramus. A deep fossa for insertion of the temporal muscle is present along the anterior margin of the crest. This fossa is particularly prominent at the base and extends below the distal root of the M_3. The angular process is curved sharply dorsally and is relatively thin. The masseteric fossa is deep and wide.

Lower dentition. The holotype does not preserve the lower incisors. The crown of P_1 is not preserved, but its presence is confirmed by the presence of its single alveolus. The P_1 is separated from the P_2 by a small diastema. The P_2 is double-rooted and is only slightly longer than the P_1 (Table 2). The double-rooted P_3 is known by one worn tooth. The presence of a slight precingulid indicates the probable presence of a small paraconid. The talonid is large and is

Table 3. Measurements (in mm.) of postcranial elements of *Kerberos langebadreae* gen. & sp. nov.

**Measurements of the fibula**		
Total length		193.3
Anteroposterior depth of the head		27*
Transverse width of head		18.3
Mid-shaft anteroposterior diameter		12.1
Mid-shaft transverse diameter		10.9
Anteroposterior depth of the distal epiphysis		26.7
Transverse width of the distal epiphysis		19.7
**Measurements of the calcaneum**		
Total length		-
Length of the tuber calcanei (up to the ectal facet)		40.7
Width of the tuber at mid-length		21.9
Transverse width of the ectal facet		13.5
Proximodistal length of the ectal facet		38.6
**Measurements of the astragalus**		
Total length		52.6
Maximum transverse length		45.6
Trochlea length		35.3
Trochlea width		26.5
Head width		30.6
**Measurements of the metatarsals and middle phalanges**		
Metatarsal I	Length	57.5
	Transverse width at mid-length	9.6
Metatarsal II (left)	Length	76.5
	Transverse width at mid-length	11.4
Metatarsal II (right)	Length	76.9
	Transverse width at mid-length	11.9
Metatarsal III	Length	78.0
	Transverse width at mid-length	11.6
Middle phalange MNHN.EBA 528	Length	26.7
	Transverse width at mid-length	12.2
Middle phalange MNHN.EBA 527	Length	31.7
	Transverse width at mid-length	14.0

*, estimated.

doi:10.1371/journal.pone.0135698.t003

constituted by the hypoconid. The P_4 is larger than the previous premolars. The relatively small paraconid and the large, hypoconid-bearing talonid recall the morphology of P_3.

The M_1 is unknown but was clearly smaller than the P_4 and the distal molars. The metaconid is absent on the M_2 and M_3. The talonid on M_2 is simple; only the hypoconulid is clearly visible at the distal part of the talonid. A small anterior keel is visible on the buccal aspect of the paraconid. The M_3 differs from the M_2 by its larger size and shorter talonid. The talonid of M_3 is as simple as the talonid of M_2.

Description of the postcranial elements. Fibula. The entire fibula is preserved, including both proximal and distal epiphyses. The fibula is partially reconstructed, but this reconstruction does not have any impact on the features described herein. There is no indication that the fibula was fused in any way to the tibia (Fig 11). A tendinal sulcus can be traced on the shaft. The proximal head of the fibula exhibits a semilunar facet for articulation with the tibia. The

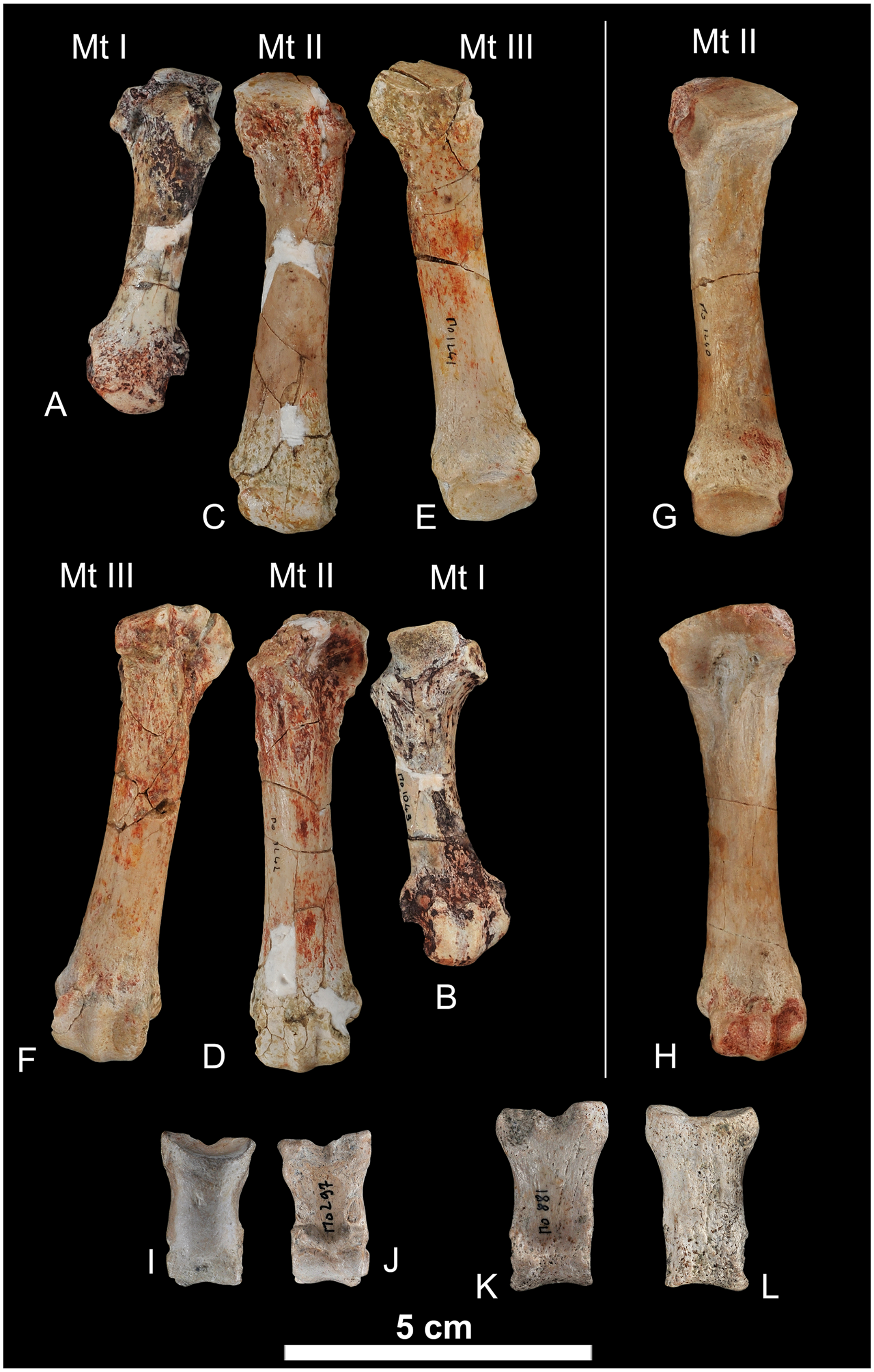
Mt I
Mt II
Mt III
Mt II
A
C
E
G
Mt III
Mt II
Mt I
B
F
D
H
I
J
K
L
5 cm

Fig 13. Drawings of the skull of *Kerberos langebadreae* gen. & sp. nov. (holotype, MNHN.F.EBA 517). A, dorsal view; B, ventral view; C, left lateral view; D, right lateral view. Abbreviations: Al., alisphenoid; An. C. F., anterior condyloid foramen; Bas., basisphenoid; F. M., foramen magnum; F. Ov., foramen oval; Fr., frontal; Inc. F., incisive foramen; Infr. F., infraorbital foramen; J., jugal; La., lacrimal; Mas. Ap., mastoid apophysis; Mas. Par. Ap., mastoid-paroccipital apophysis; Mas. Pr., mastoid process; Max., maxilla; Na., nasal; Oc. C., occipital condyle; Oc., occipital; Op. F., optic foramen; P. Gl. Pr., postglenoid process; Pa., parietal; Pal., palatine; Par. Ap., paroccipial apophysis; Po. F., posterior pterygoid foramen; Pr. Gl. Pr., preglenoid process; Premax., premaxilla; Sag. Cr., sagittal crest; Sq., squamosal; To. Pal., torus palatinus.

doi:10.1371/journal.pone.0135698.g013

distal head is broad and possesses two facets that articulate with the calcaneus and the astragalus. The former calcaneo-fibular joint is characteristic of "Creodonta" (e.g., in *Hyaenodon* and *Oxyaena*).

Calcaneus. The calcaneus is not well preserved (Fig 11). The distal portion is particularly crushed and difficult to interpret accurately. The tuber calcaneum is long and thickened. The facet for the articulation with the cuboid seems to have been laterally sloping (as in *Hyaenodon*), because its distal portion is diagonal in proximal view. The sustentacular facet is situated plantar to the astragalar facet. The shape of the sustentacular facet cannot be described, because the bone is damaged in this region. The astragalar facet is elongate and ovoid with a slight proximal inflection that extends onto the tuber calcanei. The astragalar facet is not kidney-shaped as in *Hyaenodon*.

Astragalus. The astragalus is crushed, especially on its plantar aspect (Fig 11). The trochlea–as well as the head of the astragalus–are asymmetrical. The trochlea is grooved, but rather shallow compared to that of *Hyaenodon*. The head of the astragalus is widened and transversally flat. In proportion to the trochlea, the head appears unusually large even when compared with the astragalus of the large bodied hyainailourine *Hyainailouros*. Compared to *Hyaenodon* the caput astragali is not rounded, but transversally flattened, a condition also found in *Hyainailouros*. The neck is robust and short. The astragalar neck is relatively shorter and the trochlea shallower than in *Hyaenodon*. The plantar articular facets for the calcaneus are too poorly preserved to allow any comment.

Metatarsal I. The robust first metatarsal is reduced in length relative to the other metatarsals (Fig 12).

Metatarsal II. The second metatarsal, which is known for both the right and left feet, is subequal in length to the third metatarsal and is short and robust. The proximal articulation facet is proximo-distally elongated. It is asymmetrical with a medial indentation of its shaft. The trochlea is slightly curved. This bone is shorter and wider than the second metatarsal of *Hyaenodon*. The trochlea is not globular as in *Hyaenodon* and all recent carnivorans.

Metatarsal III. The third metatarsal is broadly comparable to the second metatarsal except for the morphology of the proximal articulation. The plantar portion of the proximal articulation facet is laterally compressed while the dorsal portion expands dorsally with lateral and medial facets that would have overlapped the metatarsals lateral and medial to the fourth metatarsal.

In general, the columnar shafts of the metatarsals and globular proximal articulations are similar to the metatarsals of *Hyainailouros* [4] and are completely unlike the more gracile metatarsals present in the digitigrade foot of *Hyaenodon* [83]. When articulated, the three metatarsals are slightly divergent distally.

Middle phalanx. Two middle phalanges have been recovered. They do not have the same size, indicating that they correspond to distinct digits. This element is short (Fig 12) and wide. The proximal articulation facet is heart-shaped and concave with a plantar indentation. The distal trochlea is asymmetrical. Its shaft is symmetrical and preserves distinct flexor tubercles. Compared to *Hyaenodon* the middle phalanx is more robust, comparable to the middle

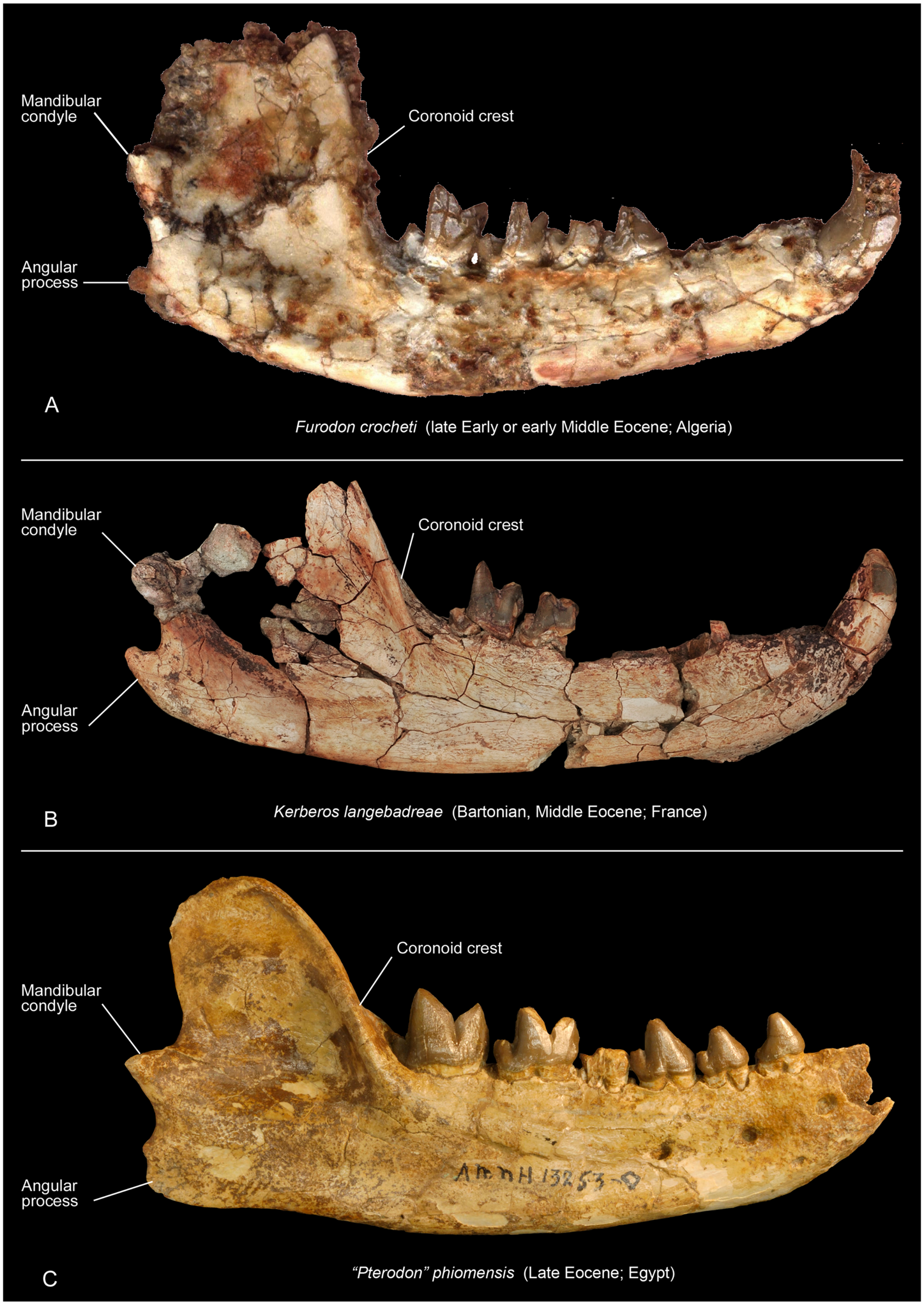
Mandibular condyle
Coronoid crest
Angular process
A
Furodon crocheti (late Early or early Middle Eocene; Algeria)
Mandibular condyle
Coronoid crest
Angular process
B
Kerberos langebadreae (Bartonian, Middle Eocene; France)
Coronoid crest
Mandibular condyle
Angular process
C
"Pterodon" phiomensis (Late Eocene; Egypt)

Fig 14. Comparison of the hemimandibles in labial view of hyainailourines. *Furodon crocheti* (A, left inverted, HGL 50bis-56; HGL, Hammada Gour Lazib, Algeria, Université Montpellier 2, France), *Kerberos langebadreae* gen. & sp. nov. (B, holotype, left inverted, MNHN.F.EBA 518b) and "*Pterodon*" *phiomensis* (C, right, AMNH 13253; AMNH, American Museum of Natural History, New York, USA). Not to scale

doi:10.1371/journal.pone.0135698.g014

phalanx of recent ursids. The middle phalanx of *Hyainailouros* is more asymmetrical with a unilateral indentation of the shaft [4].

Comparison and discussion. This specimen is especially noteworthy for its tremendous size. Indeed, no hyaenodontid of such a size is presently known for the Bartonian, making *Kerberos* one of the largest carnivores known from the Eocene of Europe–only younger *Paraptero-don* is slightly larger. In addition to considerations of body size, the material facilitates an examination of the locomotion of the earliest hyainailourines.

The dental formula of the new taxon is reminiscent of the earliest proviverrines, sinopines, arfiines, and indohyaenodontines–as well as the earliest placentals–in the presence of three upper incisors, four premolars and three molars. However, the reduced molar talonids of the new taxon clearly distinguishes it from the contemporaneous proviverrines, which retain three-cusped, basined talonids. The reduction of the talonid is shared with the hyaenodontines. The specimen differs from the hyaenodontines in the morphology of its nuchal crest, which does not extend to the mastoid process, and differs from *Hyaenodon* in the subequal length of the paraconid and protoconid. These features are characteristic of the Hyainailouridae [38]. The new taxon also displays several dental features of the Hyainailourinae such as are more reduced talonid on M_3 relative to that of M_2, the absence of metaconids on the molars, and the connate paracone and metacone [38,70].

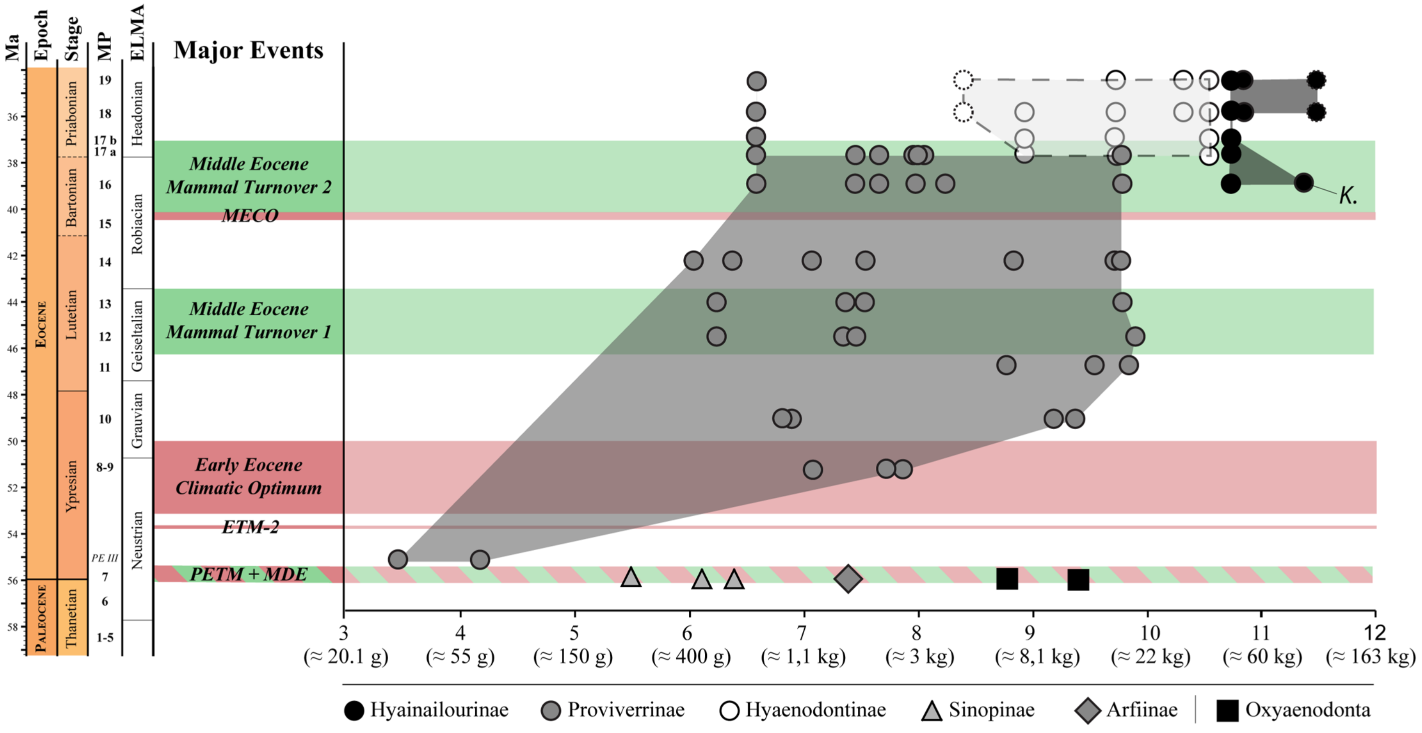

Fig 15. Values of the ln of body mass based on molar dimensions of European oxyaenodontidans and European hyaenodonts from MP7 to MP19. Values given in Table 4. Abbreviation: *K.*, *Kerberos*.

doi:10.1371/journal.pone.0135698.g015

Table 4. Mean tooth measurements and body mass estimations. The data concerning the Oxyaenodonta, Sinopinae and Arfiinae and Proviverrinae are from Solé et al. (2014) [17]. Abbreviations: Hyae, Hyaenodontinae; Hyai, Hyainailourinae; (M_1-M_3)L, M_1 to M_3 length. This stratigraphic repartition of *H. rossignoli* was chosen because the locality of Memerlin (France) where it is recorded is either MP18 or MP19 [93].

MP	Species	Taxon	(M_1-M_3)L (mm)	Mass (kg)	LnMass	References
**16**	*Kerberos langebreae*	Hyai	60.9	87.69	11.38	Present paper
	Paroxyaena galliae	Hyai	50.9	46.72	10.75	[9]
**17a**	*Paroxyaena galliae*	Hyai	50.9	46.72	10.75	[9]
	Hyaenodon brachyrhynchus	Hyae	38.2	17.06	9.74	[9]
	Hyaenodon minor	Hyae	30.4	7.65	8.94	[9]
	Hyaenodon requieni	Hyae	48.1	38.30	10.55	[9]
**17b**	*Paroxyaena galliae*	Hyai	50.9	46.72	10.75	[9]
	Hyaenodon brachyrhynchus	Hyae	38.2	17.06	9.74	[9]
	Hyaenodon minor	Hyae	30.4	7.65	8.94	[9]
	Hyaenodon requieni	Hyae	48.1	38.30	10.55	[9]
**18**	*Pterodon dasyuroides*	Hyai	52.35	51.56	10.85	[9]
	Parapterodon lostangensis	Hyai	62.82	97.78	11.49	[9]
	*Paroxyaena pavlovi**	Hyai	50.9	46.72	10.75	[9]
	Hyaenodon brachyrhynchus	Hyae	38.2	17.06	9.74	[9]
	Hyaenodon heberti	Hyae	45.09	30.53	10.33	[9]
	Hyaenodon minor	Hyae	30.4	7.65	8.94	[9]
	Hyaenodon requieni	Hyae	48.1	38.30	10.55	[9]
	Hyaenodon rossignoli	Hyae	26.1	4.48	8.41	[9]
**19**	*Pterodon dasyuroides*	Hyai	52.35	51.56	10.85	[9]
	Parapterodon lostangensis	Hyai	62.82	97.78	11.49	[9]
	*Paroxyaena pavlovi**	Hyai	50.9	46.72	10.75	[9]
	Hyaenodon brachyrhynchus	Hyae	38.2	17.06	9.74	[9]
	Hyaenodon heberti	Hyae	45.09	30.53	10.33	[9]
	Hyaenodon requieni	Hyae	48.1	38.30	10.55	[9]
	Hyaenodon rossignoli	Hyae	26.1	4.48	8.41	[9]

**Paroxyaena pavlovi* is here considered to be equivalent in size to *Paroxyaena galliae* because the M^1 of *P. pavlovi*, the only molar presently known for the taxon, is similar in size to the M^1 of *P. galliae*.

doi:10.1371/journal.pone.0135698.t004

The African genus *Metapterodon*, which is morphologically close to hyainailourines, was referred to Koholiinae by Solé et al. [6]; it was previously referred to Hyainailourinae [69–70]. *Kerberos* notably differs from *Metapterodon* by the presence of a P_1, a short and low metastyle on P^4, presence of a small protocone on P^3, and absence of an ectoflexus on each upper molar. These features are shared with the hyainailourines.

The Eocene-Oligocene hyainailourines are represented in Africa by four distinct genera: *Furodon* and *Parvavorodon* from late Early or early Middle Eocene of Algeria, and *Akhnatenavus* and *Pterodon* from the Fayum Depression of Egypt [6,37,69]. Three hyainailourine genera are known for the same period in Europe: *Paroxyaena*, *Parapterodon*, and *Pterodon*. Only one hyainailourine, *Hemipsalodon*, is known in the Eocene of North America (see below for discussion referring it to Hyainailourinae).

Kerberos shares with *Furodon* (late Early or early Middle Eocene; Algeria), the oldest hyainailourine, the presence of a single-rooted P_1; this tooth is larger than P_1 in the younger *Pterodon* and *Akhnatenavus*. However, *Kerberos* differs from *Parvavorodon* and *Furodon* by the loss of the metaconid and a more reduced talonid on its lower molars and more connate paracone and metacone on its upper molars. These features are shared with the Late Eocene and

Oligocene hyainailourines (e.g., *Akhnatenavus Paroxyaena*, *Parapterodon*, and *Pterodon*). *Kerberos* and *Furodon* share a similar mandibular morphology with the mandibular condyle located superior to the tooth row, a relatively superior placement of the angular process, and a steeply inclined ascending ramus and deepened fossa for the insertion of the temporal muscle on the anterior portion of coronoid crest (Fig 14).

The African genus *Akhnatenavus* is presently represented by only one species: the Oligocene *Akhnatenavus leptognathus* [69]. *Kerberos* differs from *Akhnatenavus* by exhibiting less pronounced diastemata between the premolars than *Akhnatenavus*, and more simplified molars (e.g., reduced talonid). All these features are derived in Hyainailourinae. Based on dental measurements, *Kerberos* is 40% larger than *Akhnatenavus*. *Kerberos* thus appears more derived than *Akhnatenavus*. However, *Kerberos* also differs from *Akhnatenavus* in the retention of an important primitive feature: a more developed P_1.

Kerberos and *Pterodon dasyuroides* are the only Eocene hyainailourines currently represented by skulls. Both have anteroposteriorly elongate skulls with relatively long rostra and neurocrania. *Pterodon* includes four European and African species: *Pterodon africanus* Andrews, 1903 [84] (Oligocene), *Pterodon phiomensis* Osborn, 1909 [56] (Oligocene), *Pterodon syrtos* Holroyd, 1999 [69] (Oligocene), and the European *Pterodon dasyuroides* Blainville, 1839 [77] (Late Eocene), which is the type species. In the analysis presented here, the genus is paraphyletic (see below). The teeth of *Kerberos* are generally similar to those of "*Pterodon*", but *Kerberos* differs in its larger P_1, P^1, and M^3. The P_1 is often absent in *P*. *dasyuroides* [9] and small in "*P*." *africanus* and "*P*." *phiomensis*. The talonids on the molars of *Kerberos* are relatively longer, and the diastema between P_1 and P_2 is longer than in "*Pterodon*" species. The mandible of *Kerberos* is more primitive than that of "*Pterodon*" species in having a more dorsal mandibular condyle, a more dorsal angular process, a more steeply inclined and taller coronoid crest, and deeper insertions of the temporal muscle on the anterior margin of the coronoid process (Fig 14). The mandibular features are reminiscent of those of *Furodon*, which is–with *Parvavorodon*–the oldest hyainailourine presently known (Fig 14). Finally, the metacone and paracone on the molars are more fused, a more derived feature than the less complete fusion present in *P*. *dasyuroides*.

Kerberos further differs from the European *Pterodon dasyuroides* in the presence of large P_1 and P^1, and an unreduced number of upper incisors (only two upper incisors are present in *P*. *dasyuroides*); these features are primitive among hyainailourines. *Kerberos* also differs from *P*. *dasyuroides* in its larger protocone on P^3, and reduced parastylar area; these are derived features in hypercarnivores. Finally, based on dental measurements and skull measurements, *Kerberos* differs from *P*. *dasyuroides* by its larger size (10–14%).

In her study of the European hyaenodontids from the Late Eocene to Late Oligocene, Lange-Badré [9] described three genera that she considered to be close to *Pterodon*: *Parapterodon*, *Paroxyaena*, and *Schizophagus*. Since this study, *Schizophagus* has been synonymized with *Paroxyaena*, which is considered to be a peculiar hyainailourine [8,85]. *Paroxyaena* is known in Robiac's fauna (reference locality of MP16 reference-level) [27]. Consequently, *Kerberos* and *Paroxyaena* are nearly coeval. Sudre [28] noted that the "*Castrais*" fauna could be slightly older than the Robiac fauna, *contra* Astruc et al. [29].

Kerberos shares with *Parapterodon* and *P*. *dasyuroides* but not with *Paroxyaena* the presence of a high, sharp P^4, and a more fused paracone and metacone on the upper molars. *Kerberos* differs from *Parapterodon* in the primitive presence of a large P^1 –this tooth is even vestigial in *Parapterodon*–and a primitively large M^3. *Kerberos* further differs from *Parapterodon* by having a more reduced parastylar region, and a more reduced protocone on upper molars, generally derived features among lineages that acquire hypercarnivorous dentition [63,86]. *Kerberos* shares with *Paroxyaena* the presence of three upper incisors, but *Kerberos* differs from

Paroxyaena by the presence of P_1, a less reduced P^1, a less developed protocone on P^3, larger M^3 (all considered primitive features), more reduced talonids on lower molars, fusion of the paracone and metacone, and reduced protocones on the upper molars (generally derived features).

Kerberos differs from the North American *Hemipsalodon* in its smaller size (the skull of *Hemipsalodon* is 45 cm long), its mesiodistally elongate skull (notably in the rostrum and the neurocranium), the distal elongation of the mastoid process (primitive features), and a more closely appressed paracone and metacone on the molars (derived feature).

In summary, the taxon described here departs from other members of the hyainailourine subfamily in featuring a distinctive mixture of primitive and derived features. Thus, we refer this taxon to the new genus, *Kerberos*. Its discovery increases the taxonomic and morphological diversity of the hyainailourines, especially in Europe.

Discussion

Ecology of *Kerberos*

The body size of an animal is one of the most important factors in determining its role in its ecosystem. Body size influences dietary preference, predatory behavior, and niche partitioning [87–88]. It is directly related to an organism's biomechanical and physiological demands. Several methodologies have been proposed for determining the body mass in fossil mammals. Several of them have been applied to hyaenodonts. Depending on the methodology, they correspond to regressions based on dental, cranial or postcranial dimensions [21,89–92]. Most of these studies use extant taxa to correlate known body size to osteological measurements that can be collected from related extinct taxa. The reconstruction of body sizes for Hyaenodonta has been problematic because the entire group is extinct, making direct regressions difficult to apply to the group. Moreover, as mentioned by Van Valkenburgh [89], dentally or cranially derived body size estimates based on extant carnivorans produce unreasonably large values in hyaenodonts because they have relatively large crania compared to carnivorans.

With these caveats in mind, we estimate the body mass of *Kerberos langebadreae* using three methodologies. (1) The methodology of Morlo [21], which is based on dental dimensions (length of the molars), provides an estimation of 85–90 kg (Table 4). (2) The methodology of Van Valkenburgh [89], based on the skull length, estimates the body mass of *K. langebadreae* to be about 277 kg. (3) The methodology of Tsubamoto [92], which is based on the dimensions of the astragalus results in ranges from 60 kg to 269 kg (length Li1) and from 49 to 199 kg (length Ar1). The median body mass of these estimates is 140 kg.

We present a comparison of the estimated body mass of *Kerberos* with those of proviverrines from MP7 to MP19 and contemporaneous hyainailourines and hyaenodontines (Fig 15; Table 4). We only use the methodology of Morlo [21] for this comparison because it is based on dental measurements, and cranial and astragalar dimensions are not available for the entire sample. During their evolution, proviverrines never have exceeded 20 kg (see Solé et al. [17]). The two oldest hyainailourines recorded in Europe, *Kerberos* and *Paroxyaena*, are clearly distinguished from endemic proviverrines by their much larger size. Thus, neither *Kerberos* nor *Paroxyaena* was likely in direct competition with proviverrines. Conceivably, the smaller proviverrines may have even been their prey.

It is noteworthy that the body mass range of contemporaneous European hyaenodontines (4–39 kg) partially overlaps with that of proviverrines (0.03–20 kg) (Fig 15). Morlo [21] reconstructed the large proviverrines (*e.g.*, *Oxyaenoides*) as cursorial hypercarnivorous predators; because this corresponds to the locomotion of *Hyaenodon*, they thus may have belonged to the same ecological niche. Additionally, one can note that the appearance of Hyaenodontinae in

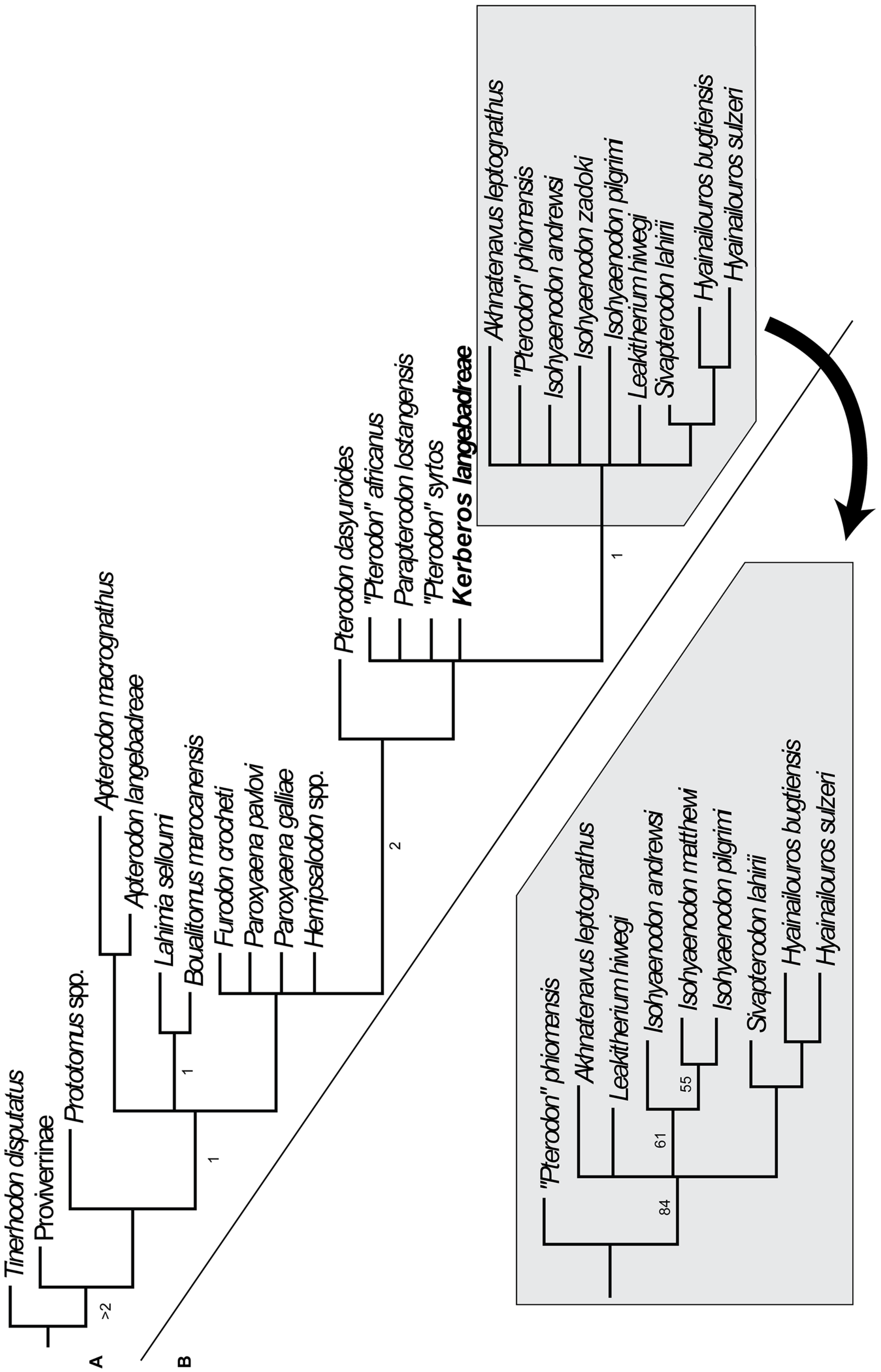
A
B
Tinerhodon disputatus
Proviverrinae
Prototomus spp.
Apterodon macrognathus
Apterodon langebadreae
Lahimia selloumi
Boualitomus marocanensis
Furodon crocheti
Paroxyaena pavlovi
Paroxyaena galliae
Hemipsalodon spp.
Pterodon dasyuroides
"Pterodon" africanus
Parapterodon lostangensis
"Pterodon" syrtos
Kerberos langebadreae
Akhnatenavus leptognathus
"Pterodon" phiomensis
Isohyaenodon andrewsi
Isohyaenodon zadoki
Isohyaenodon pilgrimi
Leakitherium hiwegi
Sivapterodon lahirii
Hyainailouros bugtiensis
Hyainailouros sulzeri
Isohyaenodon matthewi
>2
1
1
2
1
84
61
55

Fig 16. Consensuses of the first phylogenetic analysis. A: strict consensus (135 steps long; CI 0.52; RI 0.68) of the first phylogenetic analysis of the hyainailourines which includes body size character with indications of the Bremer support values. B: relationships among several hyainailourines in the majority rule consensus (120 steps long; CI 0.59; RI 0.75) with percentages of presence of the clades among equally parsimonious trees. In bold: *Kerberos langebadreae* gen. & sp. nov.

doi:10.1371/journal.pone.0135698.g016

Europe is roughly coeval with the disappearance of the large and hypercarnivorous proviverrines. The body mass range of the hyainailourines (46–98 kg), as noted above, sets them in a very different size class than either hyaenodontines and proviverrines (Fig 15). Consequently, hyaenodontines may have had a greater impact on the evolution of proviverrines than hyainailourines, which did not move into a previously occupied niche space (based on body mass).

The association of the mandible and skull allows detailed discussion of the masticatory musculature in *Kerberos* and a reconstruction of its feeding habits (see [94–98] for a description of the role and position of each masticatory muscle in carnivorous mammals). It should be noted that the temporal group musculature is more developed than the masseter group as usually observed in carnivorous mammals [9,94]. The anterior base of the coronoid process has a remarkably deep fossa with a significant anterior extension, producing a large insertion for the anterior fibers of the temporal muscle (pars orbitalis). Because this fossa is deeper than in "*Pterodon*", it would have allowed *Kerberos* a more powerful clenching action. As in "*Pterodon*", the squamosal and temporal areas, in which the deep masseter, zygomaticomandibularis and temporal muscle (pars temporalis) originate, are important. These three muscles are extensively developed; this observation correlates with the large masseteric fossa observed on the two dentaries. As indicated above, the anterior border of the choana is more anterior than the choana in *Pterodon dasyuroides*, providing a reduced surface area for the origin of the medial pterygoid muscle. The delicate angular process (relative to other hyainailourines) further supports the feeble contribution of the medial pterygoid to mandibular adduction. The enlargement of the pterygoid muscle in "*Pterodon*" was noted by Lange-Badré [9]. The important mastoid process, which is larger than in "*Pterodon*", suggests that the digastric muscle, an abductor and protruder of the mandible, was also powerful. However, the mastoid is also the

Table 5. Reconstructed locomotor styles and the position of the pes in several hyaenodonts.

Family	Species	Age	Locomotion	Pes	References
Sinopinae	*Prototomus martis*	Early Eocene	Terrestrial/scansorial	-	[34,113]
	Prototomus secundarius	Early Eocene	Scansorial	-	[113]
	Gazinocyon vulpeculus	Early Eocene	Terrestrial (incipient cursorial)	Digitigrade	[38]
	Sinopa major	Middle Eocene	Cursorial	Digitigrade	[55]
	Tritemnodon agilis	Middle Eocene	Terrestrial	Semi-digitigrade	[58]
Limnocyoninae	*Prolimnocyon atavus*	Early Eocene	Scansorial	Semi-digitigrade	[114]
	Limnocyon verus	Middle Eocene	Generalized	Plantigrade	[58,114]
	Thinocyon velox and *T. medius*	Middle Eocene	Semi-fossorial	Plantigrade	[115]
Hyainailourinae	*Kerberos langebadreae*	Middle Eocene	Terrestrial	Plantigrade	Present paper
	Hyainailouris sulzeri	Early Miocene	Terrestrial	Digitigrade	[4,33]
Arfiinae	*Arfia shoshoniensis*	Early Eocene	Terrestrial (incipient cursorial)	Plantigrade	[34,113]
Proviverrinae	*Lesmesodon**	Middle Eocene	Generalized	Plantigrade	[24]
	Cynohyaenodon cayluxi	Late Eocene	Generalized	Plantigrade	[21,24]
Hyaenodontinae	*Hyaenodon*	Oligocene	Cursorial	Digitigrade	[83]
Apterodontinae	*Apterodon langebadreae* and *Apterodon* indet.	Middle Eocene	Semi-aquatic	Plantigrade	[52]

**Lesmesodon* is only represented by juveniles [24].

doi:10.1371/journal.pone.0135698.t005

site of origin for much of the ventral cervical musculature and the separate sites of origin for these muscles are difficult to interpret (see [99–100] for a description of the muscle attachment sites in the mastoid region in mammals).

Although the cranial morphology of *Kerberos* is similar to that of "*Pterodon*", it has several more primitive features. The younger hyainailourine species expanded the role of the pterygoid muscles and of the masseter muscles, a shift evinced in the more developed angular process and reduced insertion for the temporal in "*Pterodon*" and younger species. Finally, the emphasis on the temporal musculature and digastric muscles in *Kerberos* indicates that this taxon would have been capable of powerful slicing and crushing near the molars, but the dentary was only capable of minimal lateral movements.

Savage [80] described a complete skull of *Megistotherium osteothlastes* (= *Hyainailouros bugtiensis*) and noticed that the skull is "moulded for maximum efficiency in feeding" (Savage, [80]: p.503). He particularly emphasized the importance of the temporal musculature that originated from the long, tall sagittal crest, a feature shared with *Kerberos*. *Hyainailouros bugtiensis* differs from *Kerberos* in having a shorter rostrum. Savage [80] interpreted the shortened face as important for stabilizing the roots of the canines during food capture. With a more extensive rostrum than *M. ostethlastes*, the canines may not have been used as actively in *Kerberos*.

The cervical vertebrae are unknown for *Kerberos*. However, several of these elements are known for Hyainailourinae and because the basicranial portion of the skull is similar for all known hyainailourines, the cervical vertebrae were likely similar across the subfamily. Savage [80] described the atlas of *Megistotherium* (= *Hyainailouros bugtiensis*) and Ginsburg [4] described two cervical vertebrae of *Hyainailouros sulzeri*. Savage [80] noted that the large size and broad transverse processes of the atlas imply significant extension of the head. The lateral cervical musculature associated with the large, transverse cervical processes indicates the animal was capable of strong lateral flexion of the head, which is particularly useful for restraining struggling prey. Concerning the cervical vertebrae of *H. sulzeri*, Ginsburg [4] noted the relatively short neural spine on the seventh cervical vertebra. This spine, which is taller and more robust in *Hyaenodon*, implies that *Hyainailouros* bore his head low, aligned with the thoracic vertebrae.

The secant molars M_{2-3} and M^{1-2} of Hyainailourinae exhibit little morphology that would aid in crushing (e.g., protocone and talonid on molars) and puncturing (e.g., metaconid), but the premolars are robust and display horizontal wear, which indicates they served a separate function from the slicing molars–the addition of a well-developed P^3 protocone in hyainailourines illustrates the crushing function hypothesized for the premolars. Based on this dental morphology Ginsburg [4,33] argued that "*Pterodon*" and *Hyainailouros* were bone crushers comparable to recent hyaenids, which also possess secant dentition. Stefen [101] noted that *Pterodon dasyuroides* and *Hyainailouros* possess only zigzag Hunter-Schreger-Bands (HSB), while limnocyonines *Prolimnocyon* (probably with the exception of *P. elisabethae*) and *Thinocyon*, and sinopines *Prototomus* and *Sinopa* display undulating HSB. A transitional state is found in *Apterodon* and some *Hyaenodon* and "*Pterodon*" species: they actually display a transition from undulating HSB at the enamel base through acute angled HSB to zigzag HSB at the tips of the teeth. She concluded that the development of zigzag HSB in the hyainailourines correlates with ossiphagy. The transition from undulating to zigzag HSB is notably found in most European *Hyaenodon* species–which also exhibits a pattern of wear–suggesting that bone crushing was also part of *Hyaenodon*´s diet [102].

Consequently, based on the taxon's robust premolars, the powerful cranial musculature and the phylogenetically bracketed presence of zigzag HSB, we hypothesize that *Kerberos* was capable of scavenging like its closest relatives. Extant mammal scavengers are also capable hunters, a mode of food acquisition likely partially utilized by *Kerberos* and hyainailourines more

generally. Werdelin [103] distinguishes two morphotypes among ossiphageous carnivores: the bone-crackers (e.g., spotted hyaenas), which break bones with their premolars, and bone-crushers (e.g., wolves) that break bones with their post-carnassial molars. Because *Kerberos* has extensive premolar wear, it may be considered a bone-cracker with a diet analogous to that of the extant hyenas (except the aardwolf).

The postcranial bones allow a partial reconstruction of the locomotion of *Kerberos*. The fibula exhibits interesting features related to locomotion. The fibula was not fused to the tibia. Furthermore, the fibula has a large distal head and large facet for contact with the calcaneus and a large proximal head in contact with the tibia. These features indicate a fibula capable of significant rotation for progression over rocky or uneven surfaces [104]. Among extant carnivores, such a rotatory fibula is known in bears and cats for instance and, among Hyaenodontidae, a rotary fibula is known in *Hyaenodon*, although *Hyaenodon* shows clear adaptations to cursoriality as well [83,105]. Other postcranial features indicate a plantigrade posture for *Kerberos* including: a transversally flattened caput astragali, a short astragalar neck, short and robust metatarsals and a short and robust calcaneus [106–107]. The sustentaculum tali has a plantar position, which is an osteological correlate for terrestrial locomotion [104]. The grooving of the astragalar trochlea is a well-known indicator for locomotion with cursors tending to have more deeply grooved astragali, while ambulators have more shallowly grooved astragali [108–109]. The relative depth of the astragalar trochlea also contains a strong phylogenetic signal [89]. The astragalar trochlea of *Kerberos* is shallow, but grooved. Its development suggests terrestrial locomotion, but clearly not in the cursorial posture indicated by the astragalar trochlea of *Hyaenodon* [105]. Because O'Leary et al. [110] recently proposed that scansorial features are basal for placental mammals, one must keep in mind that some of the features of *Kerberos* are potentially primitive, as they are found in many early Tertiary mammals (for instance see [111]).

To conclude, its postcranial anatomy suggests that *Kerberos* was a plantigrade, terrestrial mammal; we use the term "terrestrial" in the sense of Egi [91], referring to the following particular locomotion: "climbs rarely/never, scarcely running". This feature is important because it ecologically distinguishes *Kerberos* from two carnivoran groups that occupied the ossiphageous niches, the Borophaginae and Hyaenidae: these carnivorans are reconstructed to be cursorial [72,73]. In this regard, *Kerberos* appears more similar to the oxyaenid *Palaeonictis* because the latter displays dental ossiphageous adaptations [62] and a terrestrial locomotion [23,112].

The locomotor styles of hyaenodonts are poorly known: the postcranial morphology of only a few taxa has been studied, and most of these taxa are from North America (Table 5). Interestingly, a wide range of locomotion has been hypothesized: the limnocyonine *Thinocyon* is a probable semi-fossorial form, *Hyaenodon* is cursorial, while *Apterodon* shows semi-aquatic adaptations [52,83,115]. This lack of knowledge and the apparent diversity of locomotor adaptations among hyaenodonts prevent a rigorous reconstruction of the ancestral locomotor state for this group. However, based on the postcranial elements found in North America we are able to posit a possible primitive locomotor pattern for hyaenodonts. The oldest North American hyaenodonts have adaptations that would have allowed them to move in both arboreal and terrestrial environments. Rose ([113]: p.166) even noted that the "Wasatchian hyaenodontid [an]s, probably [were] reasonably facile in the trees as well as on the ground." Among North American hyaenodonts, the Sinopinae and Arfiinae differ from the Limnocyoninae in being more terrestrial. For instance, *Arfia* displays a mosaic combination of cursorial forelimb adaptations and scansorial hindlimb adaptations [34]. The ability to exploit both the trees and the ground was probably the ancestral condition of the hyaenodonts. Finally, the presence of plantigrady in numerous, primitive hyaenodonts allows us to hypothesize a plantigrade foot at the origin of hyaenodonts.

The sole hyainailourine for which the locomotion has been studied is *Hyainailouros sulzeri*. Ginsburg [4,33] reconstructed *Hyainailouros* not as a fast runner, but as a capable jumper. He also provided arguments for a digitigrade stance. According to our analysis, *Hyainailouros* is more derived than *Kerberos* and the postcranial morphology of the new taxon is consistent with the retention of an archaic locomotion.

In conclusion, *Kerberos* was a heavily built hyaenodont, with a plantigrade posture that predominantly moved across terrestrial substrates. Because of its size and the absence of obvious adaptations to cursoriality, *Kerberos* was likely not a fast runner but rather complemented active predation with scavenging. Carbone et al. [88,116] demonstrated that extant carnivorous mammals that weigh above 21.5–25 kg generally hunt prey as large or larger than themselves. Because *K. langebadreae* exceeds this size bracket, it probably focused on large prey such as large artiodactyls (e.g., *Choeropotamus*) and perissodactyls (e.g., *Lophiodon* and *Palaeotherium*). Due to its large body mass, *Kerberos langebadreae* was probably an important, apex predator among the European faunas of the Bartonian. However, as noted by several authors [117–118], the reconstruction of the ecomorphology of extinct carnivorous mammals is difficult especially when a taxon like *Kerberos* displays unique character combination, which are not any analogous to those found in extant carnivorans: *Kerberos* with features similar both to *Palaeonictis* and several living hyenas, clearly illustrates this issue.

Phylogeny of the Hyainailourinae

As indicated above, Hyainailourinae is characterized by a secant dentition as Hyaenodontinae. Polly [38] demonstrated that the two groups are not closely related and convergently acquired similar shearing dentitions. He provided cranial and dental characters for differentiating the two hypercarnivorous groups. He included *Pterodon*, *Hyainailouros*, *Hemipsalodon*, *Sivapterodon*, and *Megistotherium* among hyainailourines. Holroyd [37] added the genus *Akhnatenavus* and *Metapterodon*. Peigné et al. [119] removed *Dissopsalis* and *Anasinopa* from the "Afro-Asian proviverrines" of Egi et al. [60], and referred them to Hyainailourinae as well. *Dissopsalis* and *Anasinopa* differ from *Pterodon* and its closely affiliated taxa by the presence of a wide talonid and the retention of the metaconid. Lewis & Morlo [70] used this work as a basis for the establishment of a list of African hyainailourines. Hyainailourinae in Morlo et al. [53] further added African "*Sinopa*" and *Metasinopa* to the group.

The recent study of Solé et al. [6], based on an analysis of the oldest hyainailourines *Furodon* and *Parvavorodon*, restricted Hyainailourinae to its original definition [38]. The authors conserved the following African genera: *Pterodon*, *Hyainailouros*, *Isohyaenodon*, *Leakitherium*, *Sivapterodon*, *Akhnatenavus*, *Furodon*, and *Parvavorodon*. The African "*Sinopa*", *Metasinopa*, *Dissopsalis* and *Anasinopa* were referred to Teratodontinae. Moreover, Solé et al. [6] found a close relationship between Hyainailourinae and Koholiinae. Our present study of hyaenodont crania allows Apterodoninae and Hyainailourinae to be grouped into Hyainailouridae and the separation of Hyainailouridae from Teratodontinae.

In an effort to better understand the relationships within Hyainailourinae, we performed phylogenetic analyses of the group including closely related taxa. A study of hyainailourines was partially undertaken by Holroyd in her PhD thesis [37]. However, she included several taxa that have been excluded from Hyainailourinae in subsequent work (e.g., *Matthodon*, *Paenoxyaenoides*, and *Oxyaenoides*, which are now referred to Proviverrinae, and *Metapterodon*, which is referred to Koholiinae), and she restricted her study to the Eocene and Early Oligocene hyainailourines.

Our study includes almost all hyainailourines that are known by a complete lower or upper dentition. We did not include either *Parvavorodon* or *Pterodon* sp. [69], because the former is

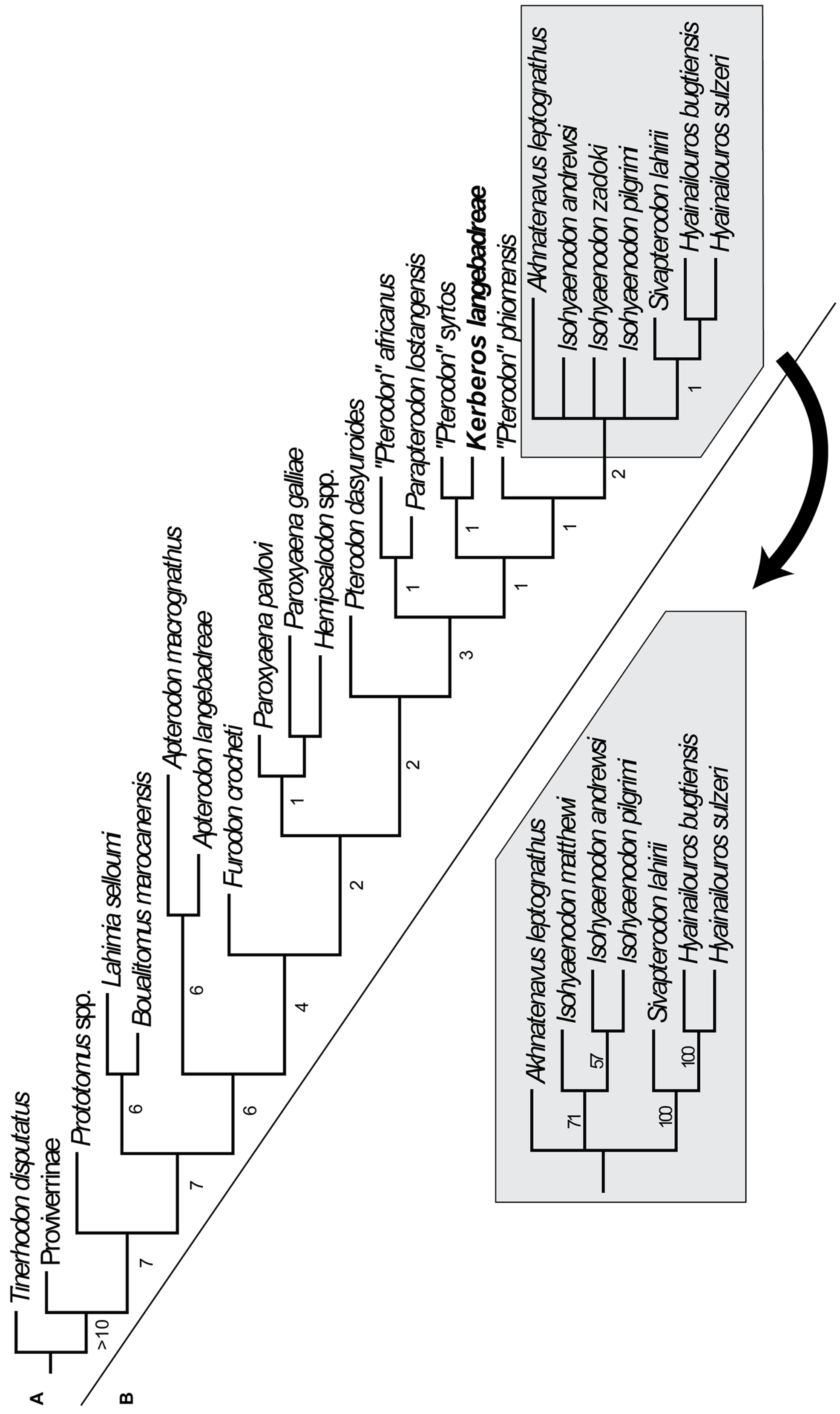
A
B
Tinerhodon disputatus
Proviverrinae
Prototomus spp.
Lahimia selloumi
Boualitomus marocanensis
Apterodon macrognathus
Apterodon langebadreae
Furodon crocheti
Paroxyaena pavlovi
Paroxyaena galliae
Hemipsalodon spp.
Pterodon dasyuroides
"Pterodon" africanus
Parapterodon lostangensis
"Pterodon" syrtos
Kerberos langebadreae
"Pterodon" phiomensis
Akhnatenavus leptognathus
Isohyaenodon andrewsi
Isohyaenodon zadoki
Isohyaenodon pilgrimi
Sivapterodon lahirii
Hyainailouros bugtiensis
Hyainailouros sulzeri
Isohyaenodon matthewi

Fig 17. Consensuses of the second phylogenetic analysis of the hyainailourines with *Leakitherium* excluded. A: strict consensus (120 steps long; CI 0.59; RI 0.75) of the second phylogenetic analysis of the hyainailourines which includes body size character with indications of the Bremer support values. B: relationships among several hyainailourines in the majority rule consensus (117 steps long; CI 0.60; RI 0.76) with percentages of presence of the clades among equally parsimonious trees. In bold: *Kerberos langebadreae* gen. & sp. nov.

doi:10.1371/journal.pone.0135698.g017

only known by two isolated teeth, and because the maxillary fragment of the latter is much abraded. We also included the apterodontines *Apterodon langebadreae* and *A*. *macrognathus*, as well as the koholiines *Lahimia* and *Boualitomus*, because the two subfamilies share many characters with Hyainailourinae, which can potentially help polarizing character transformations within the in-group. The purpose of this study is not to test all hyaenodont relationships. Several taxa, notably species of Hyaenodontidae, were not included and will be considered in future evaluations of the phylogenetic relationships within Hyaenodonta.

Three distinct analyses have been performed (see Material and methods section). The first analysis (all the taxa and characters included) yielded 201 equally parsimonious trees, with a tree length of 117 steps, consistency index (CI) of 0.60 and retention index (RI) of 0.77. The strict consensus tree is 135 steps long (CI = 0.52 and RI = 0.68) (Fig 16A). The majority rule consensus is 120 steps long (CI = 0.59 and = RI 0.75; Fig 16B). The Hyainailourinae includes the genera *Paroxyaena* and *Hemipsalodon*. In this analysis the genus *Pterodon* is not monophyletic and will be closely examined in a future work. The second analysis (*Leakitherium* deleted) yielded 7 equally parsimonious trees, with a tree length of 116 steps, consistency index (CI) of 0.61 and retention index (RI) of 0.77. The strict consensus tree is 120 steps long (CI = 0.59 and RI = 0.75) (Fig 17A). The majority rule consensus is 117 steps long (CI = 0.60 and RI = 0.76; Fig 17B). The third analysis (*Leakitherium* deleted and character related to body mass excluded) resulted in 8 equally parsimonious trees (L = 106; CI = 0.63; RI = 0.78; the strict consensus has a length of 111 steps for a CI of 0.60 and a RI of 0.76) (Fig 18). Fig 19 depicts the consensus tree of the third analysis with stratigraphical and geographical information.

The proposed topology does not show temporal incoherence. However, the topology implies a significant ghost lineage for *Pterodon dasyuroides*. As demonstrated by Holroyd [69], the hyainailourines experienced a significant diversification during the Late Eocene. This diversification should be interpreted cautiously as there are significant gaps in the fossil record of hyainailourines, particularly in the early Paleogene record of Africa. Interestingly, the two Neogene groups–the small hyainailourines represented by *Isohyaenodon* and the very large *Hyainailouros* and *Sivapterodon*–are rooted in Late Eocene taxa.

The consensus of the third analysis differs from the consensus trees of the second analysis by the absence of close relationship between the Apterodontinae and Hyainailourinae although some relationships remain unresolved. Moreover, the Bremer support values are slightly higher. In the second analysis (Fig 17), the clade formed by the Apterodontinae and the Hyainailourinae is supported by the presence of large premolar diastemata [35(2)], an increase of size [1(3)], reduction of the metaconid [37(2) Fast Optimization (= FO) & Slow Optimization (= SO)], a weak paraconule crista [16(1) (SO)] and a paracone greater than the metacone [20 (1) SO]. The close relationships between these two subfamilies was hypothesized by Grohé et al. [52] and Rana et al. [61]–together with African Teratodontinae in the latter study–but not by Solé et al. [6]. Our expanded taxonomic sampling allows more complete examination of this relationship. Moreover, as discussed above, the two subfamilies share numerous postcranial and cranial features that would likely lend support to the Apterodontinae+Hyainailourinae clade defined as Hyainailouridae.

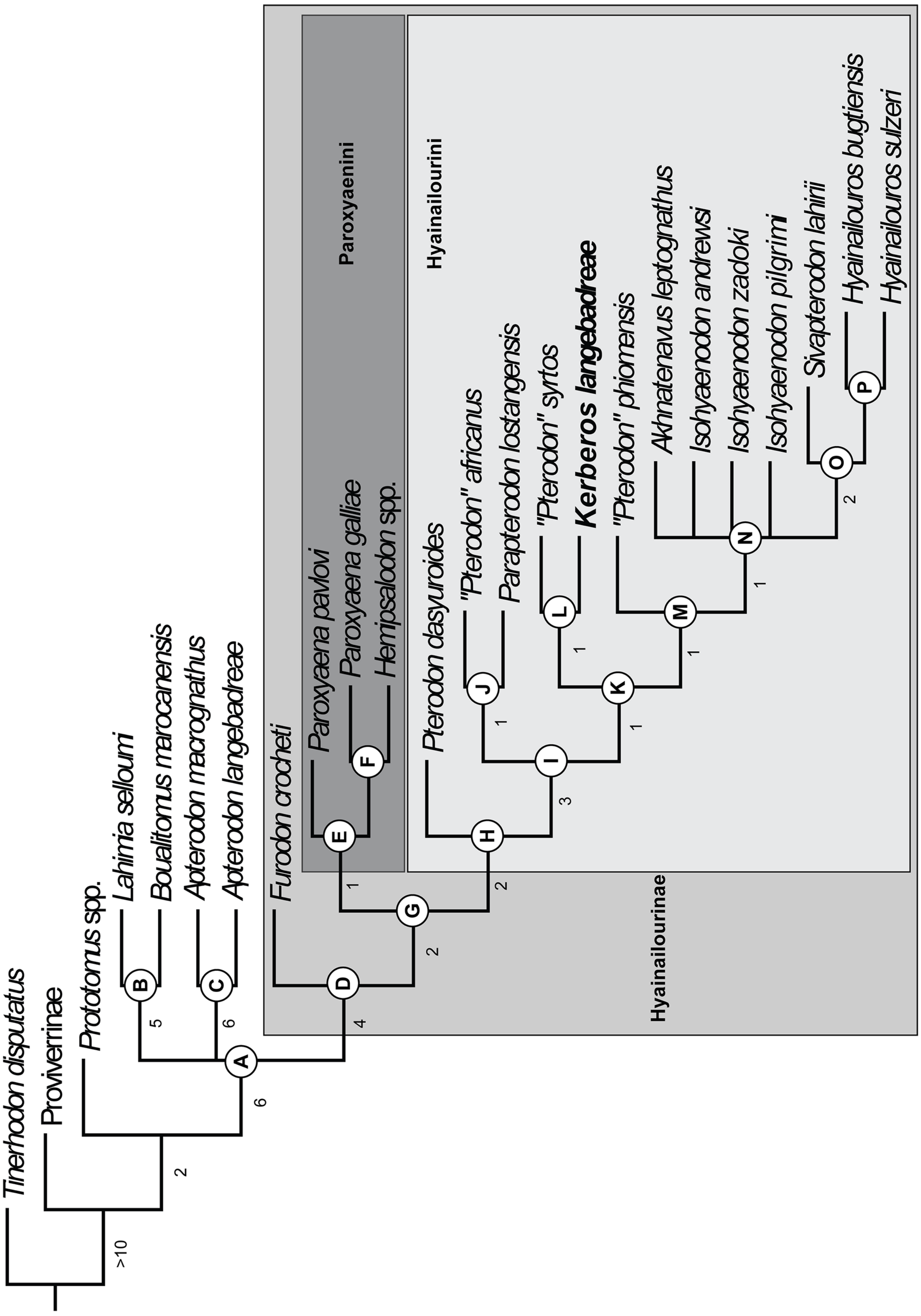
Tinerhodon disputatus
Proviverrinae
Prototomus spp.
Lahimia selloumi
Boualitomus marocanensis
Apterodon macrognathus
Apterodon langebadreae
Furodon crocheti
Paroxyaena pavlovi
Paroxyaena galliae
Hemipsalodon spp.
Pterodon dasyuroides
"Pterodon" africanus
Parapterodon lostangensis
"Pterodon" syrtos
Kerberos langebadreae
"Pterodon" phiomensis
Akhnatenavus leptognathus
Isohyaenodon andrewsi
Isohyaenodon zadoki
Isohyaenodon pilgrimi
Sivapterodon lahirii
Hyainailouros bugtiensis
Hyainailouros sulzeri
Paroxyaenini
Hyainailourini
Hyainailourinae
A
B
C
D
E
F
G
H
I
J
K
L
M
N
O
P
>10
2
6
5
6
4
2
1
2
3
1
1
1
1
1
2

Fig 18. Strict consensus of the third phylogenetic analysis of the hyainailourines with *Leakitherium* and body mass character excluded. 111 steps long; CI 0.60; RI 0.76; with indications of the Bremer support values. In bold: *Kerberos langebadreae* gen. & sp. nov.

doi:10.1371/journal.pone.0135698.g018

We describe in the following paragraphs the consensus tree of the third analysis because the analysis was not disturbed by *Leakitherium* and not influenced by the body masses (Fig 18). The node A is supported by a weak and short paraconule crista on the molar [16(1)], the reduction of the P_2 and P_3 relative to P_4 [29(1)] and the reduction of the metaconid [37(1)]. The Koholiinae (node B) are supported by the loss of the P_1 [28(2)] and a molar series longer than the premolar series [36(1)]. The Apterodontinae (node C) are grouped based on the following features: reduction of the metastyle on P^4 [9(1)], completely separated paracone and metacone of equal height [19(0), 20(1)], reduced parastyle on molars [22(1)], absence of ectoflexus [25 (0)], reduced distal cuspulid on P_3 [31(1)], talonid as wide as the trigonid [44(0) FO], and P^3 metastyle present [6(1) SO]. *Apterodon macrognathus* differs from the earlier *A*. *langebadreae* by a single-rooted P^1 [3(1)], the reduction of the metaconule [17(1)], the absence of metaconid and entoconid on molars [37(2), 40(2)], and a postprotocristid directed distally [48(1)]. Grohé et al. [52] hypothesized that *Apterodon* could be a semi-aquatic hyaenodont based on postcranial features. The peculiar morphology of apterodontine dentitions–homogenized premolars and simplified molars–could be an adaptation for a piscivorous diet. This homogenized, simplified dentition is reminiscent of pinniped dentition.

The Hyainailourinae (node D) are supported by a small, mesially located protocone on the molars [24(1), 26(1)], P_4 tall and long [33(2)], a protoconid as long as the paraconid [47(0)], a P^3 with large lingual cingulum [5(1) FO], P^4 mesial root larger than the distal one [14(1) FO], a long metastyle on molars [18(1) FO], large anterior keels [42(1) FO] and higher paraconid on molars [46(1) FO]. The oldest hyainailourine presently known–*Furodon* from late Early or early Middle Eocene of Algeria–is the sister-group of all other hyainailourines. *Parvavorodon* has not been included in the phylogenetic analysis (see above) and has been placed at the node C (Fig 19) with an unresolved relationship with other hyainailourines. This position for *Furodon* contradicts the hypothesis of Solé et al. [6] that *Furodon* could be closely related to *Akhnatenavus*.

Numerous features support node E: the absence of the metaconule on the molars [17(1)], the length of the molars equivalent to that of the premolars [36(2)], the loss of the metaconid [37(2)], the reduced talonid on M_3 [49(1)], the elongation of the metastyle on molars [18(2) FO], the distally directed postprotocristid [48(1) FO], the reduction of the parastyle on P^4 [10 (2) SO], and the reduction of the M^3 [27(1) SO]. Some of these features are found in hyaenodontines. However, as shown by Polly [38], these features have been acquired convergently in hyaenodontines and hyainailourines.

Node F (= Paroxyaenini) groups the two species of *Paroxyaena* and the North American genus *Hemipsalodon*. The synapomorphies at this node are the paracone and metacone of equal height [20(1)], the strong protocone on P^3 and P^4 [5(2) FO, 7(0) FO, 13(0) FO], the presence of a metastyle on P^3 [6(0) FO], the reduced shearing surface of the postparacrista on P^4 [12(1) FO], the small anterior keel on the paraconid [42(0) FO], the talonid as wide as the trigonid [44(0) FO], and a paraconid lower than in other hyainailourines [46(0) FO]. It is interesting to note that these two genera have a basal position among hyainailourines in our topology. This early origination agrees with the pleisiomorphic features retained in the group such as the separated paracone and metacone on molars. Surprisingly, *Paroxyaena pavlovi* is closer to *Hemipsalodon* than to *Paroxyaena galliae* (node G); however, it should be noted that *Paroxyaena galliae* is currently only represented by a juvenile specimen [8], and its permanent dentition is not completely known.

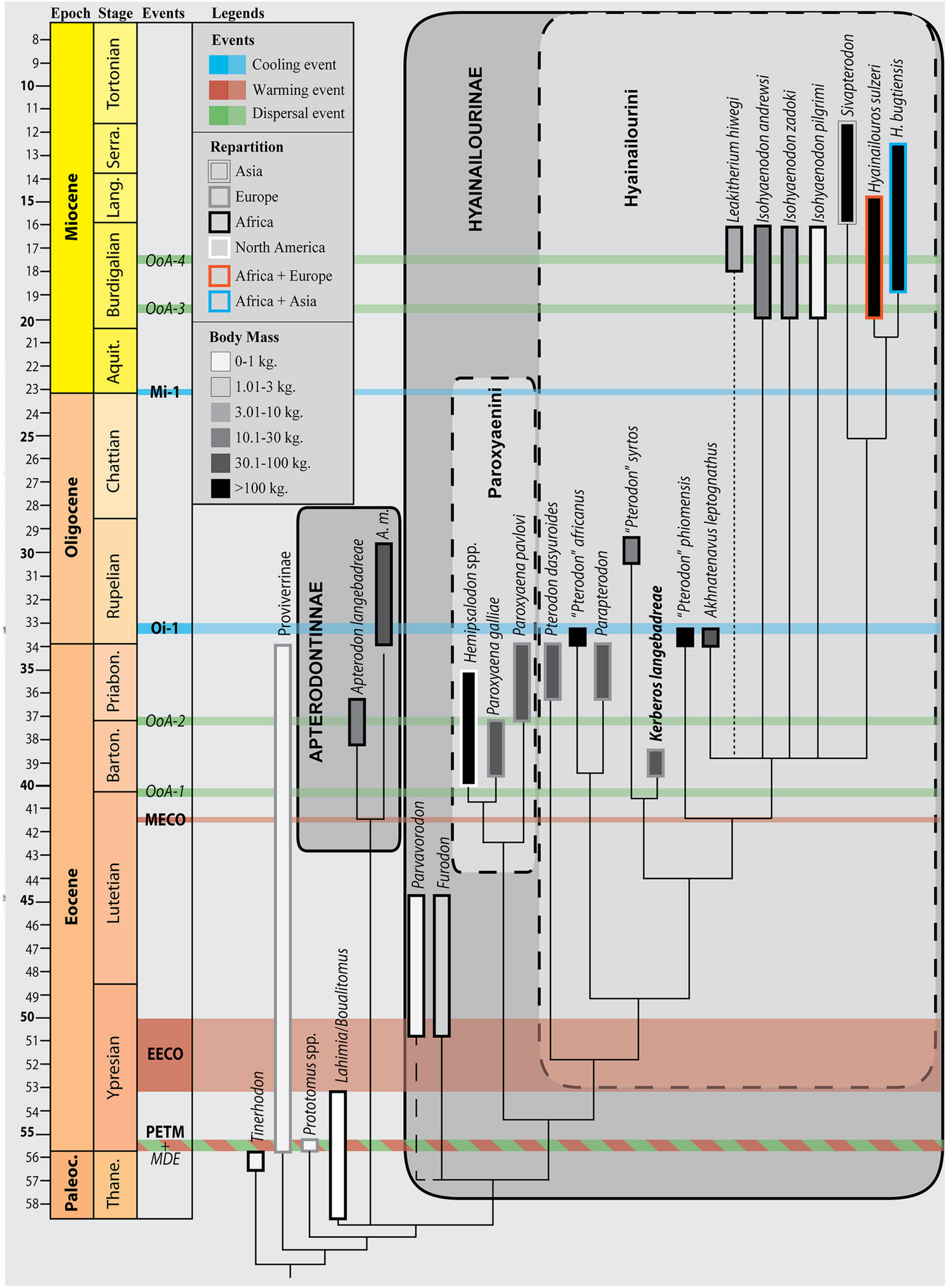
Epoch
Stage
Events
Legends
Events
Cooling event
Warming event
Dispersal event
Repartition
Asia
Europe
Africa
North America
Africa + Europe
Africa + Asia
Body Mass
0-1 kg.
1.01-3 kg.
3.01-10 kg.
10.1-30 kg.
30.1-100 kg.
>100 kg.
Miocene
Oligocene
Eocene
Paleoc.
Tortonian
Serra.
Lang.
Burdigalian
Aquit.
Chattian
Rupelian
Priabon.
Barton.
Lutetian
Ypresian
Thane.
OoA-4
OoA-3
Mi-1
Oi-1
OoA-2
OoA-1
MECO
EECO
PETM
+
MDE
HYAINAILOURINAE
Hyainailourini
Paroxyaenini
APTERODONTINNAE
Tinerhodon
Proviverrinae
Prototomus spp.
Lahimia/Boualitomus
Apterodon langebadreae
A. m.
Parvavorodon
Furodon
Hemipsalodon spp.
Paroxyaena galliae
Paroxyaena pavlovi
Pterodon dasyuroides
"Pterodon" africanus
Parapterodon
"Pterodon" syrtos
Kerberos langebadreae
"Pterodon" phiomensis
Akhnatenavus leptognathus
Leakitherium hiwegi
Isohyaenodon andrewsi
Isohyaenodon zadoki
Isohyaenodon pilgrimi
Sivapterodon
Hyainailouros sulzeri
H. bugtiensis

Fig 19. Phylogeny of the hyainailourines with stratigraphic and climatic indications based on third phylogenetic analysis with *Leakitherium* deleted and body mass character excluded. Abbreviations: Paleoc., Paleocene; Thane., Thanetian; Barton., Bartonian; Priabon., Priabonian; Aquit., Aquitanian; Lang., Langhian; Serra., Serravallian; PETM, Paleocene-Eocene Thermal Maximum; MDE, Mammal Dispersal Event; EECO, Early Eocene Climatic Optimum; MECO, Middle Eocene Climatic Optimum; Oi-1, Oligocene Oi-1 event; Mi-1, Miocene Mi-1 event; OoA, "Out of Africa" event; *A.*, *Apterodon*; *A. m.*, *Apterodon macrognathus*; *H.*, *Hyainailouros*.

doi:10.1371/journal.pone.0135698.g019

The relationship between *Paroxyaena* and *Hemipsalodon* allows the origin of the North American genus to be reconstructed. Contrary to Mellett [65], Gustafson [120] considered a close relationship between *Pterodon* and *Hemipsalodon* possible because of similarities in the basicranial regions of both genera. We follow the opinion of Gustafson [120] in considering *Hemipsalodon* as a hyainailourine. As shown by the phylogenetic analysis, several dental features such as the separation of the paracone and metacone of equal height and the development of a large protocone on P^3 support a sister-relationship with *Paroxyaena*. Moreover, we can add the following features: the presence of three upper incisors, presence of a lingual cingulum on the premolars and molars, the non-reduction of the M^3 (compared to other hyainailourines, except *Kerberos*), the shortening of the rostral part of the skull, and the presence of a depression on the lateral part of the maxilla.

To emphasize the peculiar morphology of *Paroxyaena* among hyainailourines, Lavrov [8] recognized the tribe Paroxyaenini. The Paroxyaenini are characterized by the fusion of the orbital fissure and the foramen rotundum. Due to this fusion, the Paroxyaenini have only three foramina in the posterior part of the orbital region. Gustafson [120] noted the presence of three foramina instead of four in *Hemipsalodon*, but he misidentified them; thus, from anterior to posterior, *Hemipsalodon* has an ethmoid foramen, an optic foramen, and a fused fissura orbitalis and foramen rotundum. As no feature excludes *Hemipsalodon* from Paroxyaenini, we include this genus in the tribe. The presence of *Hemipsalodon* among Paroxyaenini has important biogeographic implications as this genus is the only hyainailourine recorded in North America (see below).

Node H is supported by a weak lingual cingulum on the upper premolars [4(0)], weak mesiolabial cingulum on the upper molars [15(1)], fused metacone and paracone [19(2)], small talonid on lower molars [43(1), 45(1)], loss of the paraconule [21(1) FO], and reduction of the talonid cusps [40(2) FO, 41(1) FO]. *Pterodon dasyuroides* is characterized by the reduction of the number of upper incisors [2(1)] and of the fusion of the symphyseal region [50(1)]–while the latter feature is typical of this species, the first character is also known in *Hyainailouros*.

Node I is notably supported by the presence of three roots on P^3 [8(0)], the reduction of the M_1 talonid [39(1)], and the reduction of the P^1 [3(1) FO]. *Parapterodon* and "*Pterodon*" *africanus* (node J) are grouped based on the loss of M^3 [27(2)]. Node K is supported by the absence of the parastyle on the molars [22(1)], and the increase of the length of the premolars relative to the molars [36(0) FO].

Node L is supported by a mesial root larger than the distal root on P^4 [14(0)] and a double-rooted P^1 [3(0) FO]. It groups "*Pterodon*" *syrtos* and the new genus *Kerberos*. The former differs from *Kerberos* by the completely fused paracone and metacone [19(3)SO]. The notable features that support node M are the absence of a talonid on M_3 [49(2)], a distally directed postprotocristid [48(1) FO & SO], the loss of the mesiolabial cingulum on the upper premolars and molars [15(2) FO], and reduction of the talonid cusp [40(2) SO, 41(1)]. Node N, which comprises Eocene *Akhnatenavus* and the Miocene hyainailourines *Isohyaenodon*, *Sivapterodon* and *Hyainailouros*, is notably supported by a tall and short P_4 [33(1)], the strong reduction of the talonid on M_2 [45(2)], and the presence of very short diastemata between the premolars [35(1) FO]. The relationships within this clade however are poorly resolved. The evolution of the hyainailourines during the later Oligocene is presently poorly known (Fig 19); only several

fragmentary fossils of hyainailourines have been reported from this period (see for instance Rasmussen & Gutierrez [121]). Consequently, this lack of fossils could explain the irresolution of the relationships within this clade. This node is comprised of two distinct size groups: node O corresponds to the small Neogene hyainailourines (*Isohyaenodon*), while node P includes the largest hyainailourines ever recorded (*Sivapterodon* and *Hyainailouros*). Consequently, the separation between these two ecological groups occurred during the Oligocene or earlier.

Akhnatenavus is distinguished by the absence of a distal cingulid on P_3 [31(1)], reduction of the talonid on P_4 [34(1)], presence of large diastemata between the premolars [35(0)], and a less reduced talonid on M_2 [45(1)]. *Isohyaenodon* is not monophyletic in the strict consensus tree of the third analysis, but it was monophyletic in the majority rule consensuses of the first and second analyses. However, the relationships within the *Isohyaenodon* genus do differ between the first and second analyses. *Isohyaenodon* is a good example of the trend among hyainailourines towards a simplification of the molars. *Isohyaenodon andrewsi*–the largest species of the genus–is characterized by the presence of a talonid on M_1 [39(0)].

Node O is supported by a distally elongated postprotocrista [48(2)], the loss of the distal cuspulid on P_3 [31(1) FO], a low and broad P_4 [33(0) FO], molar series longer than the premolar series [36(1) FO], and a very reduced M_1 [38(1) FO]. The two species of *Hyainailouros* form a clade (node P), which supports the monophyly of the genus and the distinctive diagnosis of *Sivapterodon* (see Pilgrim [47] and Ginsburg [4]); this group is however only supported by a low and broad P_4 [33(0) SO] and a very reduced M_1 [38(1) SO].

Evolution of size among hyainailourines

The large size of *Kerberos* was unexpected: it is larger than any other contemporaneous European hyaenodont (Fig 15). With the inclusion of North American *Hemipsalodon* in Hyainailourinae, there were at least two large-bodied Hyainailourines in Laurasia during the Bartonian. The large body size, exemplified by Bartonian *Kerberos* and *Hemipsalodon*, is a common evolutionary tendency in hyainailourines (Fig 19). Hyainailourinae includes one of the largest carnivorous mammals ever known: *Megistotherium* (= *Hyainailourus bugtiensis*). The skull of *Megistotherium* is about 66 cm long—nearly twice as long as that of *Kerberos*—and the animal may have weighed as much as 800 kg [80].

In his description of *Megistotherium*, Savage [80] observed that the face of hyainailourines is relatively long when compared to that of carnivorans and Hyaenodontidae. He proposed two hypotheses for the conservation of a long mandible: (1) it is non-adaptive and corresponds to a conserved genetic trait, or (2) it is selective and related to the size of preferred prey (e.g., anthracotheres, pigs, rhinoceroses, mastodonts and deinotheres). The second hypothesis is supported by the abundance of proboscideans in *Megistotherium*'s fauna. Savage [80] estimated that *Megistotherium*–due to the length of its jaw–may have had 30 cm of clearance between the tips of the canines, which would allow the animal encircle and bite a proboscidean limb. Rasmussen et al. [122] hypothesized that large hyainailourines, which mainly radiated in Africa, evolved originally as specialized predators or scavengers of the massive herbivores endemic to Africa, such as embrithopods and proboscideans. Ginsburg [4] supported this hypothesis and noted that *Hyainailouros* appeared in Europe together with the proboscideans (Proboscidean datum; see [123]). Large body size is an ancient feature among proboscideans with the oldest representatives of group already relatively large [124]. *Numidotherium*, which is known from the latest Early Eocene of Algeria, was one of the largest terrestrial mammals of its time, and initiated an impressive size increase among proboscideans [125]. Following Rasmussen et al. [122], we therefore hypothesize that the large size of hyainailourines may result from co-evolution with endemic African herbivores such as proboscideans, though this hypothesis

must be more rigorously tested. However, as demonstrated at a broader mammalian scale, large mammals tend to have longer faces than closely related but smaller ones [126]. Therefore, another hypothesis would be that the long face of *Megistotherium* is related to the constraints of body size.

The large body size of the hyainailourines, exemplified by *Kerberos* and by *Hemipsalodon*, appears early in the history of the subfamily. The large size of the Miocene *Hyainailouros* and *Sivapterodon* could result from either conservation or convergence on this trait. Because *Hyainailouros* and *Sivapterodon* are close to the small *Isohyaenodon* in the phylogenetic analyses we performed, it is possible that gigantism evolved at least twice in hyainailourines.

In the same faunas occupied by the gigantic *Hyainailouros* species, there were also smaller hyainailourines. The smaller hyainailourines are known from the Late Eocene (*Akhnatenavus*) to Middle Miocene (*Isohyaenodon*) (Fig 19). The largest one (*I. andrewsi*) only weighed around 14 kg. Holroyd [69] hypothesized that these differences in sized among hyainailourines are suggestive of the exploitation of different ecological niches. Based on Carbone et al. [88,116], these smaller taxa are below the threshold for hunting prey equal to or larger than their body size. Conversely, they likely exploited prey smaller than them. Unfortunately, the skull and postcranium are unknown for these small hyainailourines, which prevents a clear evaluation of their ecology (i.e. locomotion, choice of prey). The presence of these small-bodied hyainailourines suggests that the hyainailourine bauplan was not specifically adapted for large-bodied predation.

The second cladistic analysis allows discussing the evolution of the body mass among Hyainailourinae in a phylogenetic context. The acquisition of a large body mass (between 31 and 100 kg) [1(3)] is reconstructed for the clade that comprises the hyainailourines and apterodontines. There are convergent secondary return to a smaller body size in *Apterodon langebadreae*, *Akhnatenavus*, *Isohyaenodon andrewsi* [1(2)] (between 11 and 31 kg), *Furodon crocheti*, *Isohyaenodon zadoki* [1(1)] (between 1 and 11 kg) and *Isohyaenodon pilgrim* [1(0)] (below 1 kg). In contrast, independent acquisitions of a large body size state are reconstructed in *Hemipsalodon*, *"Pterodon" phiomensis*, *"Pterodon" africanus* and in the clade formed by *Sivapterodon* and *Hyainailouros* [1(4)] (> 100 kg).

The hypothesis of several reductions of the body mass among hyainailourines (and apterodontines) is poorly supported by the analyses of body size evolution among carnivorous mammals. Van Valkenburgh et al. [127] tested the Cope's rule in extinct canids (borophagines and hesperocyonines) and noticed a clear evolutionary trend toward larger body size in these groups. They argued that energetic constraints and pervasive selection for larger size in carnivorous mammals (e.g., killing and feeding on large preys) also lead to dietary specialization (hypercarnivory). These factors increase vulnerability to extinction, explaining why large hypercarnivorous mammals appear to have been limited in their temporal appearance relative to smaller and more conservative species: a greater vulnerability to extinction ultimately results from evolution towards a larger body size. Based on this remarkable study, the possibility that large sizes evolved at least twice in hyainailourines is more reasonable than several cases of convergent reductions of the body mass. Finally, it is worth reminding that the oldest and most primitive hyainailourine and apterodontines are only of small sizes [6,52].

Paleobiogeographic implications

Based on the discovery of *Parvavorodon* and *Furodon*, Solé et al. [6] hypothesized that hyainailourines originated in Africa during the Early Eocene (*contra* Egi et al. [128]). The presence of a new hyainailourine in the Bartonian of Europe has implications for the terrestrial connection between Europe and Africa during the Eocene. The earliest appearance of the Hyainailourinae

in Europe was *Paroxyaena galliae*, which is known from the MP16, MP17a and MP17b reference-levels [9,27,85] (Fig 1). The presence of *Kerberos* is consistent with the appearance of Hyainailouridae in Europe at MP 16 (Fig 1). *Pterodon dasyuroides* is recorded in the MP18 reference-level but is unknown after MP20 reference-level. The stratigraphic position of *Parapterodon* is also presently uncertain but is probably contemporaneous with *Pterodon* [9] (Fig 1). As noted by Crochet et al. [85], *Paroxyaena* and *Pterodon* were not present in Europe at the same time. The description *Paroxyaena pavlovi* by Lavrov [8] could contradict this statement, but the stratigraphic position of this species is still uncertain.

These stratigraphic partitions raise the question of the dispersal of Hyainailourinae into Europe and the subfamily's diversification on the European continent. Did the Hyainailourinae disperse once or several times from Africa to Europe? Did the Hyainailourinae diversify in Europe?

The primitive features of *Kerberos* (e.g., presence of three upper incisors, large P_1 and P^1) conforms with an older age for *Kerberos* compared with the more derived features of "*Pterodon*" and *Parapterodon* (Fig 1). *Paroxyaena*, which is also recorded in the Bartonian of Europe, shares with *Kerberos* an unreduced number of upper incisors. However, the two genera are clearly different taxa (notably in the reduction of the P_1 and P^1 in *Paroxyaena* and the separation of the paracone and metacone). *Paroxyaena* clearly differs from *Kerberos*, "*Pterodon*", and *Parapterodon* and represents a distinct lineage (see the phylogenetic analysis). This supports a diversification of the Hyainailourinae during the Bartonian or earlier. However, this diversification is poorly understood due to the lack of fossils, and may have occurred either in Africa or in Europe.

As for the relationships among *Kerberos*, *Parapterodon* and "*Pterodon*", the new genus is distinguished by its larger size, more derived molars (e.g., parastylar reduced) and a more developed protocone on P^3. Because of these differences, *Kerberos* does not seem to be ancestral to *Pterodon dasyuroides* or *Parapterodon*. Consequently, it appears that the Hyainailourini did not diversify and radiate in Europe, but rather dispersed at least twice into Europe. The Paroxyaenini seem to have diversified in Laurasia, but this needs further scrutiny.

The hyainailourines are unknown in Asia until the Miocene. The species *Oriensterodon dahkoensis* described by Egi et al. [128] from late Middle Eocene of Asia is now considered part of Hyaenodontidae and Indohyaenodontinae rather than to Hyainailouridae [70]. This absence implies that the Bartonian hyainailourines may have dispersed directly from Africa to Europe *via* the Iberian or Apulian route.

The history of trans-Tethyan Paleogene mammal dispersals is characterized at least by four dispersal phases: during the Early Thanetian, and by the Thanetian/Ypresian, Bartonian/Priabonian, and Priabonian/Rupelian transitions. Two other dispersals between Africa and Laurasia (by the Ypresian/Lutetian and by the Lutetian/Bartonian transitions) have less support [129].

Here we refer to the dispersals of hyainailourines between Africa and Europe as the "Out of Africa event" (= OoA).

Gheerbrant & Rage [129] considered the Lutetian/Bartonian Dispersal Phase as doubtful and minor, but it could explain the dispersal of amphipithecids from Africa to Laurasia. The presence of *Kerberos* and *Paroxyaena* in the earliest part of Bartonian (Fig 19) further substantiates this dispersal phase hypothesis. The Lutetian/Bartonian Dispersal Phase corresponds to the first migration of hyainailourines to Laurasia (OoA-1 on Figs 19 and 20). The appearance of the hyainailourines in Europe is related to a modification of the European mammal fauna: the second intra-Eocene mammal turnover of Franzen [130]. This turnover seems to be related to dispersals between Central European Island and Iberian Peninsula [130–131]. Based on this

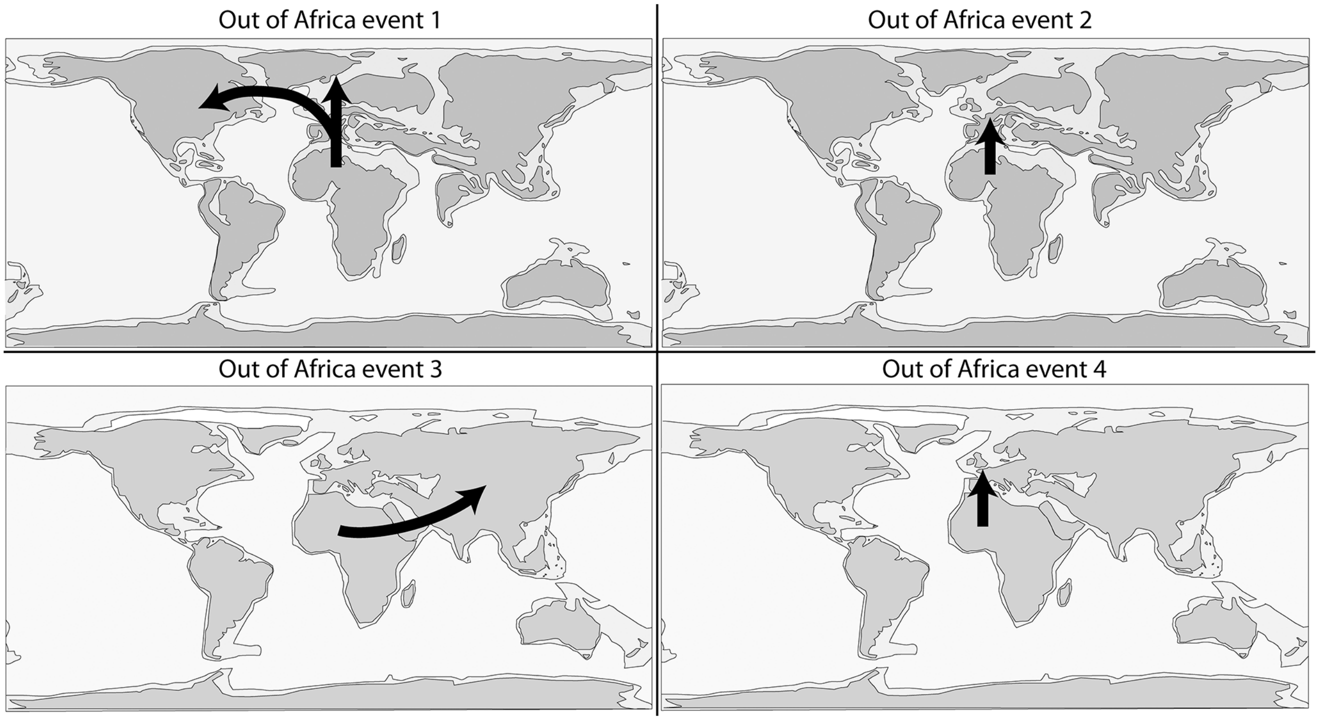

Fig 20. Illustration of the different dispersal events supported by the hyainailourines' distributions. Out of Africa event 1 = Lutetian/Bartonian Dispersal Phase; Out of Africa event 2 = Bartonian/Priabonian Dispersal Phase; Out of Africa event 3 = MN3 Dispersal Phase; Out of Africa event 4 = MN4 Dispersal phase. (Global map for OoA-1 and OoA-2 is adapted from Ron Blakey, Eocene, http://www2.nau.edu/rcb7/050Marect.jpg and global map for OoA-3 and OoA-4 is adapted from Ron Blakey, Miocene http://jan.ucc.nau.edu/rcb7/020Marect.jpg).

doi:10.1371/journal.pone.0135698.g020

exchange, we propose that the Bartonian hyainailourines may have entered into Europe through the Iberian Peninsula.

The presence of *Hemipsalodon* in the Duchesnean NALMA is very interesting. It appeared together with other new migrants. Emry [132] noted that the artiodactyls *Brachyhyops* and *Simimeryx*, as well as the hyaenodontine *Hyaenodon*, were probably migrants from Asia during the Duchesnean. Because *Hemipsalodon* is not present in all Duchesnean localities, it has been used as an index taxon for separating early and late Duchesnean with *Hemipsalodon* not present in the early Duchesnean. Because *Hemipsalodon* and *Hyaenodon* do not appear in North American localities at the same time, and because there is a close relationship between *Paroxyaena* and *Hemipsalodon* (see the phylogenetic analysis), the Paroxyaenini may have dispersed from Europe to North America during the late Duchesnean (Fig 20).

Concerning this issue, it should be emphasized that *Orienspterodon* displays several features that are reminiscent of *Paroxyaena* such as the presence of cingulids on premolars. However, the Asian species differ from *Paroxyaena* in double-rooted P^3, better fusion of paracone and metacone on M^1, small metaconid on M_3 and vestigial metaconid on M_{1-2} [128]. A close relationship between *Orienspterodon* and Paroxyaenini would support a dispersal of these hyainailourines to Asia. Such a dispersal represents a competing hypothesis to the dispersal of Paroxyaenini in North America (i.e., a dispersal from Asia to North America as reconstructed for the hyaenodontines). Only the discovery of new material for *Orienspterodon* will allow its systematic position to be precisely tested and further discussion of these paleobiogeographic scenarios.

The presence of the hyainailourine taxa "*Pterodon*"and *Parapterodon* in the Late Eocene of Europe has previously been considered support for a Bartonian/Priabonian Dispersal Phase [6] (OoA-2 on Figs 19 and 20). As noted by Gheerbrant & Rage [129], the Bartonian/Priabonian Dispersal Phase is characterized by the immigration of the "baluchimyine" rodent *Protophiomys* into Africa. Consequently, the hyainailourines could represent the sole case of a northward dispersal during this phase.

The hypothesized existence of two possible dispersal events results from the lack of support for endemic diversification of the hyainailourines in Europe during the Bartonian and Priabonian. It also implies a turnover, which affected the Europe hyainailourines between these stages. This could result from the inability of hyainailourines to cope with some aspects of European environments. Indeed, "*Pterodon*" also rapidly disappeared from Europe and is only recorded from MP18-MP20. This is especially conspicuous when compared to the extensive diversification and long stratigraphic record of European *Hyaenodon* and development of a separate lineage within Europe [133].

Unlike Apterodontinae [66], the hyainailourines did not disperse to Laurasia during the Oligocene, but did so during the Miocene. Two distinct phases can be defined. The OoA-3 corresponds to a migration from Africa to Asia. It resulted in the presence of *Hyainailouros* and *Sivapterodon* in Asia (Figs 19 and 20). This dispersal occurred at 19.6 Ma (Mammal Neogene level, MN3). The second dispersal of *Hyainailouros* occurred only slightly later, when it dispersed to Europe at MN4 (Figs 19 and 20).

By the Early-Middle Miocene, the widely dispersed *Hyainailouros* and African *Isohyaenodon* were the last representatives of hyainailourines. Their size differences show that this group remained ecologically and morphologically diverse. The only other hyaenodonts known at that time were the teratodontine *Dissopsalis*, which was present in Africa and Asia [134] and the koholiine *Metapterodon*, which was possibly present in Asia [135]. These four taxa are the swan song of Hyaenodonta and they witnessed the ultimate disappearance of their once diverse and successful order.

Conclusion

In this contribution we describe a new hyainailourid hyainailourine, *Kerberos langebadreae* gen. & sp. nov., based on a skull, mandible, and a few elements of the hind limb. Associated dental and cranial or postcranial elements of hyaenodonts is rare, especially among the hyainailourines, making this description of one of the oldest known hyainailourines particularly important for understanding the evolution of Hyaenodonta.

The skull of *Kerberos* already displays features typical of the hyainailourines–and by extension of the Hyainailouridae. The hind limb of *Kerberos* exhibits features consisted with a preference for terrestrial substrates and a plantigrade posture. Because of its size and the absence of adaptations to cursoriality, the new taxon was probably not only an active predator, but also a successful opportunistic scavenger.

The powerful masticatory musculature supports the scavenger behavioral hypothesis usually evoked for Miocene hyainailourines. This contrasts with the contemporaneous *Hyaenodon*. These hyaenodontines, which probably originated in Asia and were represented by numerous species in Europe [9,136], exhibit terrestrial (semi-plantigrady/digitigrady) and cursorial postcranial features [33,74,137]. *Kerberos* was ecologically more similar to the striped and spotted hyenas than to contemporaneous hyaenodontines except that *Kerberos* lacked the cursorial adaptations of these extant hyaenids.

The respective endemic evolution of hyaenodontines (in Asia) and hyainailourines (in Africa) explains both similarities and differences between these two clades, which became

competitors in Europe and North America, but our knowledge of this history still needs to be improved.

Supporting Information

S1 Text. Taxa included in the phylogenetic analysis–except *Parvavorodon*–and their estimated body mass. *Category established by comparisons with relatives of similar sizes.
(DOCX)

S2 Text. Character list.
(DOCX)

S1 File. Data matrix in nexus format.
(ZIP)

Acknowledgments

The optical photographs were taken by C. Lemzaouda and P. Loubry (CNRS). We thank C. Argot, P. Tassy, G. Billet, and C. Sagne (MNHN) for granting access to the material of Hyaenodonta under their care. We are also indebted to C. Bouillet (MNHN) for helping preparing the skull of the holotype of *K. langebadreae*. We thank R. Tabuce (CNRS-Université Montpellier 2) for having permitted the utilization of the picture of *Furodon crocheti*. We also thank Shawn P. Zack, Lars Werdelin, Gregg Gunnell, Margaret E. Lewis and one anonymous referee and the Academic Editors–Nathan Jeffery and Matt Friedman–for their very constructive comments that improved the manuscript.

Author Contributions

Conceived and designed the experiments: FS EA MB DV MM KB. Performed the experiments: FS EA MB DV MM KB. Analyzed the data: FS EA MB DV MM KB. Contributed reagents/materials/analysis tools: FS EA MB DV MM KB. Wrote the paper: FS EA MB DV MM KB.

References

1. Solé F, Gheerbrant E, Amaghzaz M, Bouya B (2009) Further evidence of the African antiquity of hyaenodontid ("Creodonta", Mammalia) evolution. Zoological Journal of the Linnean Society 156: 827–846.
2. Yans J, Amaghzaz M, Bouya B, Cappetta H, Iacumin P, Kocsis L, et al. (2014) First carbon isotope chemostratigraphy of the Ouled Abdoun phosphate Basin, Morocco; implications for dating and evolution of earliest African placental mammals. Gondwana Research 25: 257–269.
3. Savage RJG (1965) Fossil mammals of Africa: 19 The Miocene Carnivora of East Africa. Bulletin of the British Museum (Natural History), Geology 10: 242–316.
4. Ginsburg L (1980) *Hyainailouros sulzeri*, mammifère créodonte du Miocène d'Europe. Annales de Paléontologie (Vertébrés) 66: 19–73.
5. Gheerbrant E (1995) Les mammiferes paleocenes du Bassin d'Ouarzazate (Maroc) III. Adapisoriculidae et autres mammiferes (Carnivora,? Creodonta, Condylarthra,? Ungulata et incertae sedis). Palaeontographica Abteilung A 237: 39–132.
6. Solé F, Lhuillier J, Adaci M, Bensalah M, Mahboubi M, Tabuce R (2013b). The hyaenodontids from the Gour Lazib area (?Early Eocene, Algeria): implications concerning the systematics and the origin of the Hyainailourinae. Journal of Systematic Palaeontology 12, 303–322.
7. Meng J, Zhai R-J, Wyss AR (1998) The late Paleocene Bayan Ulan fauna of Inner Mongolia, China. Bulletin of Carnegie Museum of Natural History 34: 148–185.
8. Lavrov AV (2007) A new species of *Paroxyaena* (Hyaenodontidae, Creodonta) from Phosphorites of Quercy, Late Eocene, France. Paleontological Journal 41: 298–311

9. Lange-Badré B (1979) Les Créodontes (Mammalia) d´Europe occidentale de l´Éocéne supérieur à l´Oligocéne supérieur. Mémoires du muséum national d´histoire naturelle, Série C, Sciences de la terre 42: 1–249.
10. Ogg JG, Lugowski A (2013) TS creator–visualization of enhanced Geologic Time Scale 2012 database. Available: http://www.tscreator.org.
11. Gradstein FM, Ogg JG, Schmitz M, Ogg G (2012) The geologic time scale 2012, 2-Volume set. Elsevier, Amsterdam. 1144 pp.
12. Smith T, Smith R (2001) The creodonts (Mammalia, Ferae) from the Paleocene-Eocene transition in Belgium (Tienen Formation, MP7). Belgian Journal of Zoology 131: 117–136.
13. Solé F, Gheerbrant E, Godinot M (2013a) The Sinopaninae and Arfianinae (Hyaenodontida, Mammalia) from the Early Eocene of Europe and Asia; evidence for dispersals in Laurasia around the P/E boundary and for an unnoticed faunal turnover in Europe. Geobios 46: 313–327.
14. Hooker JJ (2010) The mammal fauna of the Early Eocene Blackheath Formation of Abbey Wood, London. Monograph of the Palaeontographical Society London 165: 1–162.
15. Godinot M (1981) Les mammifères de Rians (Eocène inférieur, Provence). Palaeovertebrata 10: 43–126.
16. Godinot M, Crochet J-Y, Hartenberger J-L, Lange-Badré B, Russell DE, Sigé B (1987) Nouvelles données sur les mammifères de Palette (Eocène inférieur, Provence). Münchner Geowissenschaftliche Abhandlungen A 10: 273–288.
17. Solé F, Falconet J, Laurent Y (2014) New Proviverrines (Hyaenodontida) from the Early Eocene of Europe; Phylogeny and Ecological Evolution of the Proviverrinae. Zoological Journal of the Linnean Society 171: 878–917.
18. Marandat B (1997) La disparité des faunes mammaliennes du niveau MP 7 (Eocène inférieur) des domaines péri-mésogéens et nordiques. Investigation d'un provincialisme intra-européen. Newsletters on Stratigraphy 35: 63–82.
19. Marandat B, Adnet S, Marivaux L, Martinez A, Vianey-Liaud M, Tabuce R (2012) A new mammalian fauna from the earliest Eocene (Ilerdian) of the Corbières (Southern France): palaeobiogeographical implications. Swiss Journal of Geosciences 105: 417–434.
20. Morlo M, Gunnell GF, Nagel D (2010). Chapter 10 –Ecomorphological analysis of carnivore guilds in the Eocene through Miocene of Laurasia. In Goswami A, Friscia A, editors. New contributions to the Natural History of Carnivora. Cambridge University Press, Cambridge, U.K. pp. 269–310.
21. Morlo M (1999) Niche structure and evolution in creodont (Mammalia) faunas of the European and North American Eocene. Geobios 32: 397–305.
22. Solé F (2014) New carnivoraforms from the early Eocene of Europe and their bearing on the evolution of the Carnivoraformes. Palaeontology 57: 963–978.
23. Solé F, Gheerbrant E, Godinot M (2011). New data on the Oxyaenidae from the Early Eocene of Europe; biostratigraphic, paleobiogeographic and paleoecologic implications. Palaeontologia Electronica: 14. Available: http://palaeo-electronica.org/2011_2/258/index.html. Accessed July 2011
24. Morlo M, Habersetzer J (1999) The Hyaenodontidae (Creodonta, Mammalia) from the lower Middle Eocene (MP11) of Messel (Germany) with special remarks on new x-ray methods. Courier Forschungsinstitut Senckenberg 216: 31–73.
25. Laurent Y, Adnet S, Bourdon E, Corbalan D, Danilo L, Duffaud S, et al. (2010) La Borie (Saint- Papoul, Aude): un gisement exceptionnel dans l'Éocène basal du Sud de la France. Bulletin de la Société d'Histoire Naturelle de Toulouse 146: 89–103.
26. Stehlin HG (1904) Sur les Mammifeh special reference to the appearance of Hunter-Schreger-Bété géologique de France, 4e serie 4: 445–475.
27. Matthis C (1985) Contribution à la connaissance des mammifères de Robiac (Eocène supérieur): Creodonta et Carnivora. Bulletin du Muséum National d'Histoire Naturelle; Paris, 4e série, 7, 305–326.
28. Sudre J (1969) Les gisements de Robiac (Eocène supérieur) et leurs faunes de Mammifères. Palaeovertebrata 2: 95–156.
29. Astruc JG, Hugueney M, Escarguel G, Legendre S, Rage J-C, Simon-Coicon R, et al. (2003) Puycelci, nouveau site à vertébrés de la série molassique d'Aquitaine. Densité et continuité biochronologique dans la zone Quercy et bassins périphériques au Paléogène. Géobios 36: 629–648
30. BiochroM'97 (1997). Synthèses et tableaux de correlation. In Aguilar J-P, Legendre S and Michaux J, editors, Actes du Congrès BiochroM'97 Montpellier. Mémoires et Travaux de l'Ecole Pratique des Hautes Etudes, Institut de Montpellier, 21. pp. 769–805

31. Lange-Badré B (1978) *Cynohyaenodon lautricensis* nov. sp. (Creodonta, Mammalia) et les *Cynohyaenodon* européens. Bulletin de la Société d'Histoire Naturelle de Toulouse 114: 472–483.

32. Van Valen L (1966) Deltatheridia, a new order of mammals. Bulletin of the American Museum of Natural History 132: 1–126.

33. Ginsburg L (1999) Order Carnivora. In Rössner GE, Heissig K, editors. The Miocene Land mammals of Europe. Friedrich Pfeil, München, Germany. pp. 105–108.

34. Gingerich PD, Deutsch HA (1989) Systematics and evolution of Early Eocene Hyaenodontidae (Mammalia, Creodonta) in the Clarks Fork Basin, Wyoming. Contributions from the Museum of Paleontology, the University of Michigan 27: 327–391.

35. Miller ME, Christensen G, Evans H (1965) Anatomy of the Dog. Academic Medicine, Philadelphia, Saunders. p. 941

36. Argot C (2004) Functional-adaptative features and palaeobiologic implications of the postcranial skeleton of the late Miocene sabretooth borhyaenid *Thylacosmilus atrox* (Metatheria). Alcheringa 28: 229–266.

37. Holroyd PA (1994) An examination of dispersal origins of Fayum mammalia. PhD. Thesis. Durham, Duke University. 328 p.

38. Polly PD (1996) The skeleton of *Gazinocyon vulpeculus* gen. et comb. nov. and the cladisitic relationships of Hyaenodontidae (Eutheria, Mammalia). Journal of Vertebrate Paleontology 16: 303–319.

39. Nixon KC (1999) *Winclada (BETA) ver 0.9.9*. Published by the author, Ithaca, NY.

40. Goloboff PA, Farris JS, Nixon KC (2008) TNT, a free program for phylogenetic analysis. Cladistics 24: 774–786.

41. Morales J, Pickford M, Fraile S, Salesa MJ, Soria D (2007) New carnivoran material (Creodonta, Carnivora and Incertae sedis) from the early Miocene of Napak, Uganda. Paleontological Research 11: 71–84.

42. Owen R (1837) Teeth. In Todd RB, editor. The Cyclopaedia of Anatomy and Physiology, 4(2). Sherwood, Gilbert, and Piper, London, U.K. pp. 864–935.

43. Linnaeus C (1758) Systema Naturae per regna tria naturae, secundum classes, ordines, genera, species, cum characteribus, differentiis, synonymis, locis. Editio decima, reformata. Laurentius Salvius: Holmiae. ii, 824 pp.

44. Van Valen L (1967) New Paleocene insectivores and insectivore classification. Bulletin of the American Museum of Natural History 135: 217–284.

45. Gunnell GF (1998) Creodonta. In: Janis CJ, Scott KM, Jacobs LL, editors. Evolution of tertiary mammals of North America–Volume 1: Terrestrial Carnivores, Ungulates, and ungulatelike mammals. Cambridge University Press. pp. 91–109.

46. Leidy J (1869) On the extinct Mammalia of Dakota and Nebraska. Journal of the Academy of Natural Sciences Philadelphia 7: 1–472

47. Pilgrim GE (1932) The fossil Carnivora of India. Memoirs of the Geological Survey of India, Palaeontologia indica 18: 1–232.

48. Crochet J-Y (1988) Le plus ancien Créodonte africain: *Koholia atlasense* nov. gen., nov. sp. (Eocene inferieur d'El Kohol, Atlas saharien, Algerie). Comptes Rendus de l'Academie des Sciences, Paris 307: 1795–1798.

49. Polly PD (1994) What if anything is a creodont? Journal of Vertebrate Paleontology 14(supplement): 42A.

50. Gheerbrant E, Iarochene M, Amaghzaz M, Bouya B (2006) Early African hyaenodontid mammals and their bearing on the origin of the Creodonta. Geological Magazine 134: 475–489.

51. Solé F (2013) New proviverrine genus from the Early Eocene of Europe and the first phylogeny of Late Paleocene-Middle Eocene hyaenodontidans (Mammalia). Journal of Systematic Paleontology 11: 375–398.

52. Grohé C, Morlo M, Chaimanee Y, Blondel C, Coster P, Valentin X, et al. (2012) New apterodontinae (Hyaenodontida) from the Eocene locality of Dur At-Talah (Libya): systematic, paleoecological and phylogenetical implications. PLOS One 7:e49054. doi: 10.1371/journal.pone.0049054 PMID: 23185292

53. Morlo M, Bastl K, Wenhao W, Schaal SFK (2014) The first species of *Sinopa* (Hyaenodontida, Mammalia) from outside of North America: Implications for the history of the genus in the Eocene of Asia and North America. Palaeontology 57: 111–125.

54. Spaulding M, O'Leary MA, Gatesy J (2009) Relationships of Cetacea (Artiodactyla) Among Mammals: Increased Taxon Sampling Alters Interpretations of Key Fossils and Character Evolution. PLoS ONE 4(9): e7062. doi: 10.1371/journal.pone.0007062 PMID: 19774069

55. Matthew WD (1906) The osteology of *Sinopa*, a creodont mammal of the Middle Eocene. Proceedings of the United States National Museum 30: 203–233.
56. Osborn HF (1909) New carnivorous mammals from the Fayûm Oligocene, Egypt. Bulletin of the American Museum of Natural History 26: 415–424.
57. Szalay FS (1967) The affinities of *Apterodon* (Mammalia, Deltatheridia, Hyaenodontidae). American Museum Novitates 2293: 1–17.
58. Matthew WD (1909) The carnivora and Insectivora of the Bridger Basin, middle Eocene. Memoirs of the Amercian Museum of Natural History 9: 289–567.
59. Colbert EH (1933) The skull of *Dissopsalis carnifex* Pilgrim, a Miocene creodont from India. American Museum Novitates 603: 1–8
60. Egi N, Holroyd PA, Tsubamoto T, Soe AN, Takai M, Ciochon RL (2005) Proviverrine Hyaenodontids (Creodonta: Mammalia) from the Eocene of Myanmar and a phylogenetic analysis of the Proviverrines from the Para-Tethys Area. Journal of Systematic Paleontology 3: 337–358.
61. Rana R, Kumar K, Zack S, Solé F, Rose KD, Missiaen P, et al. (2015) Craniodental and postcranial morphology of *Indohyaenodon raoi* from the early Eocene of India, and its implications for ecology, phylogeny, and biogeography of hyaenodontid mammals. Journal of vertebrate paleontology. doi: 10.1080/02724634.2015.965308
62. Denison RH (1938) The broad skull Pseudocreodi. Annals of the New York Academy of Sciences 37: 163–257.
63. Muizon C, Lange-Badré B (2007) Carnivorous dental adaptations in tribosphenic mammals and phylogenetic reconstruction. Lethaia 30: 353–366.
64. Shawn Z (2014) Saber-tooth origins: a new skeletal association and the affinities of Machaeroidinae (Mammalia, Creodonta). Journal of Vertebrate Paleontology, Program and Abstracts, 2014: 259–260.
65. Mellett JS (1969) A skull of *Hemipsalodon* (Mammalia, Deltatheridia) from the Clarno Formation of Oregon. American Museum Novitates 387: 1–19
66. Lange-Badré B, Böhme M (2005) *Apterodon intermedius*, sp. nov., a new European creodont mammal from MP22 of Espenhain (Germany). Annales de Paléontologie 91: 311–328.
67. Wortman JL (1902) Studies of Eocene Mammalia in the Marsh collection, Peabody Museum. Part I. Carnivora. Paper 7. American Journal of Science 13: 197–206
68. Schlosser M (1886) Paläontologische Notizen. Über das Verhältniss der Cope'schen Creodonta zu den ubrigen Fleischfressem. Morphologische Jahrbüch 12: 287–294.
69. Holroyd PA (1999) New Pterodontinae (Creodonta:Hyaenodontidae) from the late Eocene-early Oligocene Jebel Qatrani Formation, Fayum province, Egypt. Paleobios 19: 1–18.
70. Lewis ME, Morlo M (2010) Creodonta. In Werdelin L, Sanders WJ, editors. Cenozoic mammals of Africa. University of California Press. pp. 543–560.
71. Frey E, Munk W, Böhme M, Morlo M, Hensel M (2011) First creodont carnivore from the Rupelian Clays (Oligocene) of the Clay Pit Unterfeld at Rauenberg (Rhein-Neckar-Kreis, Baden-Würtemberg): *Apterodon rauenbergensis* n.sp. Kaupia 17: 107–113.
72. Werdelin L, Solounias N (1991) The Hyaenidae: taxonomy, systematics and evolution. Fossils and Strata 30: 1–104.
73. Wang X, Tedford RH, Taylor BE (1999) Phylogenetic systematics of the Borophaginae (Carnivora, Canidae). Bulletin of the American Museum of Natural History 243: 1–391.
74. Cope ED (1885) The White River beds of Swift Current River, Northwest Territory. American Naturalist, 19: 163.
75. Biedermann WGA (1863) Petrefacten aus der Umgegend von Winterthur: II. Die Braunkohlen von Elgg. Anhang: *Hyainailouros sulzeri*. Bleuler-Hausheer, Wintherthur. 23 pp.
76. Martin R (1906) Revision der obereocaenen und unteroligocaenen Creodonten Europas. Revue Suisse de Zoologie 14: 405–600.
77. Blainville HMD de. (1839). Sur l'*Hyaenodon leptorhynchus* (de Laizer) nouveau genre de carnassiers fossiles d'Auvergne. Annales Françaises et Etrangères d'Anatomie et Physiologie, 3: 17–31.
78. Morlo M, Miller ER, El-Barkooky AN (2007) Creodonta and Carnivora from Wadi Moghra, Egypt. Journal of Vertebrate Paleontology 27: 145–159.
79. Pilgrim GE (1912) The Vertebrate Fauna of the Gaj Series in the Bugti Hills and the Punjab. Memoirs of the Geological Survey of India. Palaeontologia Indica, New Series 4:1–83.
80. Savage RJG (1973) *Megistotherium*, gigantic hyaenodont from Miocene of Gebel Zelten, Libya. Bulletin of the British Museum of Natural History (Geology) 22: 483–511.

81. Koenigswald GHR von (1947) Ein *Hyaenaelurus* aus dem Miocaen Nordafrikas. Société Paléontologique de Suisse, Spécial Volume: Contribution à l'Étude des Vertébrés miocène de l'Egypte: 292–294.
82. Pasitschniak-Arts M (1993) Ursus arctos. Mammalian Species 439: 1–10.
83. Mellett JS (1977) Paleobiology of North American *Hyaenodon* (Mammalia, Creodonta). Contributions to Vertebrate Evolution 1: 1–133.
84. Andrews CW (1903) Notes on expedition to the Fayum, Egypt, with descriptions of some new mammals. Geological Magazine 4: 337–343
85. Crochet J-Y, Remy J-A, Sudre J (1983) Données nouvelles sur la faune mammalienne de Fons 6 (Fons outre Gardon, Gard) datée de l'Eocène supérieur. Géologie Méditerranéenne 10: 49–62.
86. Van Valkenburgh B (1999) Major patterns in the history of carnivorous mammals. Annual Review of Earth and Planetary Science 27: 463–493.
87. Gittleman JL (1985) Carnivore body size: Ecological and taxonomic correlates. Oecologia 67: 540–554.
88. Carbone C, Teacher A, Rowcliffe JM (2007) The costs of carnivory. PLoS Biol 5(2): e22. doi: 10.1371/journal.pbio.0050022 PMID: 17227145
89. Van Valkenburgh B (1987) Skeletal indicators of locomotor behaviour in living and extinct carnivores. Journal of Vertebrate Paleontology 7: 162–182.
90. Gingerich PD (1990) Prediction of body mass in mammalian species from the long bone lengths and diameters. Contributions from the Museum of Paleontology 28: 79–92.
91. Egi N (2001) Body mass estimates in extinct mammals from limb bone dimensions: the case of North American hyaendontids. Paleontology 44: 497–528.
92. Tsubamoto T (2014) Estimating body mass from the astragalus in mammals. Acta Palaeontologica Polonica 59: 259–265.
93. Rémy JA, Crochet J-Y, Sigé B, Sudre J, de Bonis L, Vianey-Liaud M, Godinot M, Hartenberger J-L, Lange-Badré B, Comte B (1987) Biochronologie des phosphorites du Quercy: mise à jour des listes fauniques et nouveaux gisements de mammifères fossiles.Münchner geowissenschaftliche Abhandlungen A 10: 169–188.
94. Turnbull WD (1970) Mammalian masticatory apparatus. Fieldiana, Geology 18: 149–356.
95. Ewer RF (1973) The carnivores. Cornell University Press, Ithaca, New York
96. Smith KK (1993) The form of the feeding apparatus in terrestrial vertebrates: studies of adaptation and constraint. In Hanken J, Hall BK, editors. The skull—functional and evolutionarymechanisms, Vol. 3. University of Chicago Press, Chicago, USA. pp. 150–196
97. Laison F, Lautrou A, Azérad J, Pollin B, Lévy G (2001) Superficial architecture of the jaw-closing muscles of the cat (*Felis catus*): the temporo-masseteric complex. Comptes Rendus de l'Académie des Sciences, Séries III 324: 855–862.
98. Greaves WS (1985). The generalized carnivore jaw. Zoological Journal of the Linnean Society 85: 267–274.
99. Filan SL (1990) Myology of the head and neck of the bandicoot marsupialia peramelemorphia. Australian Journal Of Zoology 38: 617–634.
100. Antón M, Salesa MJ, Pastor JF, Sánchez IM, Fraile S, Morales J. 2004. Implications of the mastoid anatomy of larger extant felids for the evolution and predatory behaviour of Sabretoothed cats (Mammalia, Carnivora, Felidae). Zoological Journal of the Linnean Society 140: 207–221.
101. Stefen C (1997) The enamel of Creodonta, Arctocyonidae, and Mesonychidae (Mammalia), with special reference to the appearance of Hunter-Schreger-Bands. Paläontologische Zeitschrift 71: 291–303
102. Bastl K, Semprebon G, Nagel D (2012) Low-magnification microwear in Carnivora and dietary diversity in Hyaenodon (Mammalia: Hyaenodontidae) with additional information on its enamel microstructure. Palaeogeography, Palaeoclimatology, Palaeoecology 348–349: 13–20.
103. Werdelin L (1989) Constraint and adaptation in the bone-cracking canid *Osteoborus* (Mammalia, Canidae). Paleobiology 15: 387–401.
104. Barnet CH, Napier JR (1953) The rotary mobility of the fibula in eutherian mammals. Journal of Anatomy 87: 11–21. PMID: 13022578
105. Bastl K (2012) The ecomorphology of the European *Hyaenodon*. Ph.D. Thesis. University Vienna. 300 pp.
106. Ginsburg L (1961) Plantigradie et digitigradie chez les carnivores fissipedes. Mammalia 25: 1–21.

107. Laborde C (1987) Caractéres d´adaption des membres au mode de vie aboricole chez *Cryptoprocta ferox* par comparaison d´autres Carnivores Viverridés. Annales des Sciences Naturelles, Zoologie, Série 13: 25–39.

108. O'Leary MA, Rose KD (1995) Postcranial skeleton of the early eocene mesonychid *Pachyaena* (Mammalia: Mesonychia). Journal of Vertebrate Paleonto1ogy 15: 401–430.

109. Heinrich RE, Rose KD (1997) Postcraniam morphology and locomotor behavior of two Early Eocene miacoid carnivorans, *Vulpavus* and *Didymictis*. Palaeontology 40: 279–305.

110. O'Leary MA, Bloch JI, Flynn JJ, Gaudin TJ, Giallombardo A, Giannini NP, et al. (2013) The Placental Mammal Ancestor and the Post–K-Pg Radiation of Placentals. Science 339: 662–667 doi: 10.1126/science.1229237 PMID: 23393258

111. Gould FHD, Rose KD (2014) Gnathic and postcranial skeleton of the largest known arctocyonid "condylarth," *Arctocyon mumak*, (Mammalia, Procreodi) and ecomorphological diversity in Procreodi. Journal of Vertebrate Paleontology 34: 1180–1202.

112. Sinclair WJ, Jepsen GL (1929) A mounted skeleton of *Palaeonictis*. Proceedings of the American Philosophical Society 68: 163–173.

113. Rose KD (2001) Compendium of Wasatchian mammal postcrania from the Willwood Formation of the Bighorn Basin. University of Michigan Papers on Paleontology 33:157–183.

114. Gebo DL, Rose KD (1993) Skeletal morphology and locomotor adaptation in *Prolimnocyon atavus*, an early Eocene hyaenodontid creodont. Journal of Vertebrate Paleontology 13:125–144.

115. Morlo M, Gunnell GF (2003) Small Limnocyonines (Hyaenodontidae, Mammalia) from the Bridgerian Middle Eocene of Wyoming: *Thinocyon*, *Prolimnocyon*, and *Iridodon*, new genus. Contributions from the Museum of Paleontology, The University of Michigan 31: 43–78.

116. Carbone C, Mace GM, Roberts SC, Macdonald DW (1999) Energetic constraints on the diet of terrestrial carnivores. Nature 402: 286–288 PMID: 10580498

117. Van Valkenburgh V (1985) Locomotor diversity in past and present guilds of large predatory mammals. Paleobiology 11: 406–428

118. Janis CM, Wilhelm PB (1993) Were there mammalian pursuit predators in the Tertiary? Dances with wolf avatars. Journal of Mammalian Evolution 1: 103–125.

119. Peigné S, Morlo M, Chaimanee Y, Ducrocq S, Tun ST, Jaeger J-J (2007) New discoveries of hyaenodontids (Creodonta, Mammalia) from the Pondaung Formation, middle Eocene, Myanmar—paleobiogeographic implications. Geodiversitas 29: 441–458.

120. Gustafson EP (1986) Carnivorous mammals from the late Eocene and early Oligocene of Trans-Pecos, Texas. Texas Memorial Museum Bulletin 33: 1–66.

121. Rasmussen DT, Gutierrez M (2009) A mammalian fauna from the Late Oligocene of northwestern Kenya. Palaeontographica Abteilung A 288: 1–52.

122. Rasmussen DT, Tilden CD, Simons EL (1989) New specimens of the gigantic creodont, *Megistotherium*, from Moghara, Egypt. Journal of Mammalogy 70: 442–447.

123. Van der Made J, Mazo AV (2003) Proboscidean dispersals from Africa towards Western Europe. Deinsea 9: 437–452.

124. Gheerbrant E, Tassy P (2009) L'origine et l'évolution des éléphants. Comptes-Rendus Palevol 8: 281–294.

125. Sander WJ, Gheerbrant E, Harris JH, Saegusa H, Delmer C (2010) Proboscidea. In Werdelin L, Sanders WJ, editors. Cenozoic mammals of Africa. University of California Press. pp. 161–251.

126. Cardini A, Polly PD (2013) Larger mammals have longer faces because of size-related constraints on skull form. Nature Communications 4: 2458. doi: 10.1038/ncomms3458 PMID: 24045342

127. Van Valkenburgh B, Wang X, Damuth J (2004) Cope's rule, hypercarnivory, and extinction in North American canids. Science 306: 101–104. PMID: 15459388

128. Egi N, Tsubamoto T, Takai M (2007) Systematic status of Asian "*Pterodon*" and early evolution of hyaenaelurine hyaenodontid creodonts. Journal of Paleontology 81: 4770–4778.

129. Gheerbrant E, Rage J-C (2006) Paleobiogeography of Africa: How distinct from Gondwana and Laurasia? Palaeogeography, Palaeoclimatology, Palaeoecology 241: 224–246.

130. Franzen JL (2003) Mammalian faunal turnover in the Eocene of central Europe. GSA Special Papers 369: 455–461.

131. Badiola A, Checa L, Cuesta MA, Quer R, Hooker JJ, Astibia H (2009) The role of new Iberian finds in understanding European Eocene mammalian paleobiogeography. Geologica Acta 7: 243–258.

132. Emry RJ (1981) Additions to the mammalian fauna of the type Duchesnean, with comments on the status of the Duchesnean "Age." Journal of Paleontology 55: 563–570.

133. Bastl K, Nagel D, Peigné S (2014) Milk tooth morphology of small-sized *Hyaenodon* (Hyaenodontidae, Mammalia) from the European Oligocene–evidence of a *Hyaenodon* lineage in Europe. Palaeontographica A 303: 61–84.

134. Barry JC (1988) *Dissopsalis*, a middle and late Miocene proviverrine creodont (Mammalia) from Pakistan and Kenya. Journal of Vertebrate Paleontology 8: 25–45.

135. Barry JC (1980) Occurrence of a Hyaenodontine Creodont (Mammalia) in the Late Miocene of Pakistan. Journal of Paleontology 54: 1128–1131.

136. Bastl K, Morlo M, Nagel D, Heizmann E (2011) Differences in the tooth eruption sequence in *Hyaenodon* ("Creodonta", Mammalia) and implications for the systematics of the genus. Journal of Vertebrate Paleontology 31: 181–192.

137. Scott WB, Jepsen G (1936) The Mammalian fauna of the White River Oligocene. Part I–Insectivora and Carnivora. Transactions of the American Philosophical Society 28: 1–153.
